# The Effect of Pyridine-2-thiolate Ligands on the Reactivity of Tungsten Complexes toward Oxidation and Acetylene Insertion

**DOI:** 10.1021/acs.organomet.1c00472

**Published:** 2021-10-18

**Authors:** Riccardo Bondi, Miljan Z. Ćorović, Michael Buchsteiner, Carina Vidovič, Ferdinand Belaj, Nadia C. Mösch-Zanetti

**Affiliations:** † Institute of Chemistry, Inorganic Chemistry, University of Graz, 8010 Graz, Austria

## Abstract

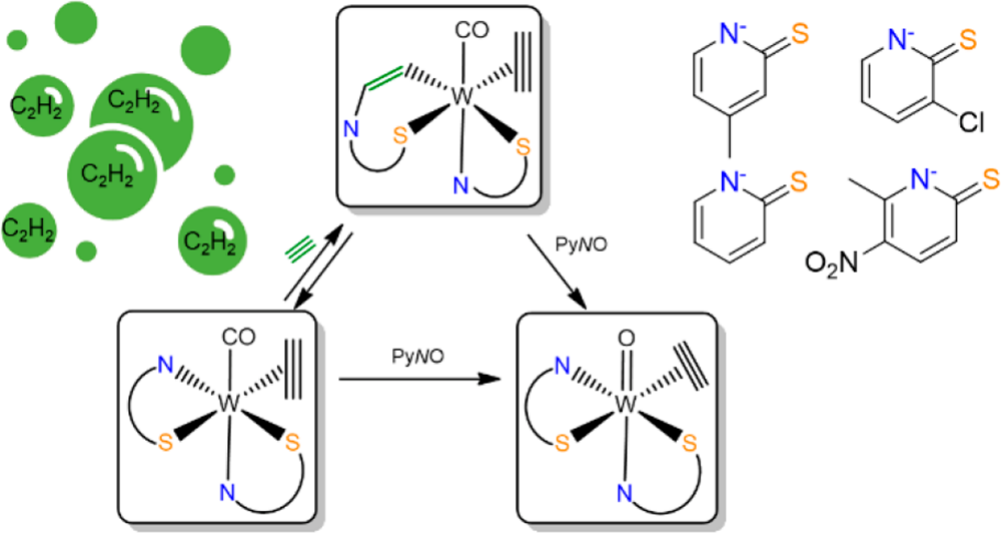

Intending to deepen our understanding of tungsten acetylene (C_2_H_2_) chemistry, with regard to the tungstoenzyme acetylene hydratase, here we explore the structure and reactivity of a series of tungsten acetylene complexes, stabilized with pyridine-2-thiolate ligands featuring tungsten in both +II and +IV oxidation states. By varying the substitution of the pyridine-2-thiolate moiety with respect to steric and electronic properties, we examined the details and limits of the previously reported intramolecular nucleophilic attack on acetylene followed by the formation of acetylene inserted complexes. Here, we demonstrate that only the combination of high steric demand and electron-withdrawing features prevents acetylene insertion. Nevertheless, although variable synthetic approaches are necessary for their synthesis, tungsten acetylene complexes can be stabilized predictably with a variety of pyridine-2-thiolate ligands.

## Introduction

Tungsten is the metal of choice for several challenging enzymatic reactions.^1^ Besides being in the active site of the metalloenzymes that catalyze redox reactions, it is also essential for the function of acetylene hydratase (AH). This is a unique example of a tungstoenzyme catalyzing the nonredox hydration of acetylene to acetaldehyde.^1−5^ This reaction is the first metabolic step of the mesophilic bacterium *Pelobacter acetylenicus*, which consumes acetylene as its only carbon and energy source.^2^ The only other known enzyme that accepts acetylene as a substrate is nitrogenase, which reduces acetylene to ethylene.^6^ The mechanism of the catalysis of AH remains elusive, as there are no reported crystal structures of the enzyme containing substrate or any inhibitor. Since one of the mechanistic ideas of AH suggests coordination of acetylene to the tungsten(IV) center,^7−12^ we aim to synthesize and understand tungsten acetylene adducts with ligands similar to those in tungstoenzymes.^13,14^

The first tungsten(II) acetylene species bearing dithiocarbamate ligands was reported in 1978;^15^ thereafter, only a few other W–C_2_H_2_ adducts have been synthesized.^16−26^ For example, Templeton et al. reported a tungsten(IV) oxo acetylene complex containing an N-donor boron-based scorpionate ligand.^20^ In our group, the use of bioinspired S,N-bidentate ligands, such as SPhoz (2-(4′4′-dimethyloxazoline-2′-yl)thiophenolate),^21^ PyS (pyridine-2-thiolate),^24^ and 6-MePyS (6-methylpyridine-2-thiolate),^26^ allows the preparation of W(II) and W(IV) complexes and ensures a sulfur-rich environment, more closely resembling the one in the native enzyme. Additionally, complexes bearing those S,N-bidentate ligands allowed for important insight into the nature of the W–C_2_H_2_ chemistry. For instance, [WO(C_2_H_2_)(SPhoz)_2_] is capable of reversible binding of C_2_H_2_ with the release of acetylene being triggered by irradiation.^21^ In addition, coordination and subsequent insertion of a second molecule of C_2_H_2_ into the tungsten–nitrogen bond take place in [W(CO)(C_2_H_2_)(PyS)_2_] showing that the alkyne is activated toward the reaction with nucleophiles. The acetylene insertion was studied using C_2_D_2_ and revealed coordination of the second acetylene before insertion.^24^ This represents the first example of a nucleophilic attack on a W-coordinated C_2_H_2_. Similar behavior has previously been observed for tungsten complexes but only with substituted alkynes.^27−34^ To sterically prevent the insertion from taking place, and to favor the attack from an external nucleophile, we introduced a methyl group in position 6 of the PyS ligand. The resulting complex [W(CO)(C_2_H_2_)(6-MePyS)_2_] also reacts with the second molecule of acetylene, but insertion occurs only partially. Moreover, nucleophilic attack of PMe_3_ on coordinated acetylene was observed for W(II) and W(IV) complexes bearing 6-MePyS ligands leading to carbyne and vinyl complexes, respectively.^26^

**Figure 1:**
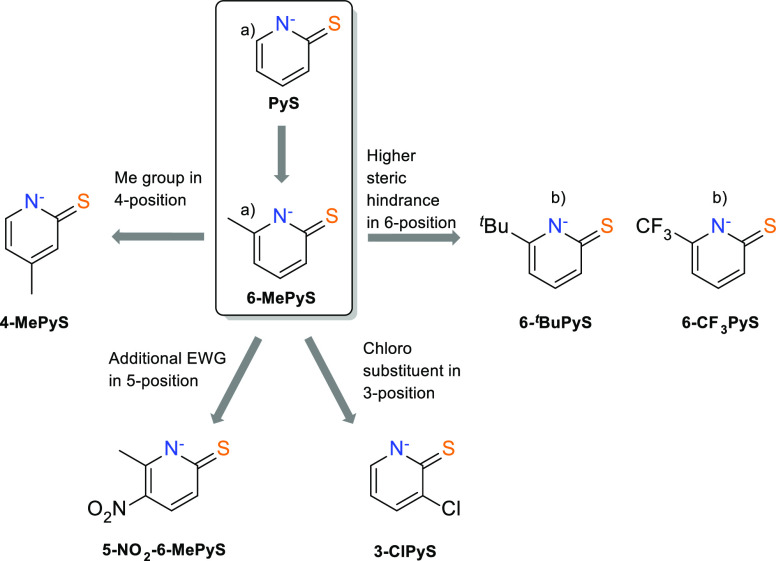
Pyridine-2-thiolates employed for the preparation of W complexes: (a) previously explored in W acetylene chemistry;^24,26^ (b) no coordination to W observed.

Herein, we explore the influence of different pyridine-2-thiolate ligands, namely, 4-methylpyridine-2-thiolate (4-MePyS), 3-chloropyridine-2-thiolate (3-ClPyS), and 5-nitro-6-methylpyridine-2-thiolate (5-NO_2_-6-MePyS) (Figure 1), on the oxidation and acetylene insertion reactivity.

## Results and Discussion

### Choice of Ligands

Ligand design is based on PyS and 6-MePyS which previously allowed the preparation of tungsten acetylene complexes of the types [W(CO)(C_2_H_2_)(SN)_2_] and [WO(C_2_H_2_)(SN)_2_], (SN= bidentate pyridine-2-thiolate moiety).^24,26^ To investigate the insertion reaction, various substituents at the pyridine heterocycle were introduced as displayed in Figure 1. Since the electronic effects of substituents in positions 2, 4, or 6 in pyridine are known to be similar,^35^ we chose 4-MePyS for comparison with 6-MePyS to elucidate whether the insertion is prevented for electronic or steric reasons.^36,37^ The introduction of a nitro group in position 5 in 6-MePyS increases the electron-withdrawing properties, rendering the nitrogen donor less nucleophilic. A similar approach was used by introducing a chloro substituent at the PyS moiety. With both ligands, a reduced reactivity toward insertion is expected. The initial attempts to introduce the Cl to position 6 of the PyS moiety were not successful, so 3-ClPyS was prepared instead. Moreover, coordination of the known ligands 6-*tert*-butylpyridine-2-thiolate (6-^*t*^BuPyS)^38^ and 6-trifluoromethylpyridine-2-thiolate (6-CF_3_PyS) to the tungsten(II) precursor [WBr_2_(CO)_3_(MeCN)_2_] was attempted, but it turned out to be unsuccessful due to steric hindrance of the large groups in position 6.

### Introduction of the Ligand

**Scheme 1:**
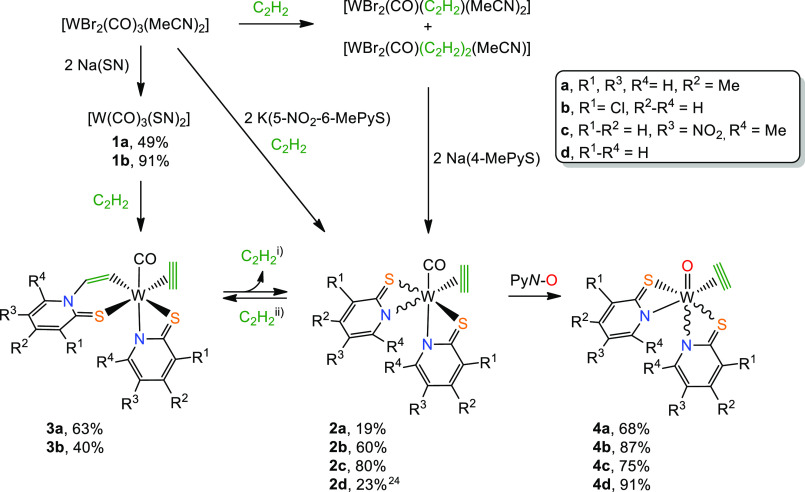
Synthetic Procedures for Complexes **1a**–**b**, **2a**–**c**, **3a**–**b**, and **4a**–**c** For all the reactions with C_2_H_2_, an excess was used (1 atm). (i) Only complex **2b** can be obtained in moderate yield from **3b** via the release of the inserted acetylene (η^1^-C_2_H_2_); (ii) with complex **2c**, no acetylene insertion occurs.

Following the synthetic procedure for complexes employing the ligands PyS and 6-MePyS,^24,26^ the reaction of the tungsten precursor [WBr_2_(CO)_3_(MeCN)_2_] with 2.1–2.2 equiv of each ligand gives the tricarbonyl complex of the general formula [W(CO)_3_(SN)_2_] (Scheme 1). After filtration, complexes [W(CO)_3_(4-MePyS)_2_] (**1a**) and [W(CO)_3_(3-ClPyS)_2_] (**1b**) were isolated, crystallized, and characterized (see Supporting Information (SI)). In contrast, isolation and characterization of the tricarbonyl complex bearing two 5-NO_2_-6-MePyS ligands were not possible due to decomposition under the experimental conditions. ^1^H NMR spectra of **1a** and **1b** show the presence of only one ligand set due to fluxionality of the tricarbonyl moiety at room temperature, which is in accordance with the literature.^39^ Also, IR values related to CO stretching (2011, 1881 cm^–1^ for **1a**; 2014, 1932, 1906 cm^–1^ for **1b**) are in the same range as those known for W(II) tricarbonyl complexes.^40^

However, on the way to tungsten oxo acetylene complexes, the isolation and purification of tricarbonyl compounds turned out to be an unnecessary step. Indeed, in most of the cases, we chose an in situ approach in which the initial reaction of [WBr_2_(CO)_3_(MeCN)_2_] with the ligand salt is immediately followed by the treatment with acetylene.

### Reaction with Acetylene

The reaction solutions of tricarbonyl complexes were purged with acetylene and subsequently stirred, after which the products were isolated as described in the SI. Exposing a toluene solution of **1a** to an acetylene atmosphere (1 atm) leads to the formation of [W(CO)(C_2_H_2_)(4-MePyS)(CHCH-4-MePyS)] (**3a**) where one molecule of acetylene has inserted into the W–N bond and a second C_2_H_2_ is coordinated. This is similar to what has recently been found for [W(CO)_3_(PyS)_2_],^38^ while only partial insertion occurred when position 6 was blocked by a methyl group as with 6-MePyS.^26^ The electronic effects of substituents in positions 2, 4, or 6 in pyridine are known to be similar^35^ indicating that the methyl group in 6-MePyS prevents the insertion for steric reasons only. Although the p*K*_a_ values of the pyridine heterocycles indicate that 4-MePy (6.0) with an additional methyl group is a stronger base than the parent Py (5.2),^37^ complexes bearing their respective thiolate ligand form show the same reactivity toward the insertion. Accordingly, we expected **1b** containing the 3-ClPyS ligand to react to the inserted product [W(CO)(C_2_H_2_)(3-ClPyS)(CHCH-3-ClPyS)] (**3b**) upon exposure to C_2_H_2_. However, after full consumption of the starting material, we consistently observe a mixture of [W(CO)(C_2_H_2_)(3-ClPyS)_2_] (**2b**) and **3b** in a 2:3 ratio. Nevertheless, after 24 h of stirring under acetylene atmosphere, **3b** could be isolated after purification by filtration through silica gel and recrystallization from dichloromethane/heptane in 40% yield as a purple solid. Interestingly, we noticed that stirred solutions of **3b** in dichloromethane gradually change the color from purple to brown. Spectroscopic characterization of this brown mixture (SI) revealed the partial formation of [W(CO)(C_2_H_2_)(3-ClPyS)_2_] (**2b**) due to the release of the inserted acetylene. Full conversion to **2b** was possible by stirring a dichloromethane solution of **3b** under reflux for 6 h. Thus, the most convenient procedure to prepare **2b** was performed by isolating the initial reaction mixture of **2b** and **3b**, subsequent purification by filtration through silica gel, and stirring under reflux for 6 h, which allowed the isolation of **2b** as a brown powder in 60% yield. The release of C_2_H_2_ was also observed with [W(CO)(C_2_H_2_)(PyS)(CHCH-PyS)] (**3a**)^24^ and [W(CO)(C_2_H_2_)(6-MePyS)(CHCH-6-MePyS)],^26^ but only partially and accompanied by the formation of polyacetylene. Thus, the use of a sterically demanding, electron-deficient ligand should prevent insertion reactions altogether. Indeed, the reaction of [WBr_2_(CO)_3_(MeCN)_2_] with 2.1 equiv of K(5-NO_2_-6-MePyS) for 45 min and subsequent flushing with acetylene for another 45 min gave [W(CO)(C_2_H_2_)(5-NO_2_-6-MePyS)_2_] (**2c**) after workup as a brown powder in 80% yield. In contrast to all other investigated pyridine-2-thiolate systems, even after stirring a solution of **2c** in CH_2_Cl_2_ under acetylene atmosphere for 24 h, no inserted complex was detected. We ascribe this to the synergistic effect of the steric demand of the methyl group in the 6-position and the electron-withdrawing properties of the nitro-group. The prevention of insertion is relevant for the oxidation to the biological oxidation state +IV, as all our attempts to oxidize inserted products were futile.

For the synthesis of [W(CO)(C_2_H_2_)(4-MePyS)_2_] (**2a**), we had to apply another synthetic procedure because of the aforementioned favored insertion in complexes with 4-MePyS. Similar to the preparation of [W(CO)(C_2_H_2_)(PyS)_2_],^24^ a metal precursor composed of a mixture of [WBr_2_(CO)(C_2_H_2_)_2_(MeCN)] and [WBr_2_(CO)(C_2_H_2_)(MeCN)_2_] was reacted with Na(4-MePyS) in dichloromethane for 2 h. Complex **2a** was obtained after purification using silica gel and recrystallization from dichloromethane/heptane as dark green crystals in a 19% yield.

### Oxidation to Tungsten(IV) Complexes

Compounds **2a**–**d** can be oxidized to complexes of the type [WO(C_2_H_2_)(SN)_2_] (**4a**–**d**) by a slight excess of pyridine-*N*-oxide (Py*N*O) as an oxygen source in dichloromethane (Scheme 1). The required reaction time for full conversion is highly dependent on the pyridine-2-thiolate ligand and ranges from 10 min in the case of the nitro-substituted **2c** to several hours starting from **2a**, **2b**, and **2d**. All oxido acetylene compounds can be isolated in high yields (**4a** 68%, **4b** 87%, **4c** 75%, and **4d** 91%) as yellow powders. Furthermore, complex **4b** can also be obtained directly from the reaction of Py*N*O with **3b** due to the observed reversibility of the insertion in the latter. Compound **2c** bearing 5-NO_2_-6-MePyS is, in comparison to the other W(II) systems, oxidized significantly faster. This can be explained by a decrease in π-back-donation of tungsten to CO weakening the tungsten carbonyl bond, thereby facilitating the release of CO and oxidation of the metal center. This is supported by IR and X-ray data upon comparison of complex **2c** (ν (CO) 1919 cm^–1^, W–CO: 1.969 Å) with its analogue lacking the nitro group [W(CO)(C_2_H_2_)(6-MePyS)_2_] (ν (CO) 1891 cm^–1^, W–CO: 1.958 Å).^26^

### Spectroscopic Data

Coordination of acetylene was confirmed by ^1^H and ^13^C NMR spectroscopy. In the case of type **2** complexes, acetylenic protons resonate as two singlets in the region 12–14 ppm due to the asymmetry of coordination (Table 1). Complexes **2a** and **2b** are found as a mixture of two isomers in solution, while complex **2c** appears in isomerically pure form. For type **3** complexes, ^1^H NMR spectra show the presence of side-on coordinated (η^2^-C_2_H_2_) and an inserted acetylene (η^1^-C_2_H_2_). Side-on coordinated acetylene resonates in the same region as those in type **2**, while the η^1^-C_2_H_2_ resonates in the form of two doublets in the aromatic region. Both inserted complexes **3a** and **3b** are isomerically pure. Due to C_2_H_2_ release from **3b** and its low solubility, it was not possible to record a meaningful ^13^C NMR spectrum.

**Table 1:** Spectroscopic Data for Tungsten Acetylene Complexes

compound	^1^H NMR of η^2^-C_2_H_2_ [ppm]^a^	^13^C NMR of η^2^-C_2_H_2_ [ppm]^a^	IR (CO) [cm^–1^]	IR (W=O) [cm^–1^]
**2a**	13.62, 12.31	207.2, 206.4	1907	
**2b**	13.80, 12.49	209.4, 207.9	1896	
**2c**	14.10, 12.83	209.8, 207.4	1919	
**3a**	12.91, 11.99	198.4, 193.1	1907	
**3b**	12.96, 12.01	Not available	1903	
**4a**	10.95, 10.94	158.1, 154.7		945
**4b**	11.08, 11.00	157.1, 154.8		936
**4c**	11.44, 11.15	160.2, 158.2		937
**4d**	10.99	158.0, 155.0		937

a In CD_2_Cl_2_; data of major isomer.

In the case of tungsten(IV) oxo species (type **4**), acetylenic proton resonances are upfield-shifted compared to their W(II) analogues and flanked by ^183^W satellites. For complexes **4a**–**c**, the acetylenic protons appear in the form of two singlets. Differently, complex **4d** shows only one singlet for both C_2_H_2_ protons, presumably due to the dynamic behavior of coordinated acetylene. All type **4** complexes, except **4c**, show an additional set of signals related to the presence of a second isomer in solution. As expected, signals are downfield-shifted when 5-NO_2_-6-MePyS is bound to the tungsten center. Even though most of the complexes exhibit two isomers in solution, only one could be crystallized (vide infra). Upon dissolving single crystals, the same ratio of the two isomers is observed pointing toward an equilibrium in solution.

The reactivity and synthetic approaches for obtaining complexes **2**, **3,** and **4** vary significantly depending on the ligand, which is however hardly reflected when comparing NMR and IR data of the respective complexes. Thus, the acetylenic proton shifts in the tungsten(II) acetylene complexes **2a**–**c** are very similar. This can possibly be ascribed to the long distance from the ligand substituents to the acetylenic protons. It suggests that the reactivity differences are primarily influenced by steric effects.

### Molecular Structures

**Figure 2:**
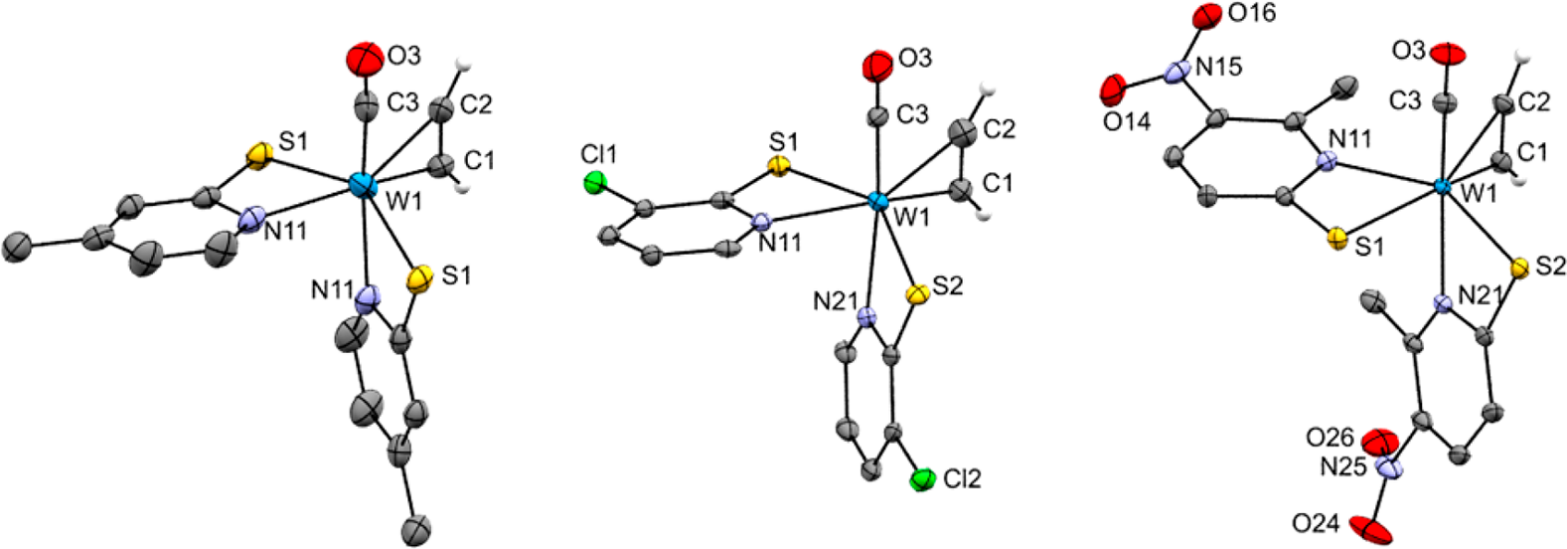
Molecular structures (50% probability thermal ellipsoids) of complexes **2a**–**c** (left to right) showing the atomic numbering scheme. Non-acetylenic H atoms are omitted for clarity reasons.

**Figure 3:**
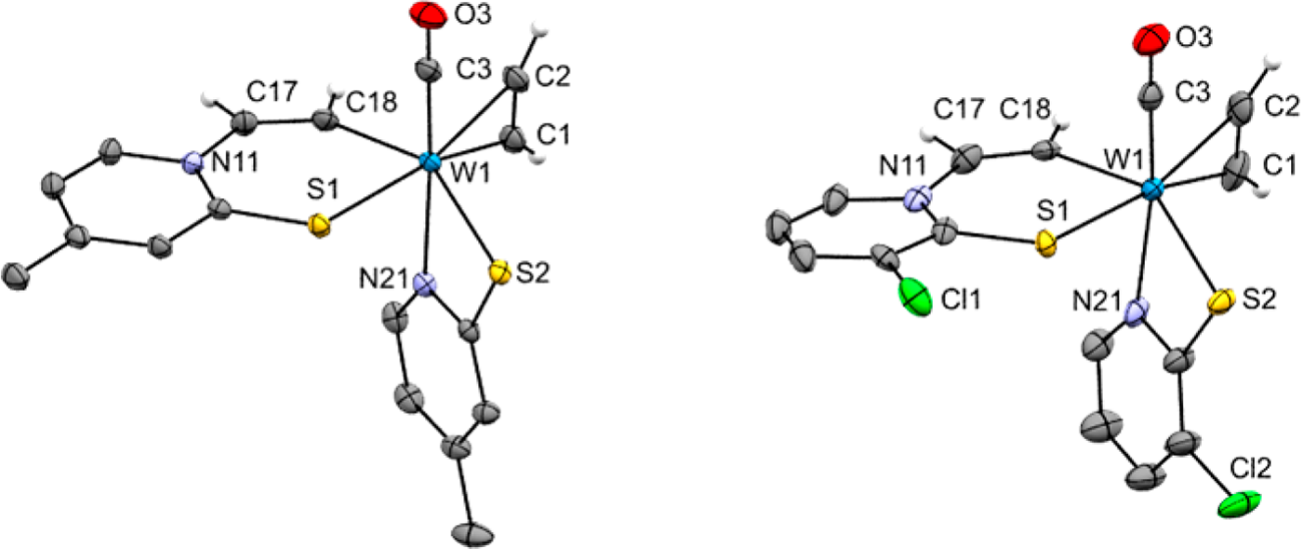
Molecular structures (50% probability thermal ellipsoids) of complexes **3a** (left) and **3b** (right) showing the atomic numbering scheme. Non-acetylenic H atoms are omitted for clarity reasons.

**Figure 4:**
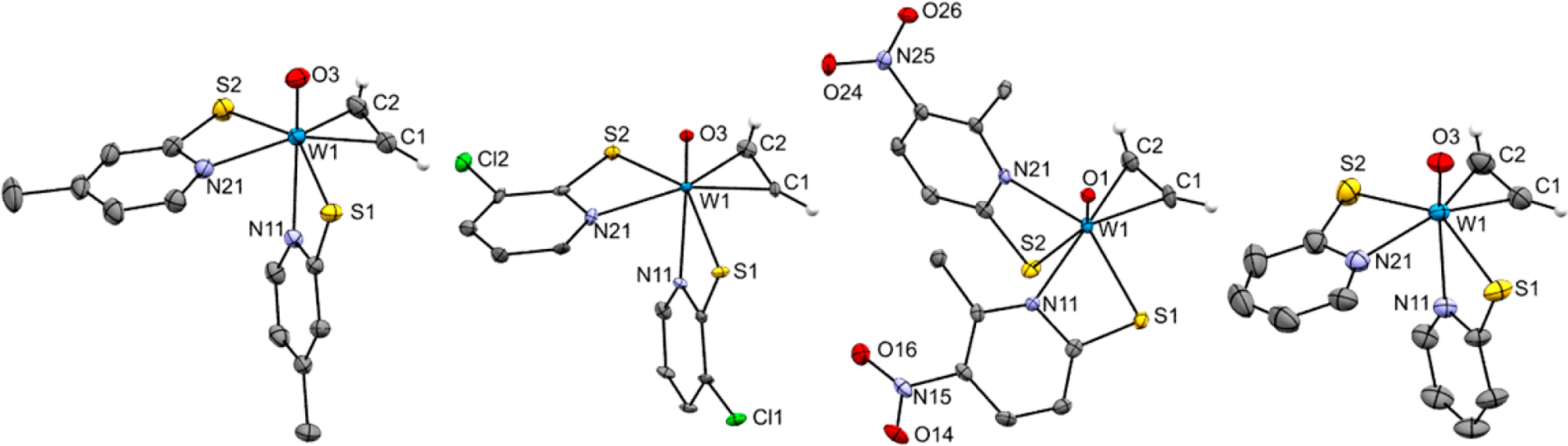
Molecular structures (50% probability thermal ellipsoids) of complexes **4a**–**d** (from left to right) showing the atomic numbering scheme. Non-acetylenic H atoms are omitted for clarity reasons.

The crystal structures of complexes **1a**–**b**, **2a**–**c**, **3a**–**b**, and **4a**–**d** were determined by single-crystal X-ray diffraction analysis. Molecular views of types **2**, **3**, and **4** are given in Figures 2–4. Selected bond lengths of complexes **1**–**4** are presented in Table 2. Full crystallographic details such as structure refinement data as well as experimental details are provided within the SI. All compounds feature distorted octahedral environments around the W atom. The center of the η^2^ C≡C bond occupies the sixth position, which is always located cis to the carbonyl or oxo groups. A significant loss of triple bond character and linearity is observed in all η^2^-bound acetylene molecules, which shows that the actual bonding situation is between the η^2^-adduct and metallacyclopropene resonance structures.

**Table 2:** Selected Bond Lengths for Tungsten Acetylene Complexes

Complex	C≡C^a^	W–C_2_H_2_	WCH=CH	W–CHCHN	C≡O^a^	W–CO	W=O	ref
**2a**	1.302(12)	2.090(13)			1.142(16)	1.884(16)		
		2.078(7)						
**2b**	1.286(6)	2.020(4)			1.148(5)	1.962(4)		
		2.048(5)						
**2c**	1.313(3)	2.023(2)			1.151(3)	1.969(2)		
		2.044(2)						
[W(CO)(C_2_H_2_)(PyS)_2_]	1.316(3)	2.022(2)			1.159(3)	1.973(2)		(24)
		2.045(2)						
[W(CO)(C_2_H_2_)(6-MePyS)_2_]	1.306(7)	2.022(5)			1.167(6)	1.958(5)		(26)
		2.055(3)						
**3a**	1.308(3)	2.031(2)	1.331(3)	2.104(2)	1.157(3)	1.987(2)		
		2.0505(19)						
**3b**	1.314(10)	2.048(5)	1.311(8)	2.101(6)	1.181(9)	1.963(8)		
		2.071(5)						
[W(CO)(C_2_H_2_)(CHCH-PyS)(PyS)]	1.3148(19)	2.0353(13)	1.3421(18)	2.0964(13)	1.161(15)	1.9781(12)		(24)
		2.0582(13)						
[W(CO)(C_2_H_2_)(CHCH-6-MePys)(6-MePyS)]	1.310(3)	2.047(2)	1.349(3)	2.103(2)	1.168(3)	1.974(2)		(26)
		2.065(2)						
[W(CO)(C_2_H_2_)(CHCH-PnS)(PnS)]	1.315(7)	2.036(4)	1.358(6)	2.079(4)	1.156(6)	1.993(5)		(24)
		2.060(4)						
**4a**	1.280(3)	2.095(2)					1.7204(17)	
		2.093(2)						
**4b**	1.274(5)	2.083(3)					1.701(3)	
		2.094(4)						
**4c**	1.258(5)	2.068(3)					1.710(2)	
		2.084(3)						
**4d**	1.260(4)	2.073(2)					1.712(2)	
		2.087(3)						
[WO(C_2_H_2_)(6-MePyS)_2_]	1.279(2)	2.0693(15)					1.7153(13)	(26)
		2.1027(15)						

a Bond lengths are given in Å. Bond lengths of the unbound gases: C_2_H_2_, C**≡**C: 1.186(4) Å;^41^ C**≡**O, 1.12822(7) Å.^42^

Monoacetylene carbonyl complexes (**2a**–**c**) show similar structural properties in terms of bond lengths and angles. Compounds **2a** and **2b** crystallized in S,S-trans configuration, while **2c** crystallized as a S,S-cis isomer, as reported for similar compounds. The carbonyl ligand in **2a**–**c** is trans to the N atoms of the pyridine-2-thiolate ligand. Moreover, the CO shows the common parallel arrangement with coordinated C_2_H_2_.^15^ In complex **2a**, the carbonyl ligand and the acetylene ligand are disordered over two orientations. The complex lies on a twofold rotation axis parallel to the *c*-axis. Moreover, in the case of **2c**, the η^2^-acetylene ligand is trans to the rather long-distant atom S1 (W1–S1 2.5872(6) Å vs W1–S2 2.4024(6) Å). The planes of the nitro groups enclose angles of 28.1(3)° and 29.2(3)° with the pyridine rings they are bonded to.

In both **3a** and **3b**, the W atom is octahedrally surrounded with the two S atoms in cis positions (**3a**: S1–W1–S2 74.995(18)°, **3b**: S1–W1–S2 74.62(7)°), and the η^2^-acetylene ligand as well as the η^1^-C_2_H_2_ group are trans to them. The carbonyl ligands are trans oriented to the N atom of the pyridine-2-thiolate ligand (**3a**: C3–W1–N21 164.99(8)°, **3b**: C3–W1–N21 163.1(3)°) and eclipsed to the acetylene ligand.

Interestingly, the C–C bond lengths of the inserted C_2_H_2_ molecule correlate with the reactivity toward acetylene release. Indeed, complex [W(CO)(C_2_H_2_)(CHCH-PnS)(PnS)], (PnS = 6-(*tert*-butyl)pyridazine-3-thiolate), has the longest inserted C–C bond, being the only one not prone to acetylene release.^24^ On the contrary, for all the other complexes with shorter C–C bonds (Table 2), at least a partial release of inserted acetylene was observed.

Complexes **4a**, **4b**, and **4d** crystallized as S,S-trans isomers. The W–N distances trans to the oxo ligand are significantly longer (**4a**: W1–N11 2.3464(18) Å, **4b**: 2.306(4) Å, **4d**: 2.314(2) Å) than W–N distances trans to the η^2^-acetylene ligand (**4a**: W1–N21 2.219(2) Å, **4b**: 2.242(4) Å, **4d**: 2.221(2)Å). Moreover, the η^2^-C_2_H_2_ shows the typical orthogonal arrangement with the oxo ligand.^16^

Similar to [WO(C_2_H_2_)(6-MePyS)_2_], complex **4c** crystallizes as an S,S-cis isomer. The thiolate group opposite the oxo ligand has a distinctly larger distance to W (W1–S2 2.6283(8) Å) than the other one (W1–S1 2.4153(8) Å). The W–N distance of the ligand opposite the η^2^-acetylene (W1–N11 2.279(3) Å) is also distinctly longer than the other one (W1–N21 2.225(2) Å). The η^2^-acetylene ligand (C1–C2 1.258(5) Å, W1–C_2_ 1.978(3) Å) is almost normal to the W=O bond (C1–C2–W1–O1 88.2(2)°, C2–C1–W1–O1–100.5(2)°) and eclipsed to W1–N21 (C1–C2–W1–N21–177.9(2)°, C2–C1–W1–N21 2.3(2)°).

Steric hindrance in position 6 leads exclusively to the formation of the S,S-cis isomer. The configuration in which both sulfur atoms are trans to each other is more likely to occur within complexes without steric interference in position 6, except for [W(CO)(C_2_H_2_)(PyS)_2_] which crystallized as S,S-cis isomer. This trend is also reflected in the tricarbonyl complexes as presented in Table 3 (for crystallographic details, see SI Figures S1–2, Tables S1,S6–7). Moreover, all inserted complexes exhibit exclusively the S,S-cis configuration.

**Table 3:** Orientation of the Ligands within W Complexes Based on Molecular Structures

	PyS	6-MePyS	4-MePyS	3-ClPyS	5-NO_2_-6-MePyS
[W(CO)_3_(SN)_2_]	S,S-trans	S,S-cis	S,S-trans	S,S-trans	
[W(CO)(C_2_H_2_)(SN)_2_]	S,S-cis	S,S-cis	S,S-trans	S,S-trans	S,S-cis
[W(CO)(C_2_H_2_)(CHCH-SN)(SN)]	S,S-cis	S,S-cis	S,S-cis	S,S-cis	
[WO(C_2_H_2_)(SN)_2_]	S,S-trans	S,S-cis	S,S-trans	S,S-trans	S,S-cis

The geometry of type **2** complexes is likely influencing the reactivity toward acetylene, especially since the second molecule of C_2_H_2_ seems to coordinate to the W(II) center prior to insertion.^24^ The latter occurs more easily when the metal center is not shielded by methyl groups in position 6. Moreover, carbonyl acetylene complexes bearing 6-MePyS^26^ and 5-NO_2_-6-MePyS (**2c**) ligands exist as a single isomer in solution (S,S-cis), and this orientation may be too rigid to undergo the coplanar rearrangement necessary for the migratory insertion. In contrast, other pyridine-2-thiolate based complexes with no substituent at position 6 show the presence of two isomers in solution. This suggests that for the insertion, the S,S-trans configuration is required. Flexible isomerization between the two configurations might facilitate the reactivity toward the coordination of the second molecule of acetylene.

## Conclusion

Here, we report the influence of different substitutional patterns on the reactivity of W(II) complexes toward the acetylene insertion and oxidation process. Comparison of the electronically similar 4-MePyS with 6-MePyS systems allows the conclusion that steric factors govern the reactivity toward insertion. The presence of the methyl group in the 6-MePyS complex prevents insertion of acetylene, while with 4-MePyS, the inserted compound [W(CO)(C_2_H_2_)(CHCH-4-MePyS)(4-MePyS)] was obtained. No insertion is observed by combining the steric effect of the methyl group and the electronic effect of the nitro group in 5-NO_2_-6-MePyS. The predominance of steric effects is also demonstrated by the 3-ClPyS system where the presence of an electron-withdrawing group in position 3 does not prevent insertion. However, the inserted complex is significantly more sensitive toward releasing C_2_H_2_ in solution compared to **3a**, which exhibits a methyl group in position 4. Moreover, the tendency to release inserted acetylene from type **3** complexes decreases with the C–C bond lengths of the η^1^-C_2_H_2_ moiety. Tungsten(IV) oxo acetylene complexes [WO(C_2_H_2_)(SN)_2_] can be synthesized from W(II) acetylene species with pyridine-*N*-oxide. When using 5-NO_2_-6-MePyS, π-back-donation from tungsten to the carbonyl in type **2** is reduced, thereby weakening the tungsten carbonyl bond and thus leading to a shorter oxidation reaction time. Complexes with pyridine-2-thiolate ligands substituted in position 6 are prone to adopt the S,S-cis orientation, while the sterically less demanding systems tend to crystallize in the S,S-trans configuration. We assume that the flexibility of the ligand coordination in W(II) complexes with 4-MePyS and 3-ClPyS ligands allows further reactivity with a second molecule of acetylene required for insertion. To our surprise, regardless of the substitutional patterns of pyridine-2-thiolate ligands, all the complexes containing η^2^-C_2_H_2_ have similar structural properties within their groups.

## Supporting Information Available

Full X-ray data, NMR spectra, and experimental procedures (PDF)

## Accession Codes

CCDC 2103131–2103141 contain the supplementary crystallographic data for this paper. These data can be obtained free of charge via www.ccdc.cam.ac.uk/data_request/cif, or by emailing data_request@ccdc.cam.ac.uk, or by contacting The Cambridge Crystallographic Data Centre, 12 Union Road, Cambridge CB2 1EZ, UK; fax: +44 1223 336033.
