# Supplementary material for: The Effect of Pyridine-2-thiolate Ligands on the Reactivity of Tungsten Complexes toward Oxidation and Acetylene Insertion
Source: Organometallics. 2021 Oct 18;40(21):3591–8. doi: 10.1021/acs.organomet.1c00472 (PMC8579403; doi:10.1021/acs.organomet.1c00472)
Supplement: Supplementary file 1 — om1c00472_si_001.pdf [file om1c00472_si_001.pdf]

Supporting Information

The Effect of Pyridine-2-thiolate Ligands on the Reactivity of Tungsten Complexes Toward Oxidation and Acetylene Insertion

Riccardo Bondi,[‡,a] *Miljan Z. Ćorović,*[‡,a] *Michael Buchsteiner,*[a,1] *Carina Vidovič,*[a,2] *Ferdinand Belaj,*[a] *Nadia C. Mösch-Zanetti**[a]

[a]*Institute of Chemistry, Inorganic Chemistry, University of Graz, 8010 Graz, Austria*

‡These authors contributed equally.

* Corresponding author. Tel.: +43 (0)316 380 – 5286

E-mail address: nadia.moesch@uni-graz.at

Table of Contents

1 General Considerations S1

2 Syntheses S2

3 Crystal Structure Determination S13

4 NMR spectra S31

5 References S53

1 General Considerations

All experiments were carried out under inert atmosphere employing standard Schlenk and glovebox techniques unless otherwise stated. All chemicals were purchased from commercial sources and except for acetylene, and pyridine-N-oxide all were used without further purification. Acetylene 2.6 was washed with water and conc. H_2SO_4 and dried with $CaCl_2$ and KOH. Pyridine-N-oxide was purified by sublimation. All solvents were purified by a Pure Solv Solvent Purification System and stored over activated molecular sieves (3 Å). Silica gel used for purification was previously washed with Et_3N and dried *in vacuo*.

NMR spectra were recorded using a Bruker Avance III spectrometer. ^1H NMR spectra were recorded at 300.13 MHz and referenced to residual protons of the NMR solvents. ^{13}C NMR spectra were obtained at 75.48 MHz and spectra were referenced to the deuterated solvent peak. The chemical shifts δ are given in ppm. The multiplicity of peaks is denoted as broad singlet (bs), singlet (s), doublet (d), triplet (t), quadruplet (q), multiplet (m), and doublet of quadruplet (dq). Coupling constants J are given in Hertz. Mass spectroscopy measurements using electron impact ionization (EI-MS) have been performed with an Agilent 5973 MSD with a push rod for direct sample measurement. IR spectra were recorded in the solid-state at a resolution of 2 cm^{-1} on a Bruker ALPHA-P Diamant ATR-FTIR. Elemental analyses (C, H, N, S) were carried out by the Microanalytical Laboratory, Department of Chemistry, University of Vienna, and the Department of Inorganic Chemistry at the Graz University of Technology of (Heraeus Vario Elementar automatic analyzer). $[WBr_2(CO)_3(MeCN)_2]$[1], $[WBr_2(CO)(C_2H_2)(MeCN)_2]$/ $[WBr_2(CO)(C_2H_2)_2(MeCN)]$[2] mixture and $[W(CO)(C_2H_2)(PyS)_2]$[2] were prepared according to literature. 4-MePySH, 3-ClPySH, and 5-NO_2-6-MePySH were prepared according to modified literature procedures.[3,4] 6-(Trifluoromethyl)pyridine-2(*1H*)-thione was purchased from Apollo Scientific, and deprotonated with pure NaH in THF, while Na(6-^{t}BuPyS) and Na(4-MePyS) were synthesized according to the literature procedures.[5]

2 Syntheses

Synthesis of ligands and ligand precursors

3-Chloropyridine-2(*1H*)-thione (3-ClPySH). 3-Chloropyridine-2(*1H*)-thione was synthesized using a modified literature procedure.[3] A 250 mL Schlenk flask was charged with 2,3-dichloropyridine (2.98 g, 20.1 mmol, 1 equiv), thiourea (1.84 g, 24.1 mmol, 1.2 equiv), and anhydrous ethanol (60 mL). The mixture was stirred at reflux for 60 h and, after cooling, the yellow suspension obtained was evaporated *in vacuo* to dryness. The resulting residue was treated with a solution of NaOH (2.40, 60 mmol, 3 equiv) in 50 mL of degassed water. The mixture was then stirred upon reflux for 2 h. After cooling, the white solid formed was removed by filtration and the resulting solution was cooled to 0 °C with an ice bath. The product was then precipitated with the addition of acetic acid, collected by filtration, washed with water and diethyl ether (2 x 5 mL), and dried in air and *vacuo* yielding 2.46 g (84%) of the product as a yellow solid.

^{1}H NMR (300 MHz, DMSO-d_6) δ 14.01 (s, 1H), 7.87 (dd, J = 7.6, 1.6 Hz, 1H), 7.71 (dd, J = 6.2, 1.6 Hz, 1H), 6.75 (dd, J = 7.6, 6.1 Hz, 1H).

Sodium 3-chloropyridine-2-thiolate, Na(3-ClPyS). In a 100 mL Schlenk flask 3-chloropyridine-2(*1H*)-thione (2.46 g, 16.9 mmol, 1 equiv) was suspended in 40 mL of THF. NaH (410 mg, 17.0 mmol, 1.01 equiv) was added portion-wise over 10 min, the suspension was stirred for an additional 1.5 h and the fine solid was removed by filtration over Celite. The Celite layer was subsequently washed with 2 x 10 mL of THF and the solvent was removed *in vacuo.* The obtained residue was washed with heptane (2 x 15 mL), diethyl ether (2 x 10 mL), and dried *in vacuo* yielding 2.47 g (87%) of Na(3-ClPyS) as an off-white powder.

^{1}H NMR (300 MHz, DMSO-d_6) δ 7.77 (dd, J = 4.6, 1.8 Hz, 1H), 7.24 (dd, J = 7.5, 1.8 Hz, 1H), 6.37 (dd, J = 7.5, 4.6 Hz, 1H).

^{13}C NMR (75 MHz, DMSO-d_6) δ 178.2 (C-S), 146.0, 133.9 (C-Cl), 133.6, 112.9.

6-Methyl-5-nitropyridine-2(*1H*)-thione (5-NO_2-6-MePySH). 6-Methyl-5-nitropyridine-2(*1H*)-thione was synthesized using a modified literature procedure.[4] 6-Chloro-2-methyl-3-nitropyridine (10.43 g, 0.060 mol, 1 equiv) and thiourea (5.20 g, 0.068 mol, 1.13 equiv) were placed in a 250 mL flask. After the addition of 200 mL of dry ethanol, the mixture was stirred upon reflux over the night. After the solvent was removed, the product was dissolved in 200 mL of KOH solution (5.08 g in 200 mL H_2O) and refluxed for an hour. The mixture was diluted with a NaOH solution (12.00 g in 100 mL H_2O) and filtrated over a sintered funnel with filter paper. The collected solution was cooled to rt and washed three times with 100 mL CH_2Cl_2. The water layer was then acidified with concentrated HCl to pH 4, and the temperature was regulated with an ice bath to be around rt. After filtration, the yellow powder was washed with cold ethanol and dried, yielding 8.025 g (78%).

^{1}H NMR (300 MHz, DMSO-d_6) δ 13.95 (s, 1H), 7.97 (d, J = 9.5 Hz, 1H), 7.18 (d, J = 9.5 Hz, 1H), 2.72 (s, 3H).

Potassium 6-methyl-5-nitropyridine-2-thiolate, K(5-NO_2-6-MePyS). 6-Methyl-5-nitropyridine-2-thiol (5.43 g, 31.9 mmol, 1 equiv) was dissolved in 60 mL of dry THF in a 100 mL Schlenk flask. The solution was sonicated and cooled to 0˚C. KH (1.42 g, 31.8 mmol, 0.99 equiv, 90% in oil) was added in portions. The mixture was stirred for 10 mins at 0˚C, and 1h at rt. Hydrogen evolution was apparent. The solution was then concentrated to 10 mL and overlaid with 100 mL of diethyl ether. The filtrate was cannulated off and the red powder washed twice with 20 mL of diethyl ether. Subsequent drying *in vacuo* yielded 6.48 g (97 %) of the product.

^{1}H NMR (300 MHz, DMSO-d_6) δ 7.55 (d, J = 8.9 Hz, 1H), 6.86 (d, J = 8.9 Hz, 1H), 2.55 (s, 3H).

Synthesis of [W(CO)$_3$(SN)$_2$], type 1

[W(CO)$_3$(4-MePyS)$_2$] (1a). [WBr$_2$(CO)$_3$(MeCN)$_2$] (0.209 g, 0.39 mmol, 1.0 equiv) and Na(4-MePyS) (0.133 g, 0.87 mmol, 2.2 equiv) were placed into a 50 mL Schlenk flask and suspended in 20 mL of CH_2Cl_2. A bubbler was placed onto the flask and the suspension was stirred for 1 h, whereby the color changed from brownish to orange. Solids were removed via cannula filtration. The filtrate was overlaid with 30 mL of heptane and the brown impurity was formed. The impurity was filtered off over a plug of Celite and after filtration, approx. 5 mL of solvent were removed and the orange solution was placed into the -20 °C freezer. After 3 days orange-red crystals (0.098 g, 49%) were isolated by cannula filtration.

^{1}H NMR (300 MHz, CD_2Cl_2) δ = 8.27 (d, J = 5.7, 2H), 6.72 (m, 2H), 6.66 (m, 2H), 2.21 (s, 6H, CH_3).

^{13}C NMR (75 MHz, CD_2Cl_2) δ = 235.7 ($J_{W\text{-}C}$ =119.0 Hz, CO), 176.5 (C-S), 150.7 (C-CH_3), 145.0, 128.3, 120.8, 21.7 (CH_3).

IR (cm^{-1}): ν (C≡O): 2011, 1881

Anal. Calcd for $C_{15}H_{12}N_2O_3S_2W$: C, 34.90; H, 2.34; N, 5.43; S, 12.42. Found: C, 35.22; H, 2.43; N, 5.50; S, 12.07.

[W(CO)$_3$(3-ClPyS)$_2$] (1b). [WBr$_2$(CO)$_3$(MeCN)$_2$] (551 mg, 1.08 mmol, 1 equiv) and Na(3-ClPyS) (387 mg, 2.31 mmol, 2.13 equiv) were placed into a 100 mL Schlenk flask and suspended in 30 mL of CH_2Cl_2. The flask was equipped with a bubbler and the suspension was stirred for 1 h. A dark orange suspension was obtained and solids were removed by filtration over a pad of Celite which was subsequently eluted with CH_2Cl_2 (2 x 5 mL). The solvent was concentrated *in vacuo* to ca. 15 mL and 35 mL of heptane were added while stirring. The solution was carefully concentrated to approx. 20 mL, obtaining an orange precipitate. The solvent mixture was cannulated off and the solid was washed with heptane (2 x 5 mL) and dried *in vacuo* to get 547 mg (91 %) of the product as an orange powder. Crystals suitable for X-ray

diffraction analysis were obtained from a saturated solution in CH_2Cl_2 carefully overlaid with heptane and placed in the freezer at -25°C.

^1H NMR (300 MHz, CD_2Cl_2) δ 8.37 (dd, J = 5.5, 1.5 Hz, 2H), 7.60 (dd, J = 8.1, 1.4 Hz, 2H), 6.88 (dd, J = 8.1, 5.5 Hz, 2H).

^{13}C NMR (75 MHz, CD_2Cl_2) δ 233.3 ($J_{W\text{-}C}$ =119.9 Hz, CO), 174.7 (C-S), 144.4, 137.7, 133.1 (C-Cl), 119.0.

IR (cm^{-1}): ν (C≡O): 2014, 1932, 1906

LRMS (EI-MS, m/z): Calcd for $C_{10}H_6Cl_2N_2S_2W$ [M^+ -3CO] 473.9, found 473.9.

Anal. Calcd for $C_{13}H_6Cl_2N_2O_3S_2W$: C, 28.03; H, 1.09; N, 5.03; S, 11.51. Found: C, 27.86; H, 1,10; N, 4.85; S, 11.47

Synthesis of [W(CO)(C_2H_2)($SN)_2$], type 2, and [W(CO)(C_2H_2)(CHCH−SN)(SN)], type 3.

[W(CO)(C_2H_2)(4-MeSPy$)_2$] (2a). The precursor mixture [WBr_2(CO)(C_2H_2)(MeCN$)_2$]/ [WBr_2(CO)($C_2H_2$$)_2$(MeCN)] (0.504 g) and Na(4-MePyS) (0.350 g, 2.37 mmol) were placed in a 100 mL Schlenk flask, suspended in approx. 100 mL of CH_2Cl_2 and nitrogen was immediately bubbled through the solution with a bubbler. Evaporated CH_2Cl_2 was continuously compensated. After 2 h the bubbler was removed and a spatula of silica gel was added. An olive-green solution was obtained via cannula filtration. The solution was overlaid with 40 mL of heptane and the solvent was carefully removed *in vacuo* until a green powder precipitated. The powder was isolated via cannula filtration and again dissolved in 20 mL CH_2Cl_2. The solution was overlaid with 20 mL of heptane. The brownish residue, which precipitated overnight, was separated via cannula filtration. The filtrate was placed in a -20 °C freezer overnight. Formed green needles were isolated from the solution via cannula filtration to obtain the product in 19% (0.100 g) yield. Single crystals suitable for X-ray diffraction analysis were obtained from a saturated acetonitrile solution at -35 °C.

^{1}H NMR (300 MHz, CD_2Cl_2, major isomer) δ 13.62 (s, 1H, C≡CH), 12.31 (s, 1H, C≡CH), 8.72 (d, *J* = 5.4 Hz, 1H), 8.45 (d, *J* = 5.8 Hz, 1H), 6.93 (m, 1H), 6.71 (m, 1H), 6.65 (dd, *J* = 5.8, 1.1 Hz, 1H), 6.54 (m, 1H), 2.29 (s, 3H, CH_3), 2.18 (s, 3H, CH_3).

^{13}C NMR (75 MHz, CD_2Cl_2, major isomer) δ 241.4 (CO), 207.2 (C_2H_2), 206.4 (C_2H_2), 177.9 (C-S), 171.0 (C-S), 151.8, 151.3, 149.5 (C-CH_3), 148.0 (C-CH_3), 146.4, 145.6, 127.3, 126.8, 121.8, 119.4, 22.0 (CH_3), 21.5 (CH_3)

IR (cm^{-1}): ν (C≡O): 1907

Anal. Calcd for $C_{15}H_{14}N_2OS_2W$: C, 37.05; H, 2.90; N, 5.76; S, 13.19. Found: C, 37.33; H, 3.01; N, 5.62; S, 13.09.

[W(CO)(C_2H_2)(CHCH-4-MePyS)(4-MePyS)] (3a). [W(CO)$_3$(4-MePyS)$_2$] (**1a**) (0.532 g, 1.01 mmol, 1.0 equiv) was placed in a 100 mL Schlenk flask and dissolved in 50 mL of toluene. The suspension was purged with acetylene for 1.4 hours and stirred overnight. The next day, toluene was removed and the residue was dissolved in 20 mL of CH_2Cl_2. The solution was filtered through a plug of silica gel. The silica gel plug was washed with dichloromethane until the intense red-violet color of the filtrate vanished. The filtrate was concentrated to approx. 15 mL *in vacuo* and was overlaid with 30 mL of heptane. Approximately half of the solvent was evaporated slowly to obtain a red-violet precipitate. The precipitate was isolated via cannula filtration and washed with 5 mL of heptane. The obtained product was dried *in vacuo* to give 0.326 g (63%) of a maroon powder. Single crystals suitable for X-ray diffraction analysis were obtained from a saturated solution in dichloromethane carefully overlaid with heptane (dichloromethane: heptane = 1 : 1).

^{1}H NMR (300 MHz, CD_2Cl_2) δ 12.91 (s, 1H, C≡CH), 11.99 (s, 1H, C≡CH), 8.08 (d, *J* = 5.6 Hz, 1H), 7.73 – 7.56 (m, 3H, 2 arom. H + η^1-C_2H_2), 6.77 – 6.70 (m, 2H), 6.66 (m, 1H), 6.50 (d, *J* = 10.9 Hz, 1H, η^1-C_2H_2), 2.28 (s, 3H, CH_3), 2.25 (s, 3H, CH_3).

^{13}C NMR (75 MHz, CD_2Cl_2) δ 232.6 (CO), 198.4 (C_2H_2), 193.1 (C_2H_2), 179.1 (C-S), 166.7 (η^1-C_2H_2), 160.2 (C-S), 148.7 (C-CH_3), 147.6, 145.3 (C-CH_3), 139.7 (η^1-C_2H_2), 133.3, 132.2, 128.1, 120.0, 119.3, 21.7 (s, CH_3), 20.8 (s, CH_3).

IR (cm^{-1}): ν (C≡O): 1907

LRMS (EI-MS, m/z): Calcd for $C_{12}H_{12}N_2S_2W$ [M^+ - CO - $2C_2H_2$] 432.0, found 432.0.

Anal. Calcd for $C_{17}H_{16}N_2OS_2W \cdot 0.1\ CH_2Cl_2$: C, 39.44; H, 3.14; N, 5.38; S, 12.31. Found: C, 39.37; H, 3.12; N, 5.29; S, 12.05.

[W(CO)(C_2H_2)(3-ClPyS)$_2$] (2b). A 100 mL Schlenk flask was charged with [$WBr_2(CO)_3(MeCN)_2$] (497 mg, 0.97 mmol, 1 equiv), Na(3-ClPyS) (365 mg, 2.18 mmol, 2.25 equiv) and CH_2Cl_2 (30mL), equipped with a bubbler, and the suspension was stirred for 1h. The dark orange suspension was flushed with C_2H_2 for 25 min and stirred overnight under C_2H_2 atmosphere obtaining a dark purple suspension. After removing all the volatiles *in vacuo*, the brown solid was suspended in CH_2Cl_2 (30mL) and filtered through a layer of silica gel in a Schlenk frit. The silica gel was washed with ca. 50 mL of CH_2Cl_2 until the intense purple color vanished. Thereafter, the solvent was reduced to ca. 30 mL, and the solution was heated to reflux for 6h. The brownish suspension was filtered through silica gel and the silica layer was washed with 30 mL of CH_2Cl_2. The volume of the filtrate was reduced to ca. 30 mL, whereupon heptane (20 mL) was added. The brown solid obtained after further evaporation of the solvents to ca. 15 mL, was collected through cannula filtration, washed with pentane (5 x 5 mL), and dried *in vacuo* to get 306 mg (0.58 mmol, 60 %) of [W(CO)(C_2H_2)(3-ClPyS)$_2$] as a brown powder. Crystals suitable for X-ray diffraction analysis were obtained from a solution in DCM (2 mL) mixed with benzene (0.5 mL) and carefully overlaid with heptane (2 mL) at -25°C.

^1H NMR (300 MHz, CD_2Cl_2, major isomer) δ 13.80 (s, 1H, C≡CH), 12.49 (s, 1H, C≡CH), 8.81 (dd, *J* = 5.2, 1.5 Hz, 1H), 8.57 (dd, *J* = 5.5, 1.4 Hz, 1H), 7.76 (dd, *J* = 8.2, 1.5 Hz, 1H), 7.48 (dd, *J* = 7.9, 1.4 Hz, 1H), 7.14 – 7.06 (m, 1H), 6.79 (dd, *J* = 7.9, 5.5 Hz, 1H).

^{13}C NMR (75 MHz, CD_2Cl_2, major isomer) δ 238.8 (CO), 209.4 (C_2H_2), 207.9 (C_2H_2), 176.9 (C-S), 169.0 (C-S), 145.6, 144.8, 139.1, 136.9, 132.3 (C-Cl), 131.9 (C-Cl), 120.7, 117.4.

IR (cm^{-1}): ν (C≡O): 1896

LRMS (EI-MS, m/z): Calcd for $C_{13}H_8Cl_2N_2OS_2W$ [M^+] 527.9, found 528.0. Calcd for $C_{12}H_8Cl_2N_2S_2W$ [M^+ - CO] 499.9, found 500.0. Calcd for $C_{10}H_6Cl_2N_2S_2W$ [M^+ - CO - C_2H_2] 473.9, found 474.0.

Anal. Calcd for $C_{13}H_8Cl_2N_2OS_2W$: C, 29,62; H, 1,53; N, 5,31; S, 12,16. Found: C, 29,49; H, 1,55; N, 5,11; S, 12,15.

[W(CO)(C_2H_2)(3-ClPyS)(CHCH-3-ClPyS)] (3b). A 100 mL Schlenk flask was charged with [$WBr_2(CO)_3(MeCN)_2$] (247 mg, 0.48 mmol, 1 equiv), Na(3-ClSPy) (184 mg, 1.10 mmol, 2.3 equiv) and 30 mL of CH_2Cl_2. The flask was equipped with a bubbler and the suspension was stirred for 1 h, obtaining a dark orange suspension. The flask was covered with Alu-foil and acetylene was flushed through the suspension for 30 min. After stirring under acetylene atmosphere for 9 h, the flask was flushed again with acetylene for 30 min and stirred overnight. The purple suspension was filtered through a layer of silica gel in a Schlenk frit while all the glassware was covered with aluminum foil. The purple solution was concentrated to ca 20 mL, carefully overlaid with heptane (ca 20 mL) and the flask was placed in the freezer at -25°C. After 2 days, a purple crystalline solid was isolated through cannula filtration, dried *in vacuo* to get 107 mg (0.19 mmol, 40%) of **3b**. Single crystals suitable for X-ray diffraction analysis were obtained from a solution in CH_2Cl_2 overlaid with heptane at -25°C.

^1H NMR (300 MHz, CD_2Cl_2) δ 12.96 (s, 1H, C≡CH), 12.01 (s, 1H, C≡CH), 8.14 (d, J = 6.7 Hz, 1H), 7.82 (d, J = 10.9 Hz, 1H, η^1-C_2H_2), 7.80 – 7.77 (m, 2H), 7.66 – 7.53 (m, 2H), 6.89 (s, 1H), 6.70 (d, J = 10.9 Hz, 1H, η^1-C_2H_2).

IR (cm^{-1}): ν (C≡O): 1903

LRMS (EI-MS, m/z): Calcd for $C_{14}H_{10}Cl_2N_2S_2W$ [M^+ - CO] 525.9, found 526.0. Calcd for $C_{12}H_8Cl_2N_2S_2W$ [M^+ - CO - C_2H_2] 499.9, found 500.0. Calcd for $C_{10}H_6Cl_2N_2S_2W$ [M^+ - CO - 2 C_2H_2] 473.9, found 473.9.

Anal. Calcd for $C_{15}H_{10}Cl_2N_2OS_2W$: C, 32.57; H, 1.82; N, 5.06; S, 11.59. Found: C, 32.42; H, 1,89; N, 4.88; S, 11.65.

[W(CO)(C_2H_2)(5-NO_2-6-MePyS)$_2$] (2c). [$WBr_2(CO)_3(MeCN)_2$] (1 g, 1.96 mmol, 1 equiv) and K(5-NO_2-6-MePyS) (0.825 g, 3.96 mmol, 2.02 equiv) were suspended in 100 mL Schlenk flask with 50 mL of dry MeCN and stirred for 30 min with a bubbler attached. The flask was afterwards connected to the acetylene line and the reaction mixture was stirred for another 30 min. The solution changed color from red to brown, and the solvent was removed *in vacuo*. The solid residue was suspended in CH_2Cl_2 and filtered over Celite. After two crystallizations from a CH_2Cl_2/heptane mixture (95/5), and cannula filtration of a solvent, dark orange crystals were obtained in 80 % yield. (0.905 g)

^{1}H NMR (300 MHz, CD_2Cl_2) δ 14.10 (s, 1H, C≡CH), 12.83 (s, 1H, C≡CH), 8.30 (d, J = 8.8 Hz, 1H), 7.74 (s, 0H), 6.93 (d, J = 8.8 Hz, 1H), 6.74 (d, J = 8.9 Hz, 1H), 2.40 (s, 3H, CH_3), 1.50 (s, 3H, CH_3).

^{13}C NMR (75 MHz, CD_2Cl_2) δ 231.4 (CO), 209.8 (C_2H_2), 207.4 (C_2H_2), 184.4 (C-S), 177.6 (C-S), 155.1 (C-NO_2), 151.5 (C-NO_2), 143.1 (C-CH_3), 142.9 (C-CH_3), 135.3, 132.2, 125.8, 125.49, 24.3 (CH_3), 22.6 (CH_3).

IR (cm^{-1}): ν (C≡O): 1919

Anal. Calcd. for $C_{15}H_{12}N_4O_5S_2W \cdot 0.5\ CH_2Cl_2$: C, 30.09; H, 2.12; N, 9.06; S, 10.36. Found: C, 30.41; H, 2.10; N, 9.31; S, 10.62.

Synthesis of [WO(C_2H_2)(SN)$_2$], type 4

[WO(C_2H_2)(4-MePyS)$_2$] (4a). [W(CO)(C_2H_2)(4-MeSPy)$_2$] (**2a**) (0.500 g, 1.03 mmol, 1.0 equiv) and pyridine-*N*-oxide (0.108 g, 1.13 mmol, 1.1 equiv) were placed in a 100 mL Schlenk flask and suspended in 20 mL of MeCN. A bubbler was placed onto the Schlenk flask. The suspension was stirred overnight and the color changed from violet-red to dark ocher. The solvent was evaporated completely *in vacuo* and the residue was washed 5 times with 5 mL of diethyl ether to obtain the product as a light brown powder in 68% (0.333 g) yield. Single crystals suitable for X-ray diffraction analysis were obtained from a saturated solution in ethyl acetate carefully overlaid with heptane (ethyl acetate : heptane = 1 : 1) at -35 °C.

^{1}H NMR (300 MHz, CD_2Cl_2, major isomer) 10.95 (s, 1H, C≡CH), 10.94 (s, 1H, C≡CH), 9.02 (d, *J* = 5.4 Hz, 1H), 7.35 (d, *J* = 5.6 Hz, 1H), 6.94 (m, 2H), 6.85 – 6.79 (m, 1H), 6.34 (dd, *J* = 5.7, 0.9 Hz, 1H), 2.30 (s, 3H, CH_3), 2.09 (s, 3H, CH_3).

^{13}C NMR (75 MHz, CD_2Cl_2, major isomer) δ 177.0 (C-S), 168.3 (C-S), 158.1 (s(d), J_{WC} = 36.0 Hz, C_2H_2), 154.7 (s(d), J_{WC} = 26.5 Hz, C_2H_2), 153.8 (C-CH_3), 149.7 (C-CH_3), 143.2, 142.8, 128.0, 127.6, 121.8, 119.3, 22.1 (CH_3), 21.5 (CH_3).

IR (cm^{-1}): ν (W=O): 945

Anal. Calcd for $C_{14}H_{14}N_2OS_2W$ · 0.05 EtOAc: C, 35.63; H, 3.03; N, 5.85; S, 13.40. Found: C, 35.55; H, 2.82; N, 5.98; S, 13.03.

[WO(C_2H_2)(3-ClPyS)$_2$] (4b). A 50 mL Schlenk was charged with [W(CO)(C_2H_2)(3-ClPyS)$_2$], (**2b**), (182 mg, 0.34 mmol, 1 equiv), pyridine-*N*-oxide (38 mg, 0.40 mmol, 1.18 equiv) and CH_2Cl_2 (20 mL). The flask was equipped with a bubbler and the dark green suspension was stirred overnight. The brownish suspension was filtered through Celite and washed with CH_2Cl_2 until the brownish color of the filtrate vanished. The solution was concentrated to approx. 20 ml and 10 mL of MeCN were added. A light brown crystalline solid was obtained upon slow evaporation of the solvent mixture. The product was isolated through cannula filtration, washed

with pentane (3 x 3 mL), and dried *in vacuo* obtaining 152 mg (87%) of [WO(C_2H_2)(3-ClPyS)$_2$] as a light brown crystalline solid. Single crystals suitable for X-ray diffraction analysis were obtained by recrystallization from CH_2Cl_2/MeCN (1/1) at -25°C.

^{1}H NMR (300 MHz, CD_2Cl_2, major isomer): δ 11.08 (s(d), 1H, J_{WH} = 10.4 Hz, C≡CH), 11.00 (s(d), 1H, J_{WH} = 11.0 Hz, C≡CH), 9.11 (dd, J = 5.3, 1.5 Hz, 1H), 7.83 (dd, J = 8.1, 1.5 Hz, 1H), 7.56 – 7.34 (m, 2H), 7.11 (dd, J = 8.1, 5.4 Hz, 1H), 6.51 (dd, J = 7.9, 5.4 Hz, 1H).

^{13}C NMR (75 MHz, CD_2Cl_2, major isomer): δ 176.2 (C-S), 167.1 (C-S), 157.1 (s(d), J_{WC} = 36.4 Hz, C_2H_2), 154.8 (s(d), J_{WC} = 28.3 Hz, C_2H_2), 142.3, 141.9, 140.6, 137.2, 133.3 (C-Cl), 133.0 (C-Cl), 120.7, 118.1.

IR (cm^{-1}): ν (W=O): 936

LRMS (EI-MS, m/z): Calcd for $C_{10}H_6Cl_2N_2OS_2W$ [M^+ - C_2H_2] 489.9, found 489.9

Anal. Calcd for $C_{12}H_8Cl_2N_2OS_2W$ · 0.4 CH_2Cl_2 · 0.2·MeCN · 0.1 n-C_5H_{12}: C, 28.30; H, 1.89; N, 5.46; S, 11.36. Found: C, 28.37; H, 1.67; N, 5.30; S, 11.00.

[WO(C_2H_2)(5-NO$_2$-6-MePyS)$_2$] (4c). [W(CO)(C_2H_2)(5-NO$_2$-6-Me-PyS)$_2$] (400 mg, 0.694 mmol, 1 equiv), (**2c**) and pyridine-*N*-oxide (69 mg, 0.729 mmol, 1.05 equiv) were placed in a 100 mL Schlenk flask and dissolved in 30 mL of dry CH_2Cl_2. The reaction was stirred with an attached bubbler for 20 min and the reaction mixture was dried *in vacuo*. The solid was dissolved in CH_2Cl_2 and filtered over Celite. The collected filtrate was overlaid with MeCN and after slow evaporation and removing the solvent mixture via cannula, bright orange crystals were collected in 75 % yield (293 mg).

^{1}H NMR (300 MHz, CD_2Cl_2) δ 11.44 (s, 1H, C≡CH), 11.15 (s, 1H, C≡CH), 8.32 (d, J = 8.8 Hz, 1H), 8.16 (d, J = 9.0 Hz, 1H), 7.21 (d, J = 8.8 Hz, 1H), 6.93 (d, J = 9.0 Hz, 1H), 2.98 (s, 3H, CH_3), 2.56 (s, 3H, CH_3).

^{13}C NMR (75 MHz, CD_2Cl_2) δ 181.5 (C-S), 179.8 (C-S), 160.2 (C_2H_2), 158.2 (C_2H_2), 155.4 (C-NO_2), 152.8 (C-NO_2), 143.5 (C-CH_3), 143.1 (C-CH_3), 135.7, 133.8, 127.4, 126.1, 126.1, 23.4 (CH_3), 21.0 (CH_3).

IR (cm^{-1}): ν (W=O): 937

Anal. Calcd. for $C_{14}H_{12}N_4O_5S_2W$: C, 29.80; H, 2.14; N, 9.93; S, 11.36. Found: C, 29.88; H, 2.13; N, 9.78; S, 11.49.

[WO(C_2H_2)(SPy)$_2$] (4d). In a 50 mL Schlenk tube, [W(CO)(C_2H_2)(SPy)$_2$] (405 mg, 0.882 mmol, 2 equiv) was mixed with pyridine-*N*-oxide (99 mg, 1.041 mmol, 1.18 equiv) and dissolved in 30 mL of CH_2Cl_2. The tube was equipped with a bubbler, wrapped in aluminum foil, and stirred for 7.5 h. The reaction mixture was dried and the solid was resuspended in 20 mL of CH_2Cl_2 and filtered through Celite. Thereafter, 8 mL of heptane were added to the filtrate and the mixture was slowly concentrated *in vacuo*. The formed precipitate was isolated via cannula and recrystallized again following the same procedure with 40 mL CH_2Cl_2 and 20 mL heptane to give 91% of pure product (359 mg) after drying.

^{1}H NMR (300 MHz, CD_2Cl_2, major isomer) δ 10.99 (s, 2H, C≡CH), 9.20 (d, J = 5.5 Hz, 1H), 7.77 – 7.67 (m, 1H), 7.52 (d, J = 5.5 Hz, 1H), 7.36 – 7.25 (m, 1H), 7.18 – 7.06 (m, 2H), 7.00 (d, J = 8.2, Hz, 1H), 6.58 – 6.44 (m, 1H).

^{13}C NMR (75 MHz, CD_2Cl_2, major isomer) δ 178.0 (C-S), 169.1 (C-S), 158.0 (s(d), J_{WC} = 35.1 Hz, C_2H_2), 155.0 (s(d), J_{WC} = 27.4 Hz, C_2H_2), 143.9, 143.4, 141.1, 137.6, 127.7, 127.4, 120.5, 117.7.

IR (cm^{-1}): ν (W=O): 937

Anal. Calcd for $C_{12}H_{10}N_2OS_2W$: C, 32.30; H, 2.26; N, 6.28; S, 14.37. Found: C, 32.44; H, 2.00; N, 6.31; S, 14.49.

3 Crystal Structure Determination

Crystal Structure Determination – General. All the single crystal measurements were performed on a Bruker APEX-II CCD diffractometer at 100 K using Mo K_{α} radiation with a wavelength of 0.71073Å from an Incoatec microfocus sealed tube equipped with a multilayer monochromator. Absorption corrections were made semi-empirically from equivalents. The structures were solved by direct methods (SHELXS-97)[6] and refined by full-matrix least-squares techniques against F^2 (SHELXL-2014/6)[7]. A weighting scheme of $w = 1/[\sigma^2(F_o^2)+(aP)^2+bP]$ where $P = (F_o^2+2F_c^2)/3$ was used. The non-hydrogen atoms were refined with anisotropic displacement parameters without any constraints. The H atoms of the aromatic rings were put at the external bisectors of the X–C–C angles at C–H distances of 0.95 Å and common isotropic displacement parameters were refined for the H atoms of the same ring. The H atoms of the methyl groups were refined with common isotropic displacement parameters for the H atoms of the same group and idealized geometries with tetrahedral angles, enabling rotation around the C–C bonds, and C–H distances of 0.98 Å. The positions of the H atoms of the ethenidyl groups and those of the ethyne ligands were taken from difference Fourier maps, the C–H distances were fixed to 0.95Å, and these H atoms were refined with common isotropic displacement parameters for the H atoms of the same C_2 unit without any constraints to the bond angles. The H atoms of the dichloromethane solvent molecule were refined with a common isotropic displacement parameter and idealized geometry with *approx*. tetrahedral angles and C–H distances of 0.99Å. Crystal data, data collection parameters, and structure refinement details are given in Tables **S1-S5**. Further refinement information, structure and bonding parameters, SHELXL .res, and .hkl files are given in the deposited CIF file which is available free of charge from The Cambridge Crystallographic Data Centre (CCDC 2103131-2103141).

Crystal Structure Determination of 2a. The carbonyl ligand and the ethyne ligand are disordered over two orientations and were refined with site occupation factors of 0.5 because the complex lies on a two-fold rotation axis. The H atoms of the pyridine ring, as well as those of the ethyne ligand, were put at the external bisectors of the C–C–X angles at C–H distances of 0.95Å, and common isotropic displacement parameters were refined for the H atoms of the same ligand.

Crystal Structure Determination of 2b. The H atoms of the ethyne ligand were put at the external bisectors of the W–C–C angles at C–H distances of 0.95Å and a common isotropic displacement parameter were refined for these H atoms.

Crystallographic data

Table S1. Crystallographic data and structure refinement for complexes **1a** and **1b**.

**Crystal data**	$W(CO)_3(4\text{-}MePyS)_2$ (**1a**)	$W(CO)_3(3\text{-}ClPyS)_2$ (**1b**)
CIF data code	MB64	RB33A
Empirical formula	$C_{15}H_{12}N_2O_3S_2W$	$C_{13}H_6Cl_2N_2O_3S_2W$
Formula weight	516.24	557.07
Crystal description	Needle, red	needle, orange
Crystal size	0.28 x 0.18 x 0.13mm	0.28 x 0.05 x 0.03mm
Temperature	100 K	100 K
Crystal system	monoclinic	monoclinic
Space group	C 2/c	C 2/c
a	30.255(4) Å	34.041(4) Å
b	7.2572(9) Å	6.9442(6) Å
c	17.973(3) Å	14.8081(15) Å
β	121.289(5) °	112.647(3) °
Volume	3372.3(8) $Å^3$	3230.6(5)$Å^3$
Z	8	8
Calc. Density	2.034 mg/m^3	2.291 mg/m^3
F (000)	1968	2096
Linear absorption coefficient μ	7.112 mm^{-1}	7.753 mm^{-1}
Max. and min. transmission	1.000 and 0.577	1.000 and 0.769
Unit cell determination	2.65° < θ < 35.77°	2.59° < θ < 33.81°
Reflections used	8318	7656
**Data collection**		
Θ range for data collection	2.28 to 35.00°	2.59 to 33.00°
Reflections collected/ unique	16307 / 7398	21155 / 6091
Significant unique reflections	6412 with I > 2σ(I)	5207 with I > 2σ(I)
R(int), R(sigma)	0.0316, 0.0410	0.0372, 0.0360
Completeness to θ_{max}	99.8%	99.9%
**Refinement**		
Data/ parameters/ restraints	7398 / 214 / 0	6091 / 210 / 0
Goodness-of-fit on F^2	1.034	1.008
Final R indices [I > 2σ(I)]	R1 = 0.0240, wR2 = 0.0550	R1 = 0.0241, wR2 = 0.0516
R indices (all data)	R1 = 0.0307, wR2 = 0.0574	R1 = 0.0322, wR2 = 0.0544
Weighting scheme param. a, b	0.0227, 0.0000	0.0254, 0.0000
Largest Δ/σ in last cycle	0.001	0.005
Largest diff. peak and hole	1.465 and -1.150e/$Å^3$	1.775 and -1.386e/$Å^3$
**CCDC no.**	2103131	2103132

Table S2. Crystallographic data and structure refinement for complexes **2a**, **2b,** and **2c**.

**Crystal data**	W(CO)(C_2H_2)(4-MePyS)$_2$ (**2a**)	W(CO)(C_2H_2)(3-ClPyS)$_2$ (**2b**)	W(CO)(C_2H_2)(5-NO_2-6MePyS)$_2$ (**2c**)
CIF data code	MB80B	RB56	MC3
Empirical formula	$C_{15}H_{14}N_2OS_2W$	$C_{13}H_8Cl_2N_2OS_2W \cdot 0.5C_6H_6$	$C_{15}H_{12}N_4O_5S_2W$
Formula weight	486.25	566.14	576.26
Crystal description	needle, green	block, blue	block, red
Crystal size [mm]	0.26 x 0.11 x 0.07	0.13 x 0.10 x 0.05	0.24 x 0.16 x 0.15
Temperature	100 K	100K	100K
Crystal system	orthorhombic	triclinic	monoclinic
Space group	P c c n	P -1	P 2_1/n
a	17.273(3)Å	8.0744(3)Å	9.7281(11)Å
b	6.7944(11)Å	10.3865(5)Å	14.3516(14)Å
c	13.531(2)Å	11.1903(5)Å	12.6889(13)Å
α		86.180(3)°	
β		88.018(2)°	97.766(4)°
γ		70.231(2)°	
Volume	1587.9(5)$Å^3$	881.13(7)$Å^3$	1755.3(3)$Å^3$
Z	4	2	4
Calc. Density	2.034 mg/m^3	2.134 mg/m^3	2.181 mg/m^3
F (000)	928	538	1104
Linear absorption coefficient μ	7.536 mm^{-1}	7.101 mm^{-1}	6.856 mm^{-1}
Max. and min. transmission	1.000 and 0.496	1.000 and 0.650	1.000 and 0.639
Unit cell determination	2.34° < θ < 34.22°	2.68° < θ < 32.40°	2.48° < θ < 40.76°
Reflections used	4760	6217	9949
**Data collection**			
Θ range for data collection	3.01 to 35.00°	1.82 to 31.99°	2.15 to 40.00°
Reflections collected/ unique	12659 / 3486	14850 / 6100	33669 / 10870
Significant unique reflections	2699 with I > 2σ(I)	5234 with I > 2σ(I)	8973 with I > 2σ(I)
R(int), R(sigma)	0.0457, 0.0430	0.0452, 0.0589	0.0414, 0.0444
Completeness to θ_{max}	99.9%	99.9%	99.9%
**Refinement**			
Data/ parameters/ restraints	3486 / 118 / 0	6100 / 221 / 0	10870 / 257 / 2
Goodness-of-fit on F^2	1.109	1.036	1.048
Final R indices [I > 2σ(I)]	R1 = 0.0311, wR2 = 0.0638	R1 = 0.0319, wR2 = 0.0687	R1 = 0.0290, wR2 = 0.0574
R indices (all data)	R1 = 0.0458, wR2 = 0.0695	R1 = 0.0429, wR2 = 0.0726	R1 = 0.0420, wR2 = 0.0621
Weighting scheme param. a, b	0.0000, 2.6994	0.0250, 0.4570	0.0000, 3.4077
Largest Δ/σ in last cycle	0.001	0.001	0.003
Largest diff. peak and hole	1.452, -1.226e/$Å^3$	1.715, -2.103e/$Å^3$	2.404, -2.255e/$Å^3$
**CCDC no.**	2103133	2103134	2103135

Table S3. Crystallographic data and structure refinement for complexes **3a** and **3b**.

**Crystal data**	$W(CO)(C_2H_2)$(4-MePyS)<br>(-CHCH-4-MePyS) (**3a**)	$W(CO)(C_2H_2)$(3-ClPyS)<br>(-CHCH-3-ClPyS) (**3b**)
CIF data code	MB66	RB32
Empirical formula	$C_{17}H_{16}N_2OS_2W$	$C_{15}H_{10}Cl_2N_2OS_2W \cdot CH_2Cl_2$
Formula weight	512.29	638.05
Crystal description	block, red	needle, purple
Crystal size	0.29 x 0.15 x 0.11 mm	0.19 x 0.16 x 0.03mm
Temperature	100 K	100 K
Crystal system	monoclinic	monoclinic
Space group	P 2_1/c	P 2_1/c
a	7.7201(8)Å	14.9107(13)Å
b	16.5422(18)Å	7.2712(7)Å
c	14.1069(14)Å	18.5418(16)Å
β	105.730(4)°	98.365(5)°
Volume	1734.1(3)$Å^3$	1988.9(3)$Å^3$
Z	4	4
Calc. Density	1.962 mg/m^3	2.131 mg/m^3
F (000)	984	1216
Linear absorption coefficient μ	6.906 mm^{-1}	6.564 mm^{-1}
Max. and min. transmission	1.000 and 0.623	1.000 and 0.565
Unit cell determination	2.88° < θ < 40.58°	2.76° < θ < 30.62°
Reflections used	9799	5895
**Data collection**		
Θ range for data collection	2.74 to 40.00°	2.22 to 30.00°
Reflections collected/ unique	38720 / 10718	13927 / 5796
Significant unique reflections	8811 with I > 2σ(I)	4191 with I > 2σ(I)
R(int), R(sigma)	0.0501, 0.0472	0.0610, 0.1016
Completeness to θ_{max}	99.9%	99.9%
**Refinement**		
Data/ parameters/ restraints	10718 / 228 / 4	5796 / 252 / 4
Goodness-of-fit on F^2	1.029	1.053
Final R indices [I > 2σ(I)]	R1 = 0.0293, wR2 = 0.0640	R1 = 0.0539, wR2 = 0.1340
R indices (all data)	R1 = 0.0411, wR2 = 0.0691	R1 = 0.0821, wR2 = 0.1441
Weighting scheme param. a, b	0.0000, 3.2321	0.0575, 4.2710
Largest Δ/σ in last cycle	0.001	0.002
Largest diff. peak and hole	2.336 and -2.380e/$Å^3$	2.084 and -1.724e/$Å^3$
**CCDC no.**	2103136	2103137

Table S4. Crystallographic data and structure refinement for complexes **4a** and **4b**.

**Crystal data**	$WO(C_2H_2)$ $(4\text{-MePyS})_2$ (**4a**)	$WO(C_2H_2)$ $(3\text{-ClPyS})_2$ (**4b**)
CIF data code	MB88	RB136
Empirical formula	$C_{14}H_{14}N_2OS_2W$	$C_{12}H_8Cl_2N_2OS_2W$
Formula weight	474.24	515.07
Crystal description	block, colorless	Block, yellow
Crystal size	0.16 x 0.13 x 0.10 mm	0.09 x 0.06 x 0.04 mm
Temperature	100 K	100 K
Crystal system	triclinic	triclinic
Space group	P -1	P -1
a	7.433(3)Å	7.5625(5)Å
b	7.930(3)Å	8.0843(5)Å
c	13.329(5)Å	12.8605(8)Å
α	94.702(8)°	85.869(4)°
β	98.065(7)°	82.241(4)°
γ	92.130(7)°	71.667(5)°
Volume	774.4(5)$Å^3$	739.15(8)$Å^3$
Z	2	2
Calc. Density	2.034 mg/m^3	2.314 mg/m^3
F (000)	452	484
Linear absorption coefficient μ	7.724 mm^{-1}	8.451mm^{-1}
Max. and min. transmission	1.000 and 0.688	0.745 and 0.491
Unit cell determination	2.58° < θ < 40.45°	2.86° < θ < 28.65°
Reflections used	8534	4217
**Data collection**		
Θ range for data collection	1.55 to 40.00°	2.66 to 29.00°
Reflections collected/ unique	18122 / 9584	17122 / 3918
Significant unique reflections	7852 with I > 2σ(I)	3465 with I > 2σ(I)
R(int), R(sigma)	0.0387, 0.0516	0.0996, 0.0763
Completeness to $θ_{max}$	99.9%	99.8 %
**Refinement**		
Data/ parameters/ restraints	9584 / 194 / 2	3918 / 190 / 2
Goodness-of-fit on F^2	1.039	1.046
Final R indices [I > 2σ(I)]	R1 = 0.0296, wR2 = 0.0678	R1 = 0.0363, wR2 = 0.0643
R indices (all data)	R1 = 0.0411, wR2 = 0.0720	R1 = 0.0448, wR2 = 0.0672
Weighting scheme param. a, b	0.0215, 0.0000	0.0000, 0.3712
Largest Δ/σ in last cycle	0.002	0.001
Largest diff. peak and hole	1.821 and -1.903$e/Å^3$	1.608 and -1.654$e/Å^3$
**CCDC no.**	2103138	2103139

Table S5. Crystallographic data and structure refinement for complexes **4c** and **4d**.

**Crystal data**	$WO(C_2H_2)(5\text{-}NO_2\text{-}6\text{-}MePyS)_2$ (**4c**)	$WO(C_2H_2)(PyS)_2$ (**4d**)
CIF data code	MC13B	CV73
Empirical formula	$C_{14}H_{12}N_4O_5S_2W$	$C_{12}H_{10}N_2OS_2W$
Formula weight	564.25	446.19
Crystal description	Block, yellow	Block, yellow
Crystal size	0.18 x 0.14 x 0.11 mm	0.22 x 0.16 x 0.11mm
Temperature	100 K	100 K
Crystal system	monoclinic	monoclinic
Space group	P 2_1/c	C 2/c
a	10.9324(5)Å	28.113(2)Å
b	9.7084(5)Å	7.4943(6)Å
c	16.8341(7)Å	14.9503(12)Å
β	101.481(2)°	119.501(4)°
Volume	1750.95(14)$Å^3$	2741.5(4)$Å^3$
Z	4	8
Calc. Density	2.140 mg/m^3	2.162 mg/m^3
F (000)	1080	1680
Linear absorption coefficient μ	6.870 mm^{-1}	8.719mm^{-1}
Max. and min. transmission	0.745 and 0.432	1.000 and 0.638
Unit cell determination	2.47° < θ < 29.56°	2.73° < θ < 35.30°
Reflections used	9910	9904
**Data collection**		
Θ range for data collection	2.43 to 30.00°	2.73 to 35.00°
Reflections collected/ unique	55673 / 5037	29918 / 6028
Significant unique reflections	4373 with I > 2σ(I)	5032 with I > 2σ(I)
R(int), R(sigma)	0.0941, 0.0559	0.0517, 0.0393
Completeness to θ_{max}	98.3%	100.0%
**Refinement**		
Data/ parameters/ restraints	5037 / 248 / 2	6028 / 172 / 2
Goodness-of-fit on F^2	1.054	1.027
Final R indices [I > 2σ(I)]	R1 = 0.0316, wR2 = 0.0609	R1 = 0.0272, wR2 = 0.0612
R indices (all data)	R1 = 0.0403, wR2 = 0.0649	R1 = 0.0359, wR2 = 0.0642
Weighting scheme param. a, b	0.0238, 0.9922	0.0287, 0.0000
Largest Δ/σ in last cycle	0.003	0.002
Largest diff. peak and hole	2.427 and -2.305e/$Å^3$	1.709 and -1.948e/$Å^3$
**CCDC no.**	2103140	2103141

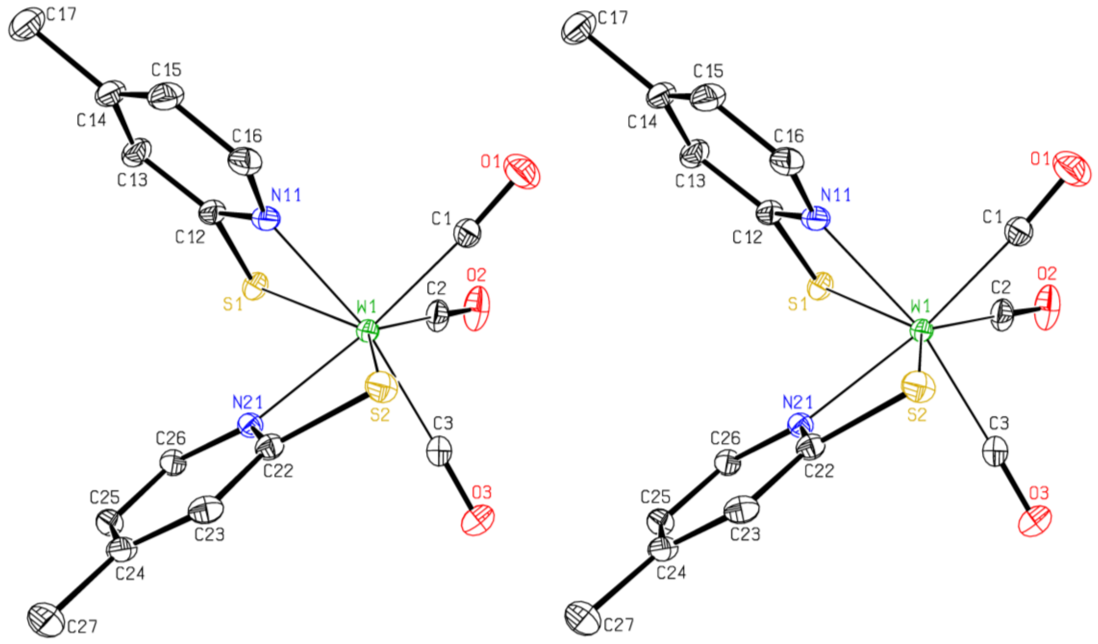

Figure S1. Stereoscopic ORTEP plot of **1a** showing the atomic numbering scheme. The probability ellipsoids are drawn at the 50% probability level. The H atoms were omitted for clarity.

Table S6. Selected bond lengths (Å) and angles (°) for complex **1a**.

W1-C1	1.985(2)	C2-W1-N21	135.98(8)
W1-C2	1.983(2)	C1-W1-S1	114.77(6)
W1-C3	2.013(2)	C2-W1-S2	143.32(6)
W1-N11	2.204(2)	S1-W1-S2	141.931(19)
W1-N21	2.2109(18)	O1-C1-W1	175.9(2)
W1-S1	2.5081(6)	O2-C2-W1	179.0(2)
W1-S2	2.5389(6)	O3-C3-W1	177.6(2)
C1-O1	1.145(3)	C12-S1-W1	81.88(7)
C2-O2	1.160(3)	C16-N11-C12	119.6(2)
C3-O3	1.145(3)	C16-N11-W1	136.45(15)
S1-C12	1.752(2)	C12-N11-W1	103.90(14)
S2-C22	1.754(2)	C22-S2-W1	80.83(7)
		C26-N21-C22	119.03(19)
C3-W1-N11	164.89(9)	C26-N21-W1	137.66(15)
C1-W1-N21	149.92(8)	C22-N21-W1	103.31(13)

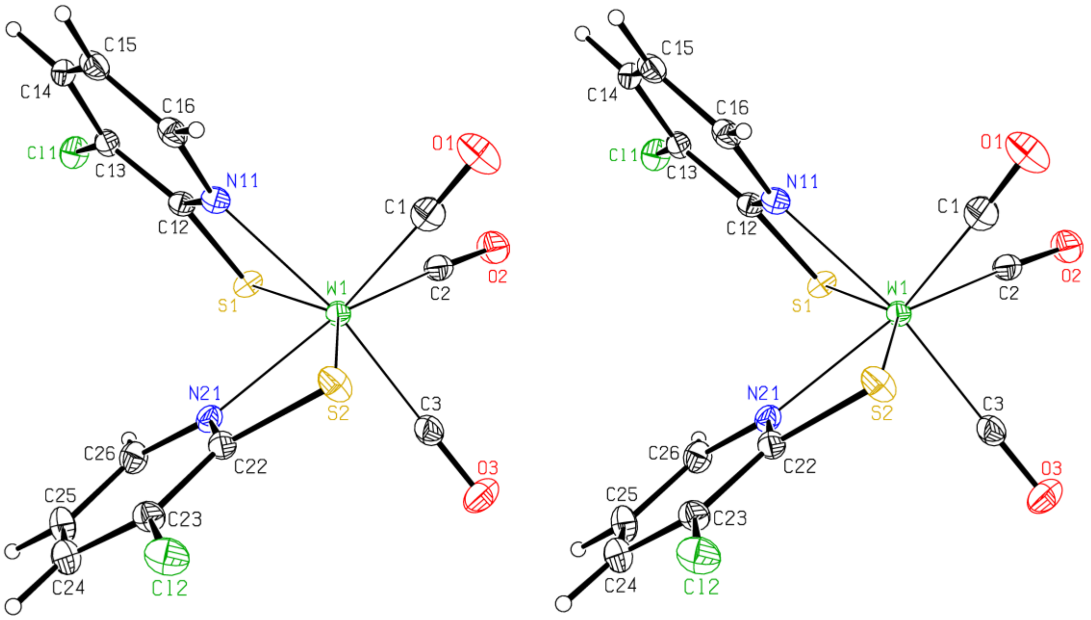

Figure S2. Stereoscopic ORTEP plot of **1b** showing the atomic numbering scheme. The probability ellipsoids are drawn at the 50% probability level. The H atoms are drawn with arbitrary radii.

Table S7. Selected bond lengths (Å) and angles (°) for complex **1b**.

W1-C1	2.008(3)
W1-C2	2.001(3)
W1-C3	1.998(3)
W1-N11	2.220(2)
W1-N21	2.240(2)
W1-S1	2.5089(6)
W1-S2	2.5698(7)
C1-O1	1.147(3)
C2-O2	1.153(3)
C3-O3	1.144(3)
S1-C12	1.742(3)
S2-C22	1.733(3)
C3-W1-N11	170.50(9)
C1-W1-N21	135.36(10)

C2-W1-N21	149.82(9)
C1-W1-S1	128.32(8)
C2-W1-S2	136.53(8)
S1-W1-S2	147.30(2)
O1-C1-W1	176.7(2)
O2-C2-W1	176.8(2)
O3-C3-W1	179.2(3)
C12-S1-W1	81.72(9)
C16-N11-C12	120.2(2)
C16-N11-W1	137.48(18)
C12-N11-W1	102.35(16)
C22-S2-W1	80.46(9)
C26-N21-C22	120.5(2)
C26-N21-W1	137.40(18)
C22-N21-W1	102.13(16)

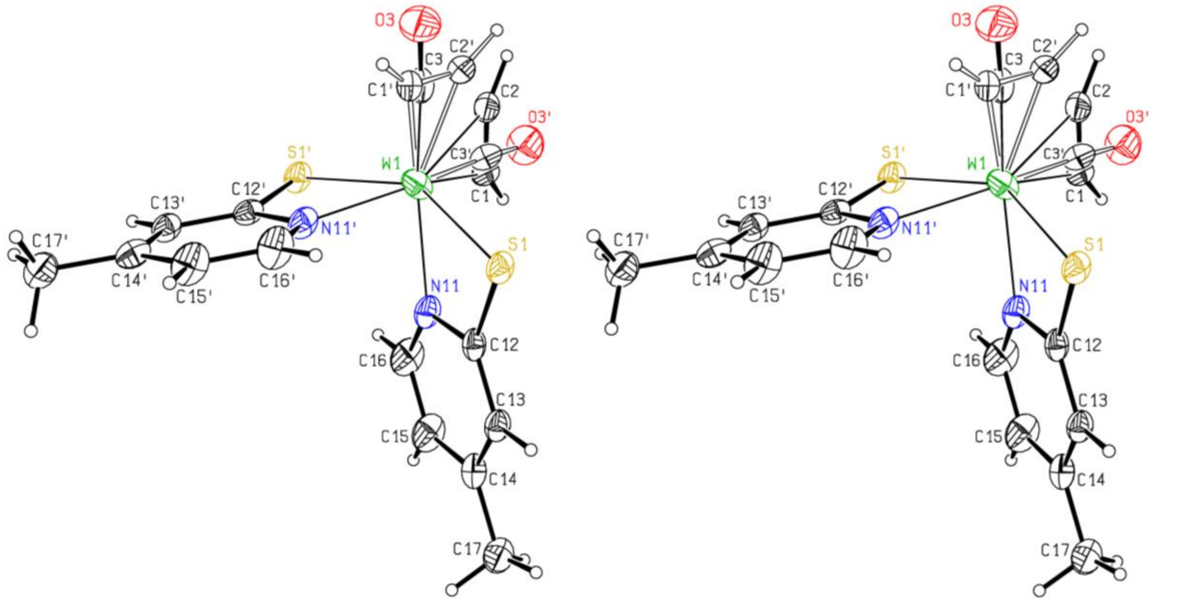

Figure S3. Stereoscopic ORTEP plot of **2a** showing the atomic numbering scheme. The probability ellipsoids are drawn at the 50% probability level. The H atoms are drawn with arbitrary radii. One arrangement of the disordered ligands is drawn with open bonds.

Table S8. Selected bond lengths [Å] and angles [°] for **2a**.

W1-C1	2.090(13)
W1-C2	2.078(7)
W1-C3	1.884(16)
W1-N11	2.228(3)
W1-S1	2.4788(8)
C1-C2	1.302(12)
C3-O3	1.142(16)
S1-C12	1.749(3)

C3-W1-N11	161.1(5)
C1-W1-N11	152.8(4)
C2-W1-N11	154.7(2)
S1-W1-S1	146.18(4)
O3-C3-W1	176.6(10)
C12-S1-W1	83.10(9)
C16-N11-C12	118.6(3)
C16-N11-W1	138.4(2)
C12-N11-W1	102.88(19)

C1-C2-W1-C3	177.8(9)
C2-C1-W1-C3	-2.2(9)
W1-N11-C12-S1	0.2(2)
W1-S1-C12-N11	-0.17(18)

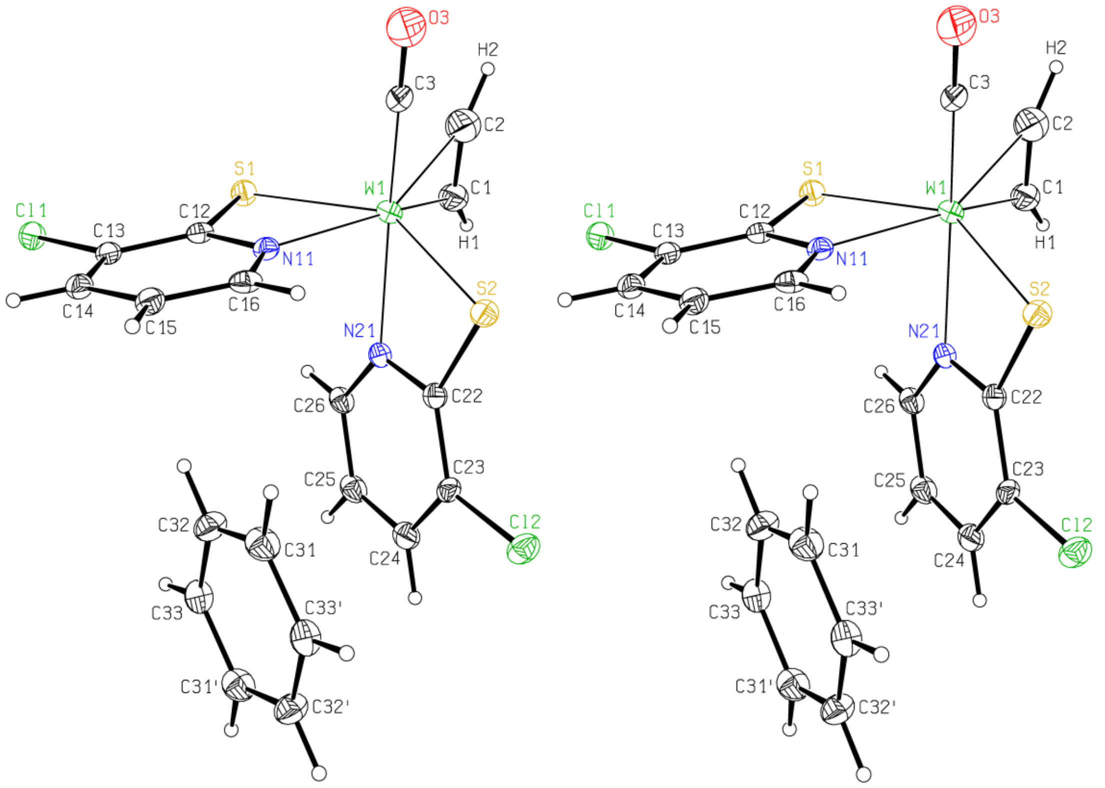

Figure S4. Stereoscopic ORTEP plot of **2b** showing the atomic numbering scheme. The probability ellipsoids are drawn at the 50% probability level. The H atoms are drawn with arbitrary radii

Table S9. Selected bond lengths [Å] and angles [°] for **2b**.

W1-C1	2.020(4)	S1-W1-S2	144.93(3)
W1-C2	2.048(5)	N11-W1-N21	81.44(10)
W1-C3	1.962(4)	C1-W1-N11	155.22(14)
W1-N11	2.244(3)	C2-W1-N11	152.47(16)
W1-N21	2.231(3)	C3-W1-N21	159.61(14)
W1-S1	2.5243(9)	O3-C3-W1	178.7(4)
W1-S2	2.4427(10)	C12-S1-W1	81.82(12)
C1-C2	1.286(6)	C16-N11-C12	121.0(3)
C3-O3	1.148(5)	C16-N11-W1	136.9(2)
S1-C12	1.730(4)	C12-N11-W1	102.1(2)
S2-C22	1.753(4)	C22-S2-W1	82.64(12)
		C26-N21-C22	121.1(3)
		C26-N21-W1	137.9(3)
		C22-N21-W1	101.0(2)

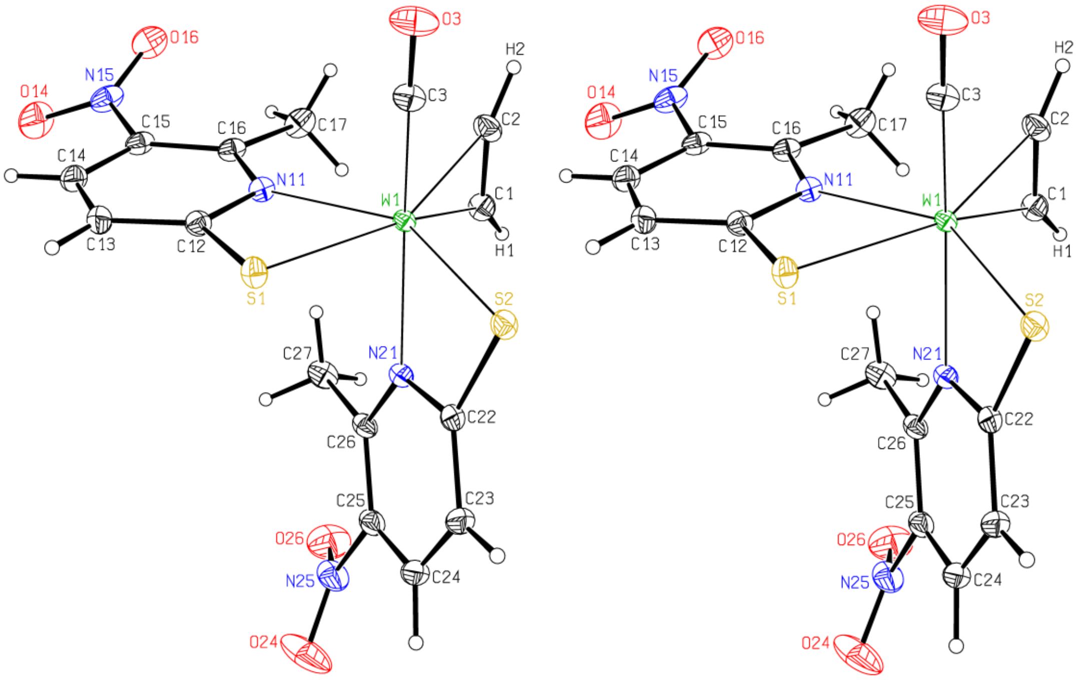

Figure S5. Stereoscopic ORTEP plot of **2c** showing the atomic numbering scheme. The probability ellipsoids are drawn at the 50% probability level. The H atoms are drawn with arbitrary radii.

Table S10. Selected bond lengths [Å] and angles [°] for **2c**.

W1-C1	2.023(2)
W1-C2	2.044(2)
W1-C3	1.969(2)
W1-N11	2.2348(18)
W1-N21	2.2491(17)
W1-S1	2.5872(6)
W1-S2	2.4024(6)
C1-C2	1.313(3)
C3-O3	1.151(3)
S1-C12	1.710(2)
S2-C22	1.748(2)

C1-W1-S1	159.79(7)
C2-W1-S1	154.69(7)
C3-W1-N21	159.99(8)
N11-W1-S2	145.80(5)
C2-C1-H1	140.4(17)
C1-C2-H2	145.6(6)
O3-C3-W1	178.8(2)
C12-S1-W1	80.87(7)
C12-N11-C16	120.65(19)
C12-N11-W1	102.65(13)
C16-N11-W1	136.67(15)
C22-S2-W1	83.64(8)
C22-N21-C26	121.77(19)
C22-N21-W1	99.40(13)
C26-N21-W1	138.75(15)

C1-C2-W1-C3	179.0(3)
C2-C1-W1-C3	-1.04(19)

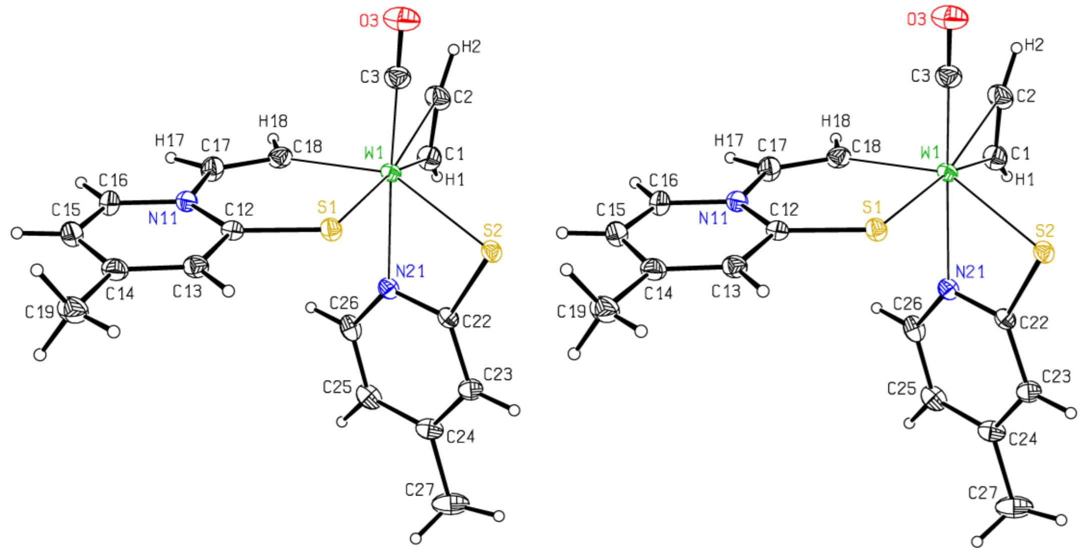

Figure S6. Stereoscopic ORTEP plot of **3a** showing the atomic numbering scheme. The probability ellipsoids are drawn at the 50% probability level. The H atoms are drawn with arbitrary radii.

Table S11. Selected bond lengths [Å] and angles [°] for **3a**.

W1-C1	2.031(2)	C1-C2-H2	147.6(5)
W1-C2	2.0505(19)	O3-C3-W1	178.4(2)
W1-C3	1.987(2)	C12-S1-W1	117.85(7)
W1-C18	2.104(2)	C18-C17-N11	128.36(19)
W1-N21	2.2360(19)	C18-C17-H17	124.3(15)
W1-S1	2.5737(6)	N11-C17-H17	107.3(16)
W1-S2	2.4796(6)	C17-C18-W1	143.02(16)
C1-C2	1.308(3)	C17-C18-H18	103.2(6)
C3-O3	1.157(3)	W1-C18-H18	113.6(6)
S1-C12	1.720(2)	C22-S2-W1	82.42(7)
N11-C17	1.435(3)	C26-N21-C22	119.6(2)
C17-C18	1.331(3)	C26-N21-W1	138.06(16)
S2-C22	1.759(2)	C22-N21-W1	102.17(13)

C1-W1-S1	164.71(7)	C1-C2-W1-C3	-177.36(18)
C2-W1-S1	157.93(7)	C2-C1-W1-C3	2.68(17)
C3-W1-N21	164.99(8)	C17-C18-W1-C3	-86.7(3)
C18-W1-S2	148.64(6)	C12-N11-C17-C18	-2.6(4)
S1-W1-S2	74.995(18)	C16-N11-C17-C18	176.9(2)
C2-C1-H1	139.5(14)	N11-C17-C18-W1	2.8(5)

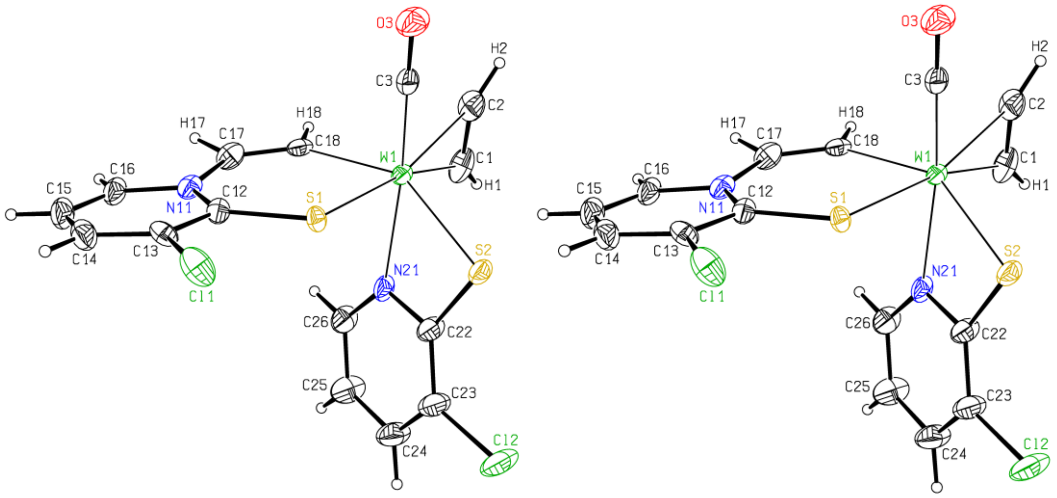

Figure S7. Stereoscopic ORTEP plot of **3b** showing the atomic numbering scheme. The probability ellipsoids are drawn at the 50% probability level. The H atoms are drawn with arbitrary radii. The solvent molecule was omitted for clarity.

Table S12. Selected bond lengths [Å] and angles [°] for **3b**.

W1-C1	2.048(5)	O3-C3-W1	178.5(8)
W1-C2	2.071(5)	C12-S1-W1	117.4(3)
W1-C3	1.963(8)	C12-N11-C16	119.5(8)
W1-C18	2.101(6)	C12-N11-C17	126.1(6)
W1-N21	2.225(7)	C16-N11-C17	114.4(7)
W1-S1	2.565(2)	C18-C17-N11	128.4(5)
W1-S2	2.496(2)	C18-C17-H17	126.8(12)
C1-C2	1.314(10)	N11-C17-H17	104.4(11)
C3-O3	1.181(9)	C17-C18-W1	142.3(4)
S1-C12	1.734(8)	C17-C18-H18	109.2(8)
N11-C17	1.424(10)	W1-C18-H18	107.9(7)
C13-Cl1	1.733(9)	C22-S2-W1	82.0(3)
C17-C18	1.311(8)	C22-N21-C26	120.3(7)
S2-C22	1.748(9)	C22-N21-W1	102.3(5)
C23-Cl2	1.765(10)	C26-N21-W1	137.3(6)

C1-W1-S1	167.2(3)	C1-C2-W1-C3	-177.3(6)
C2-W1-S1	155.4(2)	C2-C1-W1-C3	2.8(5)
C18-W1-S2	146.19(13)	C17-C18-W1-C3	-92.3(7)
C3-W1-N21	163.1(3)	C17-N11-C12-S1	-10.3(10)
C18-W1-N21	89.4(2)	C12-N11-C17-C18	5.4(11)
S1-W1-S2	74.62(7)	C16-N11-C17-C18	-177.4(7)
C2-C1-H1	137.3(17)	N11-C17-C18-W1	9.3(12)
C1-C2-H2	139.0(5)	W1-N21-C22-S2	0.4(7)

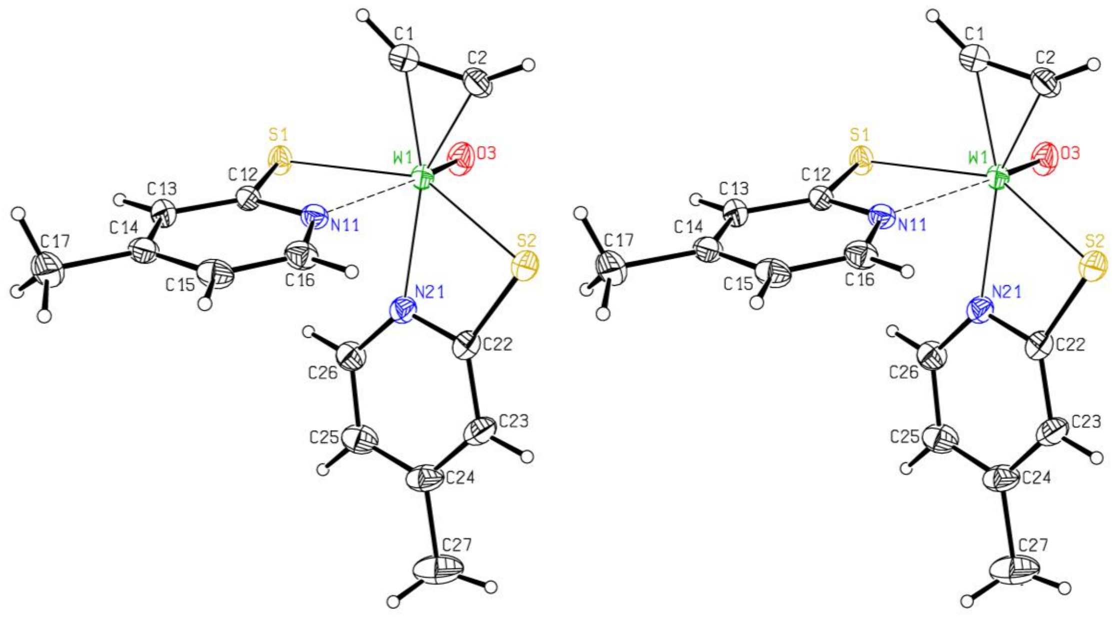

Figure S8. Stereoscopic ORTEP plot of **4a** showing the atomic numbering scheme. The probability ellipsoids are drawn at the 50% probability level. The H atoms are drawn with arbitrary radii. The rather long W–N distance [W1–N11 2.3464(18)Å] is plotted with a dashed line.

Table S13. Selected bond lengths [Å] and angles [°] for **4a**.

W1-C1	2.095(2)	S1-W1-S2	147.35(2)
W1-C2	2.093(2)	C12-S1-W1	83.94(7)
W1-O3	1.7204(17)	C12-N11-C16	119.39(18)
W1-N11	2.3464(18)	C12-N11-W1	100.72(12)
W1-N21	2.219(2)	C16-N11-W1	139.76(14)
W1-S1	2.5269(10)	C22-S2-W1	82.92(7)
W1-S2	2.4407(8)	C26-N21-C22	119.9(2)
C1-C2	1.280(3)	C26-N21-W1	137.90(15)
S1-C12	1.745(2)	C22-N21-W1	102.15(14)
S2-C22	1.763(2)		
		C1-C2-W1-O3	97.22(16)
C1-W1-N21	156.57(9)	C2-C1-W1-O3	-92.05(16)
C2-W1-N21	145.90(8)	C1-C2-W1-S1	-3.76(18)
O3-W1-N11	154.46(7)	C2-C1-W1-S1	176.71(15)
N11-W1-N21	73.60(7)		

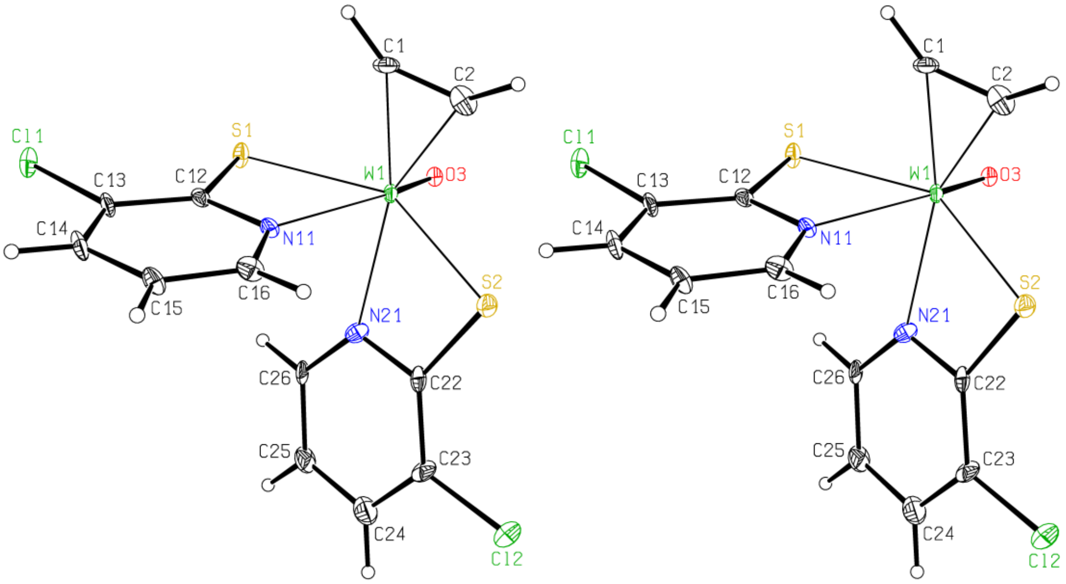

Figure S9. Stereoscopic ORTEP plot of 4b showing the atomic numbering scheme. The probability ellipsoids are drawn at the 50% probability level, the H atoms are drawn with arbitrary radii.

Table S14. Selected bond lengths [Å] and angles [°] for **4b**.

W1-C1	2.083(3)
W1-C2	2.094(4)
W1-O3	1.701(3)
W1-N11	2.306(4)
W1-N21	2.242(4)
W1-S1	2.5253(12)
W1-S2	2.4350(12)
C1-C2	1.274(5)
S1-C12	1.726(4)
C13-Cl1	1.725(5)
S2-C22	1.753(5)
C23-Cl2	1.725(5)
C1-W1-N21	159.88(16)
C2-W1-N21	147.69(17)
O3-W1-N11	154.27(14)
S1-W1-S2	146.59(4)
C12-S1-W1	83.10(16)
C12-N11-C16	120.2(4)
C12-N11-W1	100.8(3)
C16-N11-W1	139.1(3)
C22-S2-W1	82.99(17)
C22-N21-C26	120.8(5)
C22-N21-W1	100.7(3)
C26-N21-W1	138.4(3)
C1-C2-W1-O3	98.5(3)
C2-C1-W1-O3	-90.5(3)
C1-C2-W1-S1	-2.1(2)
C2-C1-W1-S1	178.1(2)
C1-C2-W1-S2	-155.4(3)
C2-C1-W1-S2	27.0(3)

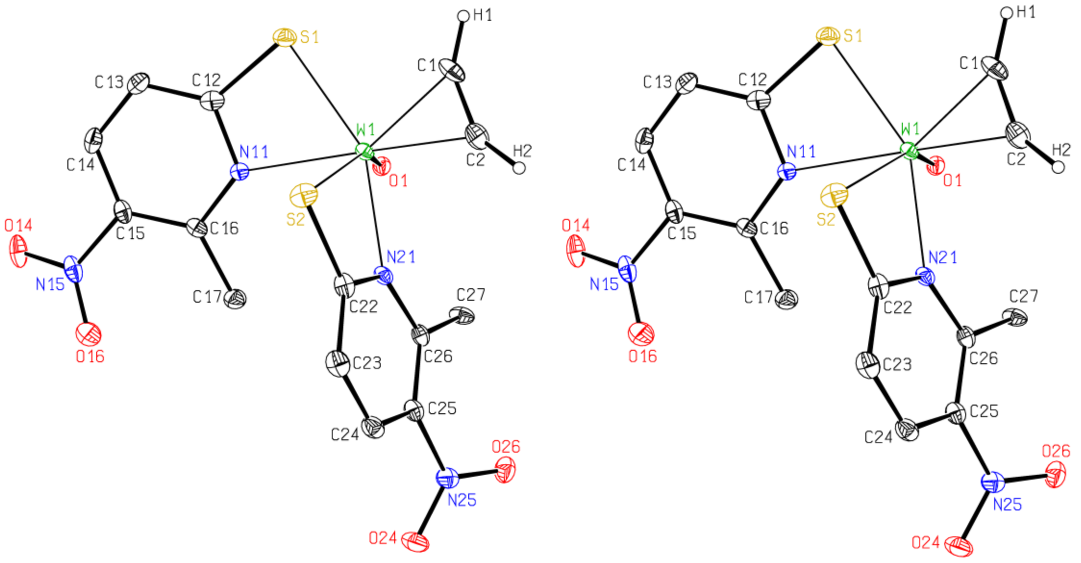

Figure S10. Stereoscopic ORTEP plot of **4c** showing the atomic numbering scheme. The probability ellipsoids are drawn at the 50% probability level. The H atoms of the ethyne ligand are drawn with arbitrary radii, the other H atoms were omitted for clarity.

Table S15. Selected bond lengths [Å] and angles [°] for **4c**.

W1-O1	1.710(2)
W1-C1	2.068(3)
W1-C2	2.084(3)
W1-N11	2.279(3)
W1-N21	2.225(2)
W1-S1	2.4153(8)
W1-S2	2.6283(8)
C1-C2	1.258(5)
S1-C12	1.741(3)
S2-C22	1.712(3)

C1-W1-N11	146.73(11)
C2-W1-N11	166.70(11)
N21-W1-S1	147.12(7)
O1-W1-S2	152.43(6)
C12-S1-W1	84.54(11)
C12-N11-C16	121.9(3)
C12-N11-W1	99.85(19)
C16-N11-W1	138.2(2)
C22-S2-W1	79.97(10)
C22-N21-C26	121.8(2)
C22-N21-W1	104.11(18)
C26-N21-W1	134.0(2)

C1-C2-W1-O1	88.2(2)
C2-C1-W1-O1	-100.5(2)
C1-C2-W1-N21	-177.9(2)
C2-C1-W1-N21	2.3(2)
C1-C2-W1-S1	-28.2(2)
C2-C1-W1-S1	153.8(2)
W1-N11-C12-S1	0.1(2)
W1-S1-C12-N11	-0.1(2)
C14-C15-N15-O14	19.5(4)
C16-C15-N15-O16	20.6(4)
W1-N21-C22-S2	-3.6(2)
W1-S2-C22-N21	3.04(19)
C24-C25-N25-O24	27.8(4)
C26-C25-N25-O26	29.4(4)

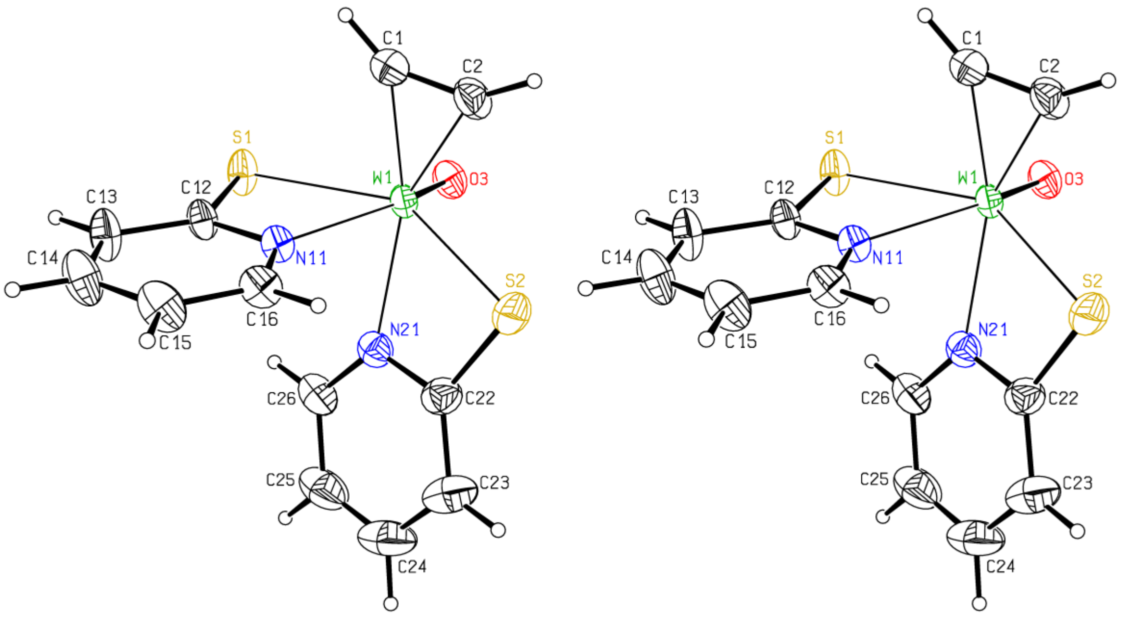

Figure S11. Stereoscopic ORTEP plot of **4d** showing the atomic numbering scheme. The probability ellipsoids are drawn at the 50% probability level. The H atoms are drawn with arbitrary radii.

Table S16. Selected bond lengths [Å] and angles [°] for **4d**.

W1-C1	2.073(2)
W1-C2	2.087(3)
W1-O3	1.712(2)
W1-N11	2.314(2)
W1-N21	2.221(2)
W1-S1	2.5158(7)
W1-S2	2.4391(8)
C1-C2	1.260(4)
S1-C12	1.734(3)
S2-C22	1.757(3)
C1-W1-N21	158.65(10)
C2-W1-N21	148.11(11)
O3-W1-N11	154.09(8)
N11-W1-N21	74.97(8)
S1-W1-S2	145.80(3)
C12-S1-W1	83.70(8)
C16-N11-C12	120.0(2)
C16-N11-W1	138.81(19)
C12-N11-W1	101.14(14)
C22-S2-W1	82.97(11)
C26-N21-C22	120.7(3)
C26-N21-W1	137.4(2)
C22-N21-W1	101.86(19)
C1-C2-W1-O3	98.3(2)
C2-C1-W1-O3	-90.6(2)
C1-C2-W1-S1	-1.8(3)
C2-C1-W1-S1	178.4(2)

4 NMR spectra

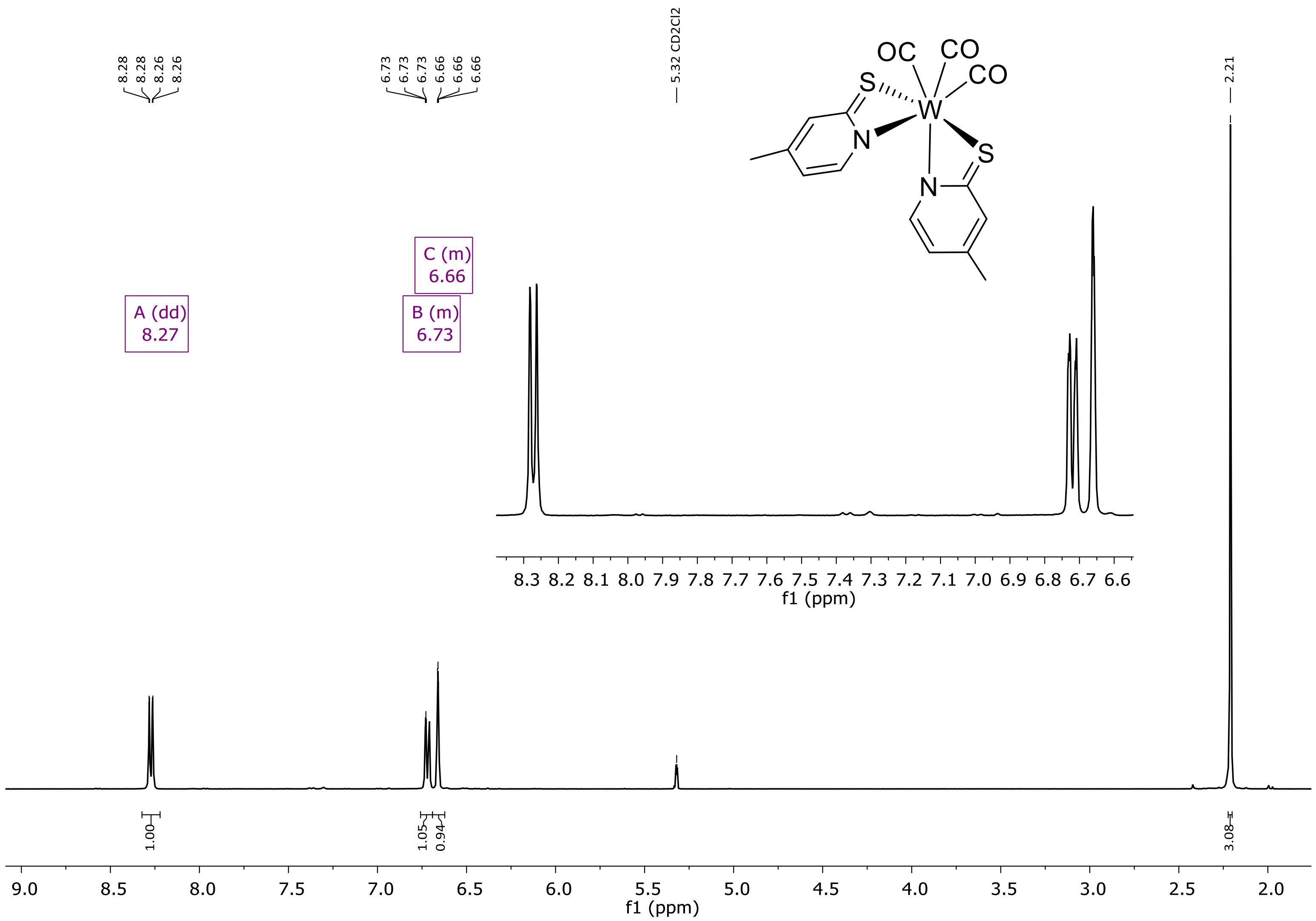

Figure S12. ^{1}H NMR spectrum of [W(CO)$_3$(4-MePyS)$_2$] (**1a**) in CD_2Cl_2.

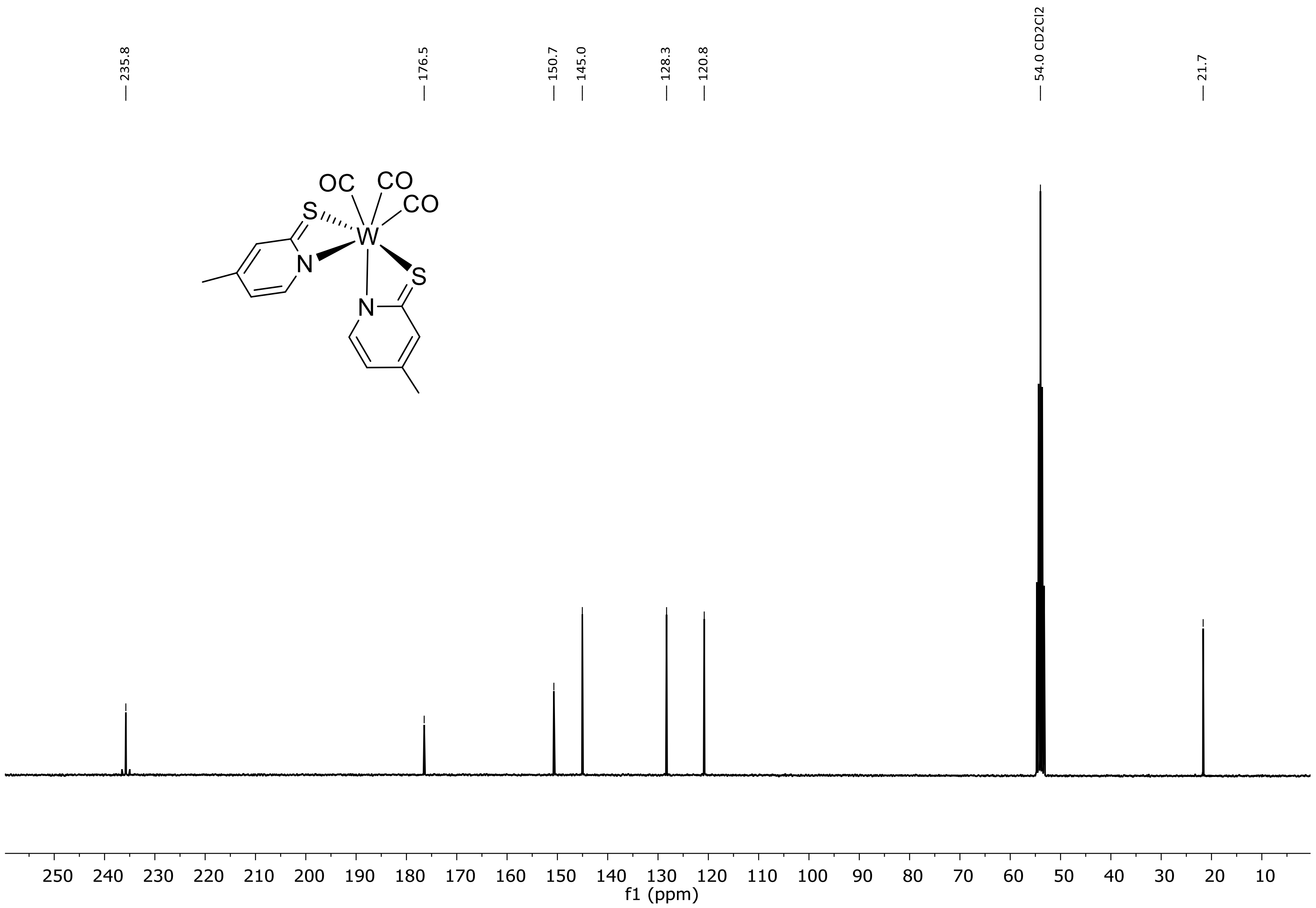

Figure S13. ^{13}C NMR spectrum of [W(CO)$_3$(4-MePyS)$_2$] (**1a**) in CD_2Cl_2.

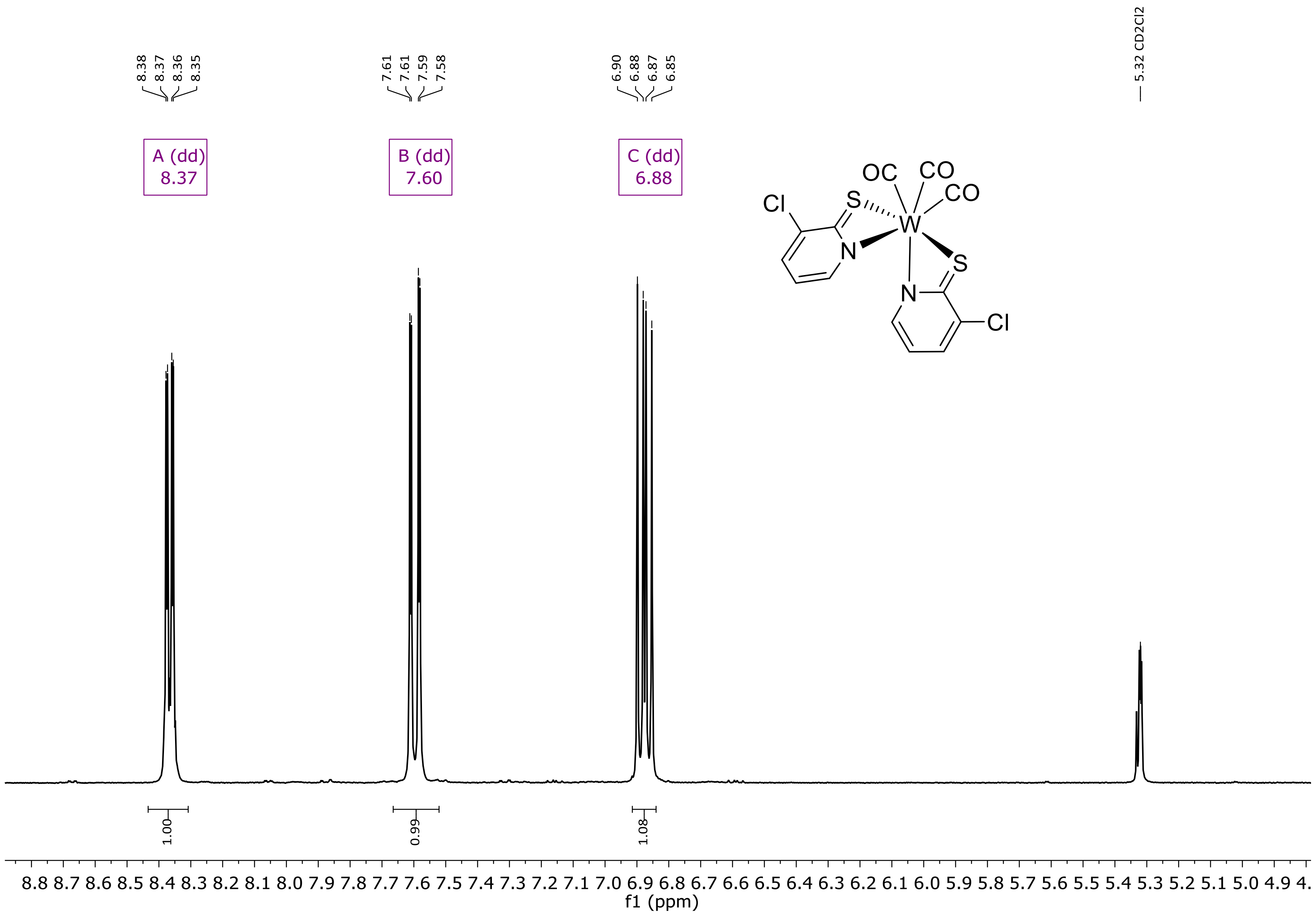

Figure S14. ^{1}H NMR spectrum of $[W(CO)_3(3\text{-}ClPyS)_2]$ (**1b**) in CD_2Cl_2.

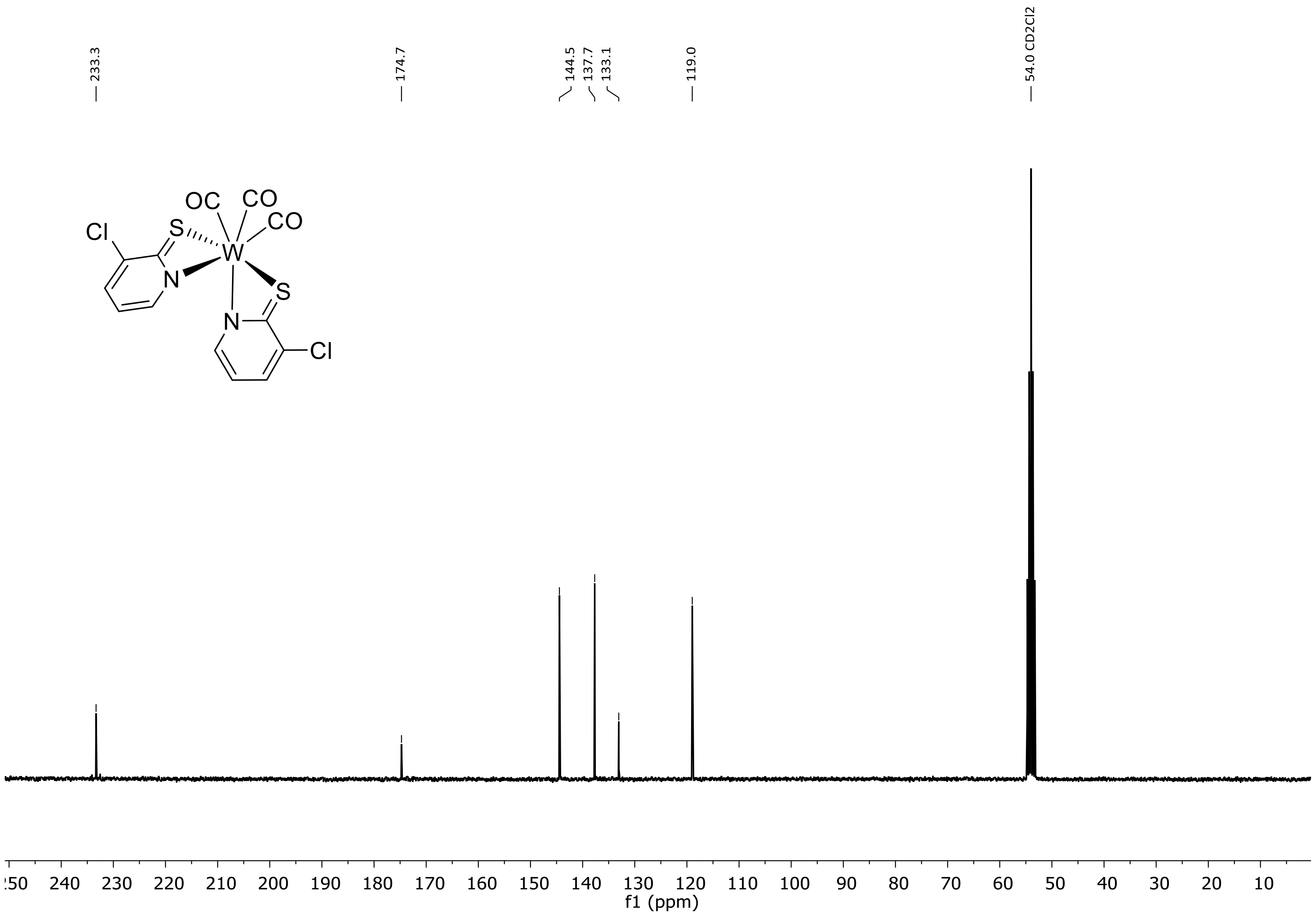

Figure S15. ^{13}C NMR spectrum of [W(CO)$_3$(3-ClPyS)$_2$] (**1b**) in CD_2Cl_2.

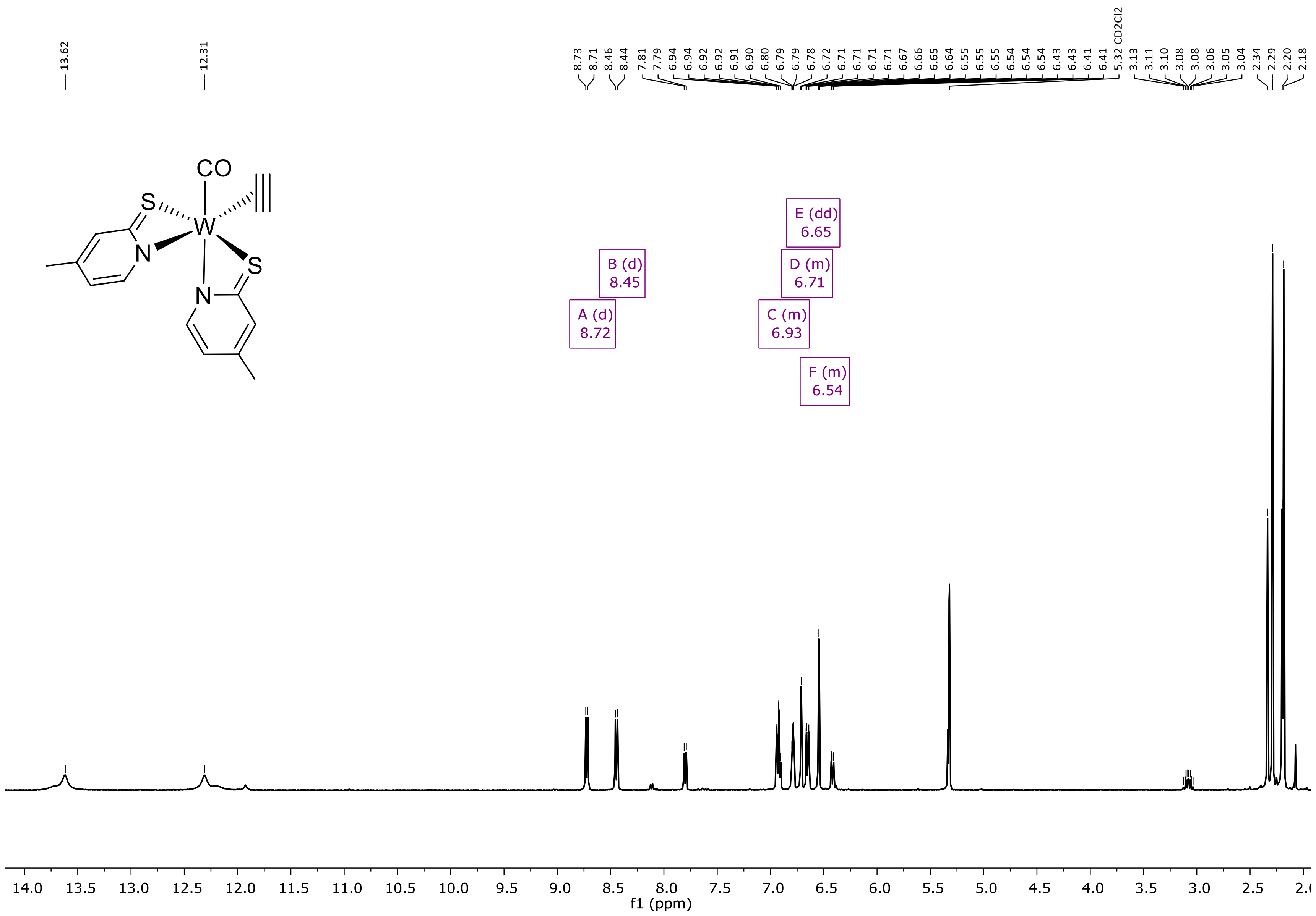

Figure S16. ^{1}H NMR spectrum of [W(CO)(C_2H_2)(4-MePyS)$_2$] (**2a**) in CD_2Cl_2. Two isomers are present.

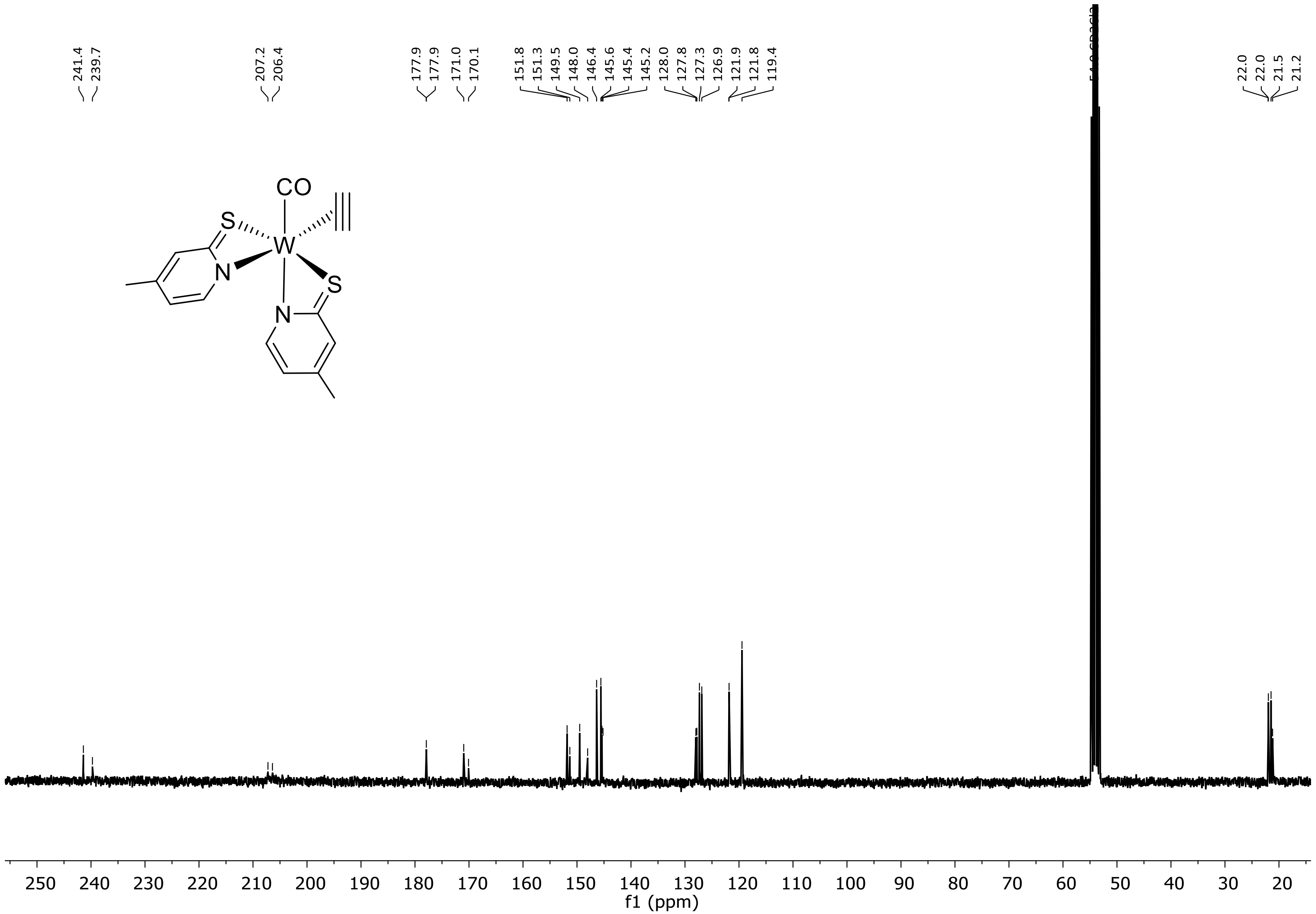

Figure S17. ^{13}C NMR spectrum of [W(CO)(C_2H_2)(4-MePyS)$_2$] (**2a**) in CD_2Cl_2. Two isomers are present.

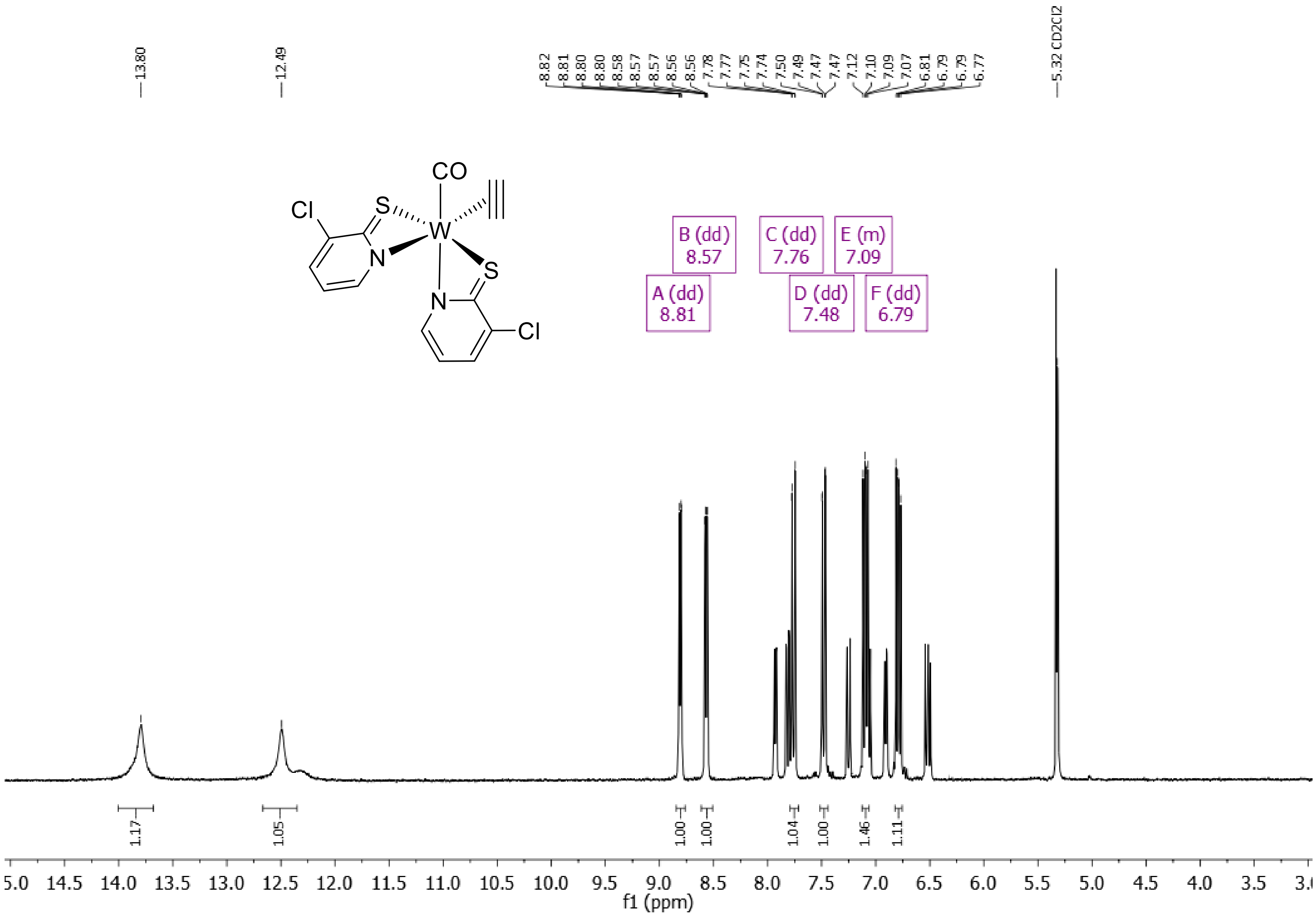

Figure S18. ^{1}H NMR spectrum of [W(CO)(C_2H_2)(3-ClPyS)$_2$] (**2b**) in CD_2Cl_2. Two isomers are present.

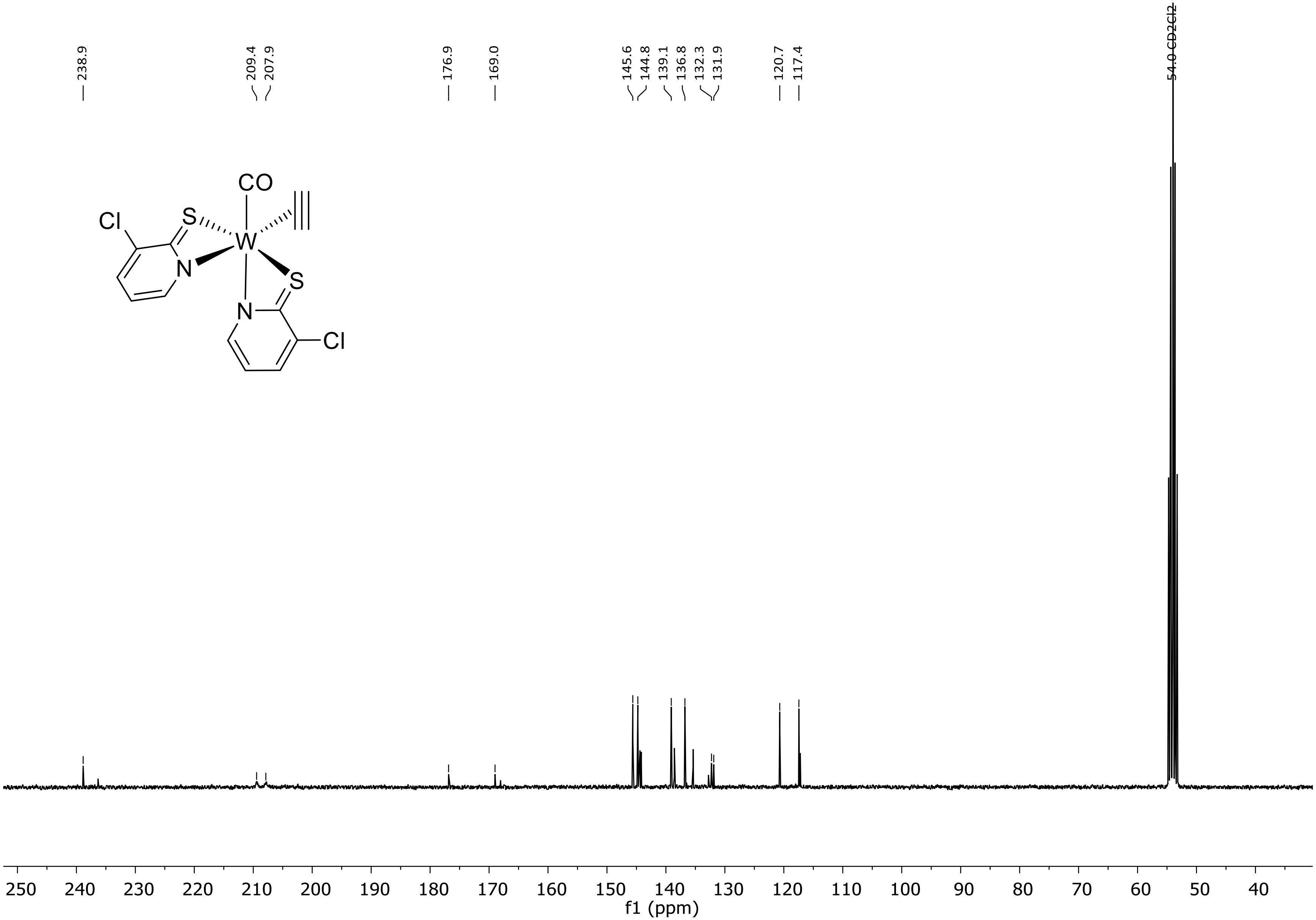

Figure S19: ^{13}C NMR spectrum of [W(CO)(C_2H_2)(3-ClPyS)$_2$] (**2b**) in CD_2Cl_2. Two isomers are present.

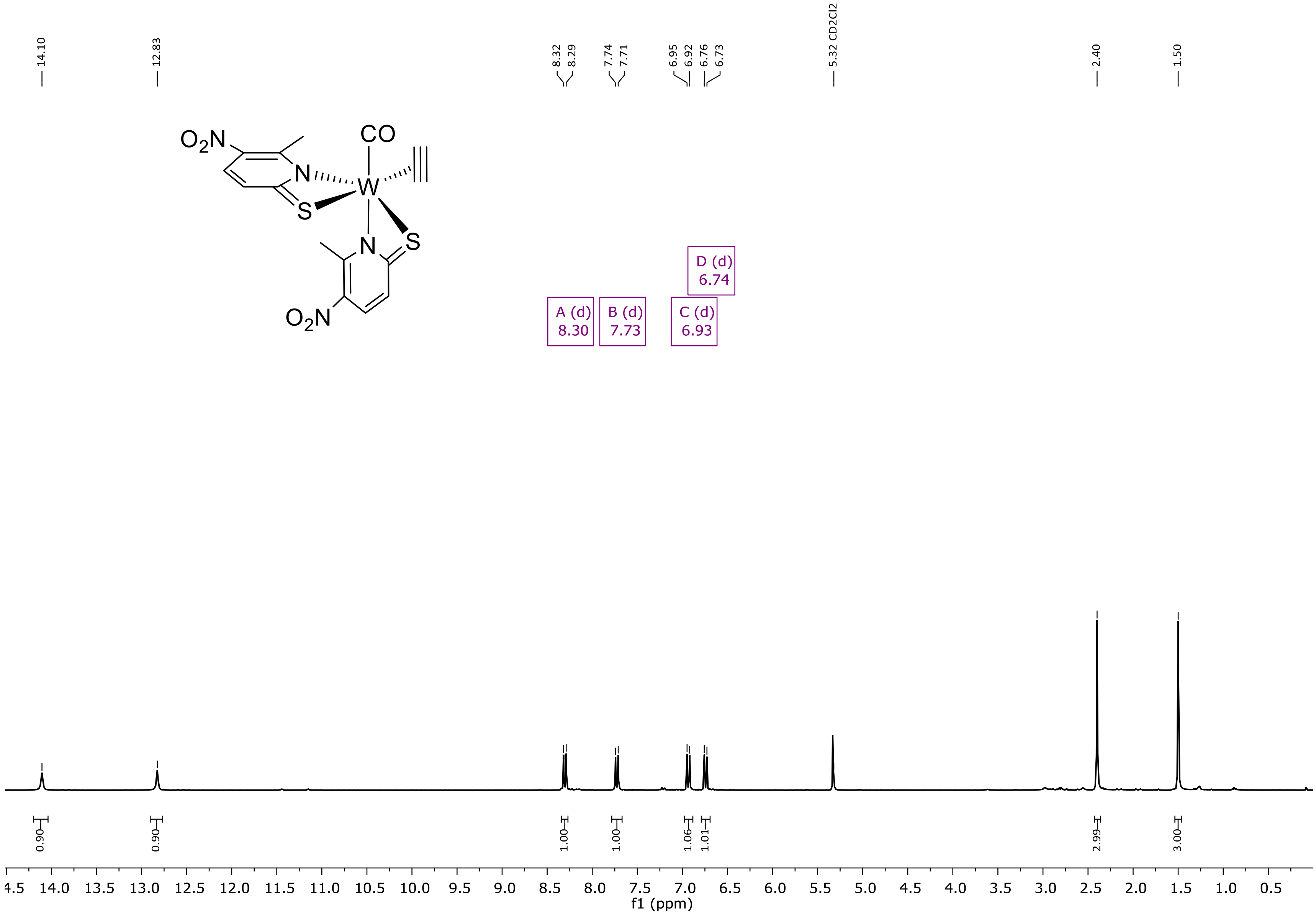

Figure S20. ^{1}H NMR spectrum of [W(CO)(C_2H_2)(5-NO_2-6-MePyS)$_2$] (**2c**) in CD_2Cl_2.

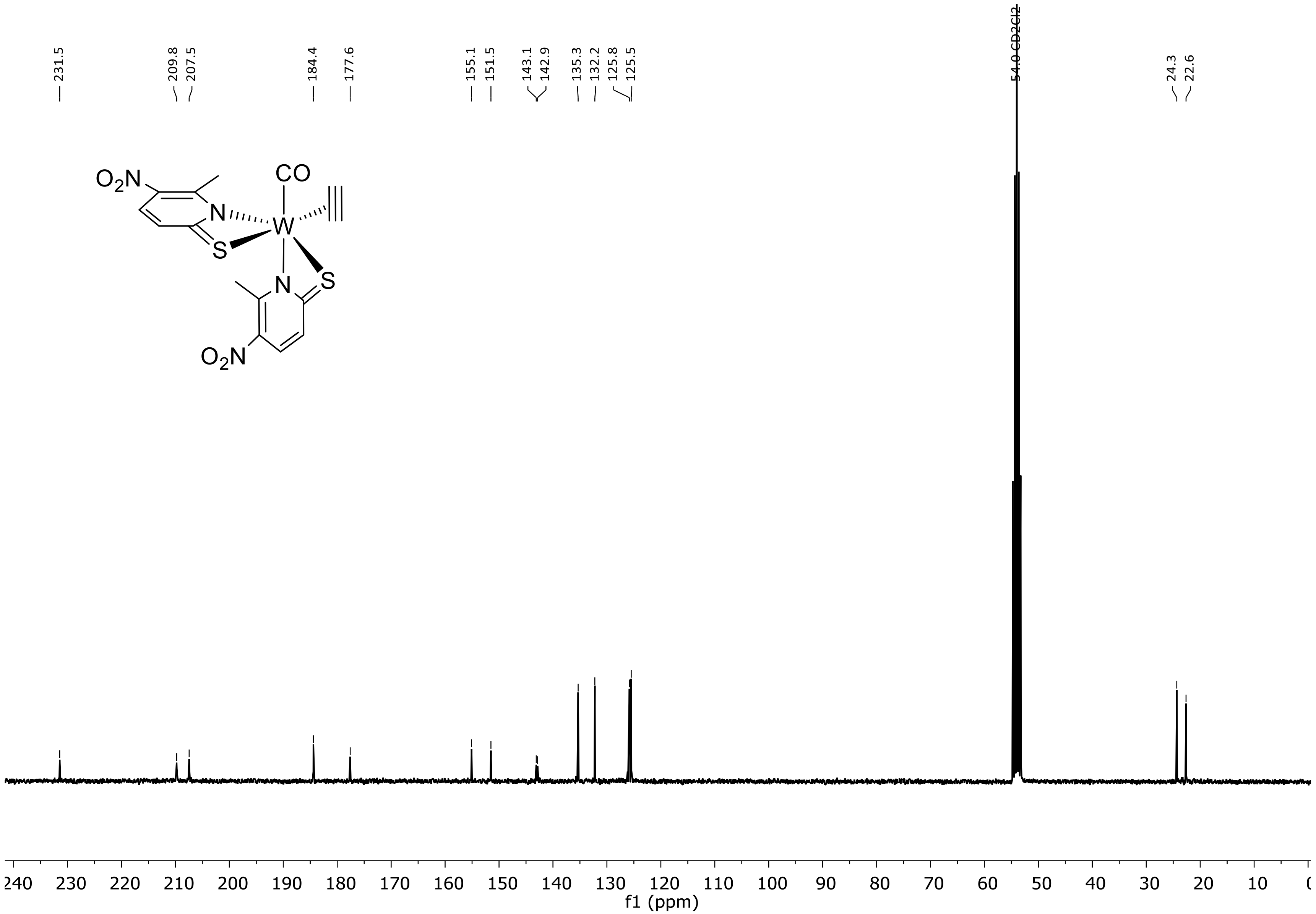

Figure S21. ^{13}C NMR spectrum of $[W(CO)(C_2H_2)(5\text{-}NO_2\text{-}6\text{-}MePyS)_2]$ (**2c**) in CD_2Cl_2.

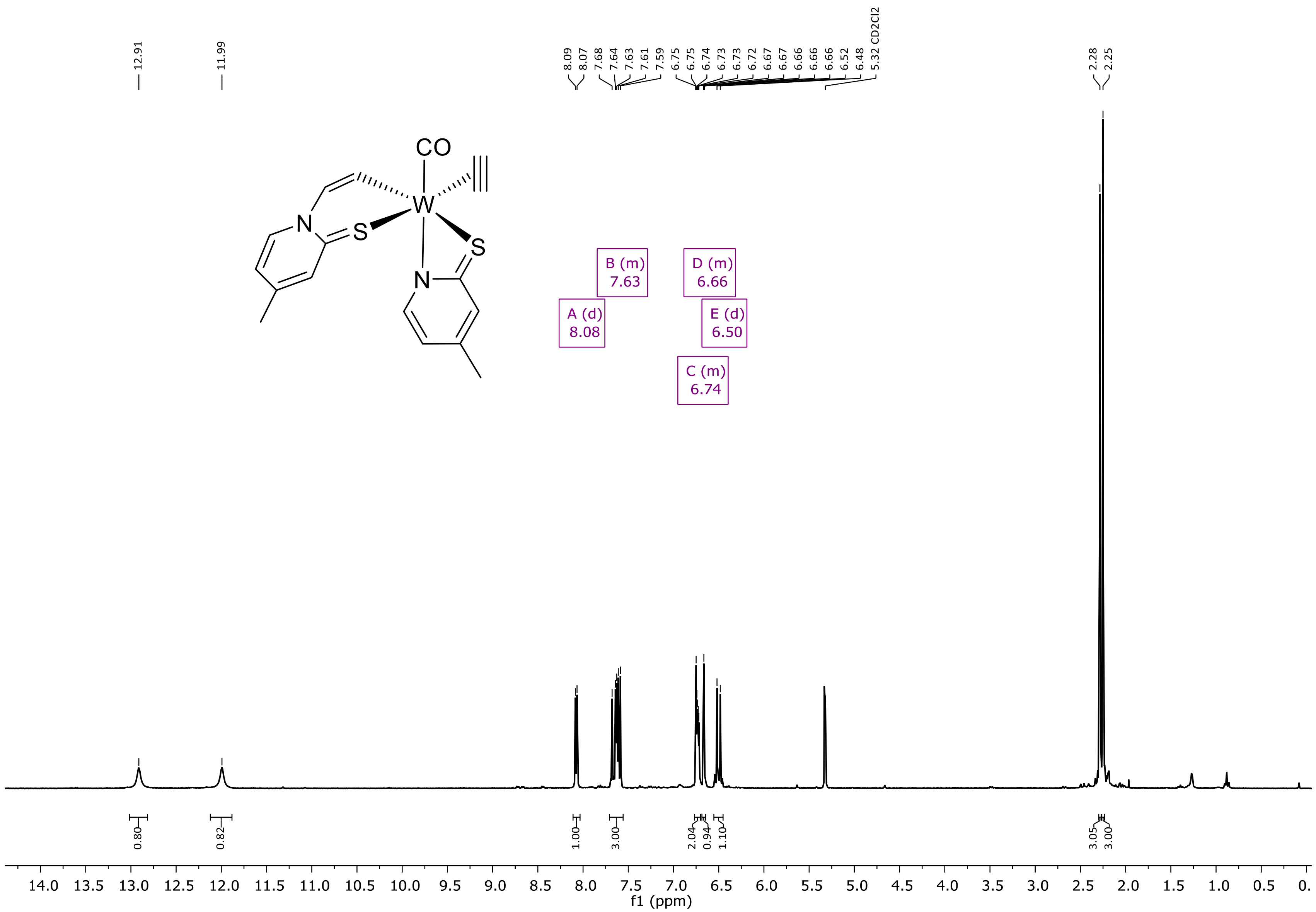

Figure S22. ^{1}H NMR spectrum of $[W(CO)(C_2H_2)(4\text{-MePyS})(CHCH\text{-}4\text{-MePyS})]$ (**3a**) in CD_2Cl_2.

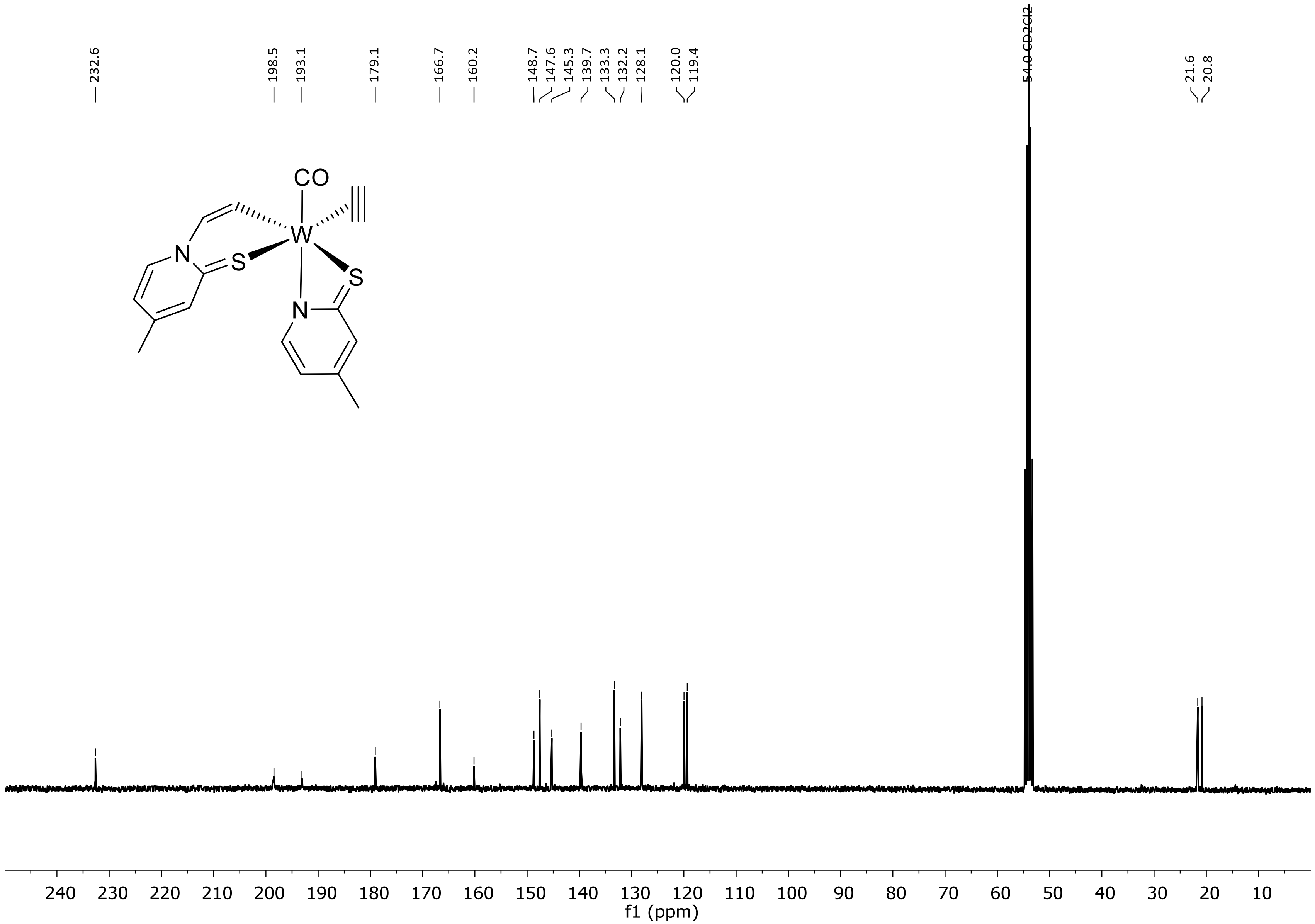

Figure S23. ^{13}C NMR spectrum of [W(CO)(C_2H_2)(4-MePyS)(CHCH-4-MePyS)] (**3a**) in CD_2Cl_2.

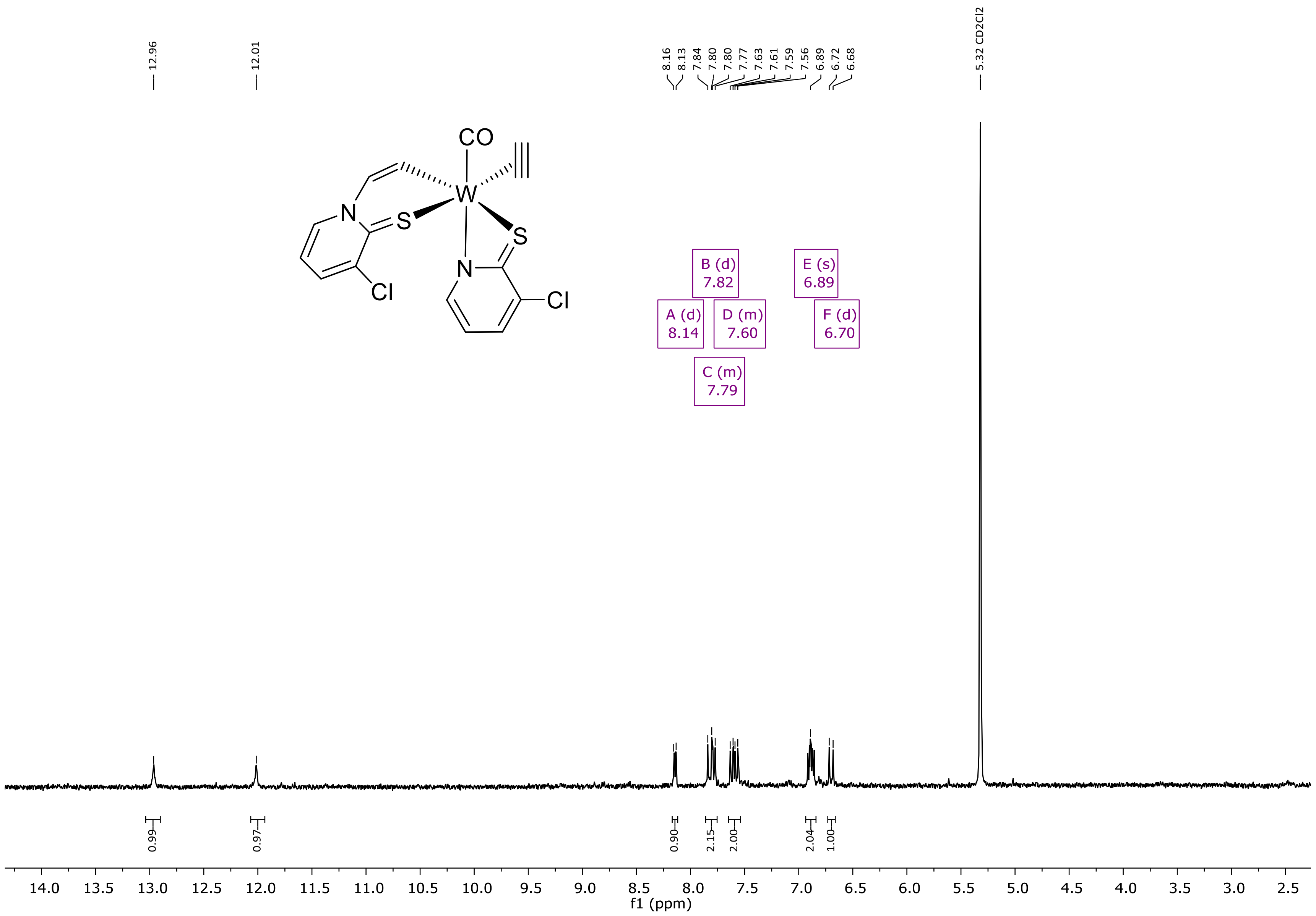

Figure S24. ^{1}H NMR spectrum of [W(CO)(C_2H_2)(3-ClPyS)(CHCH-3-ClPyS)] (**3b**) in CD_2Cl_2.

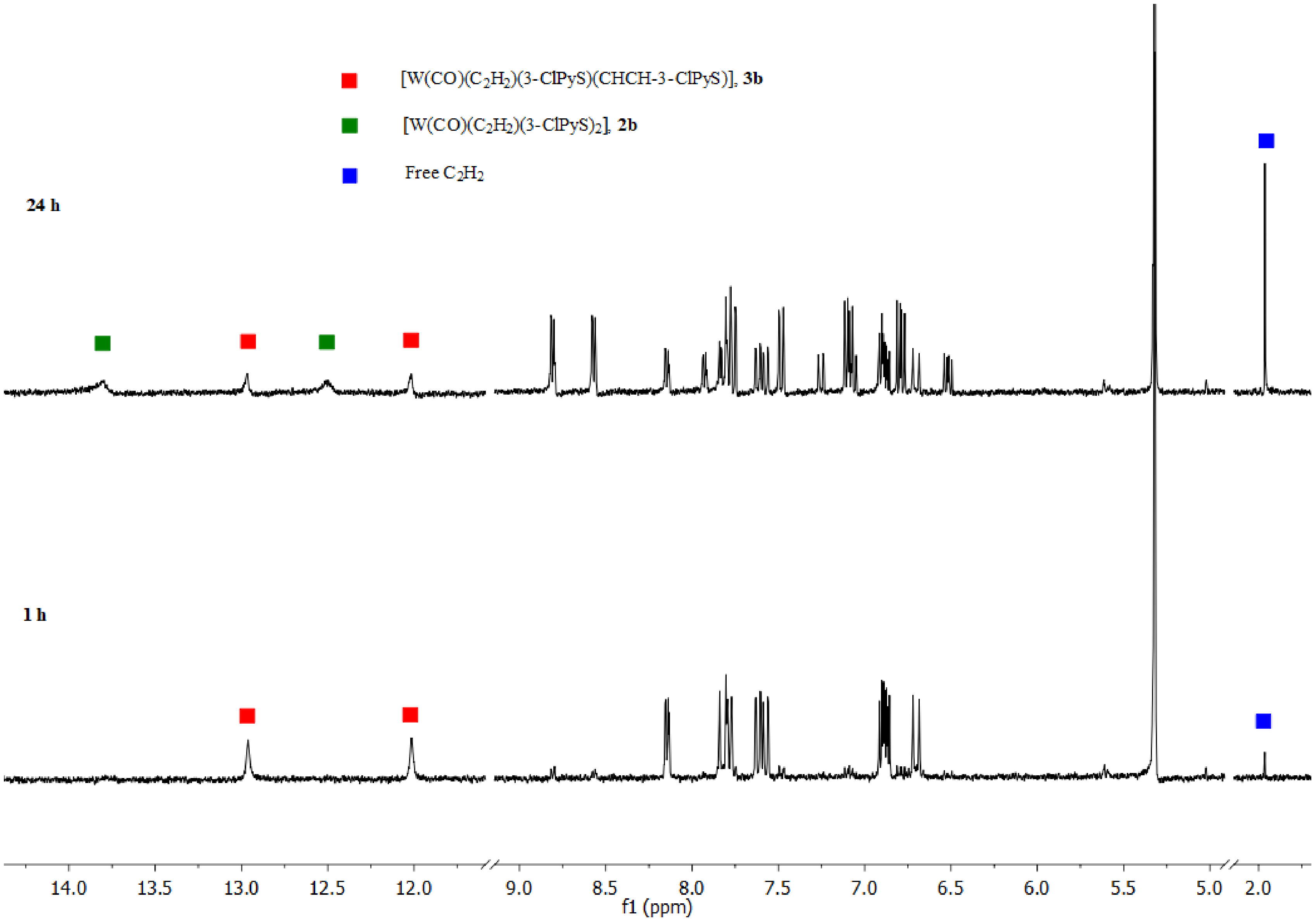

Figure S25. ^{1}H NMR spectra showing the partial formation of **2b** from **3b** due to the partial release of C_2H_2 in CD_2Cl_2 in a J-Young tube.

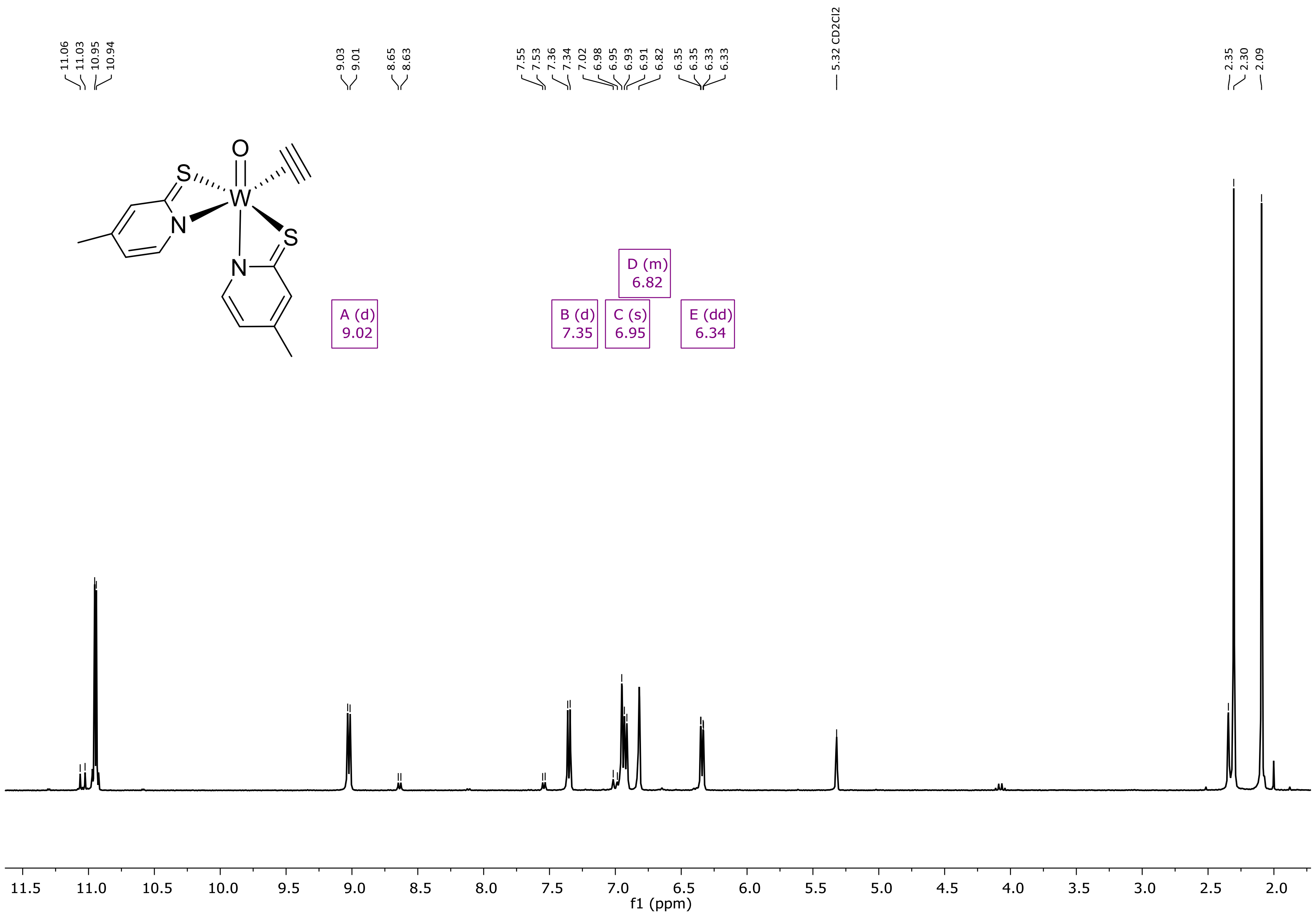

Figure S26. ^{1}H NMR spectrum of $[WO(C_2H_2)(4\text{-MePyS})_2]$ (**4a**) in CD_2Cl_2. Two isomers are present.

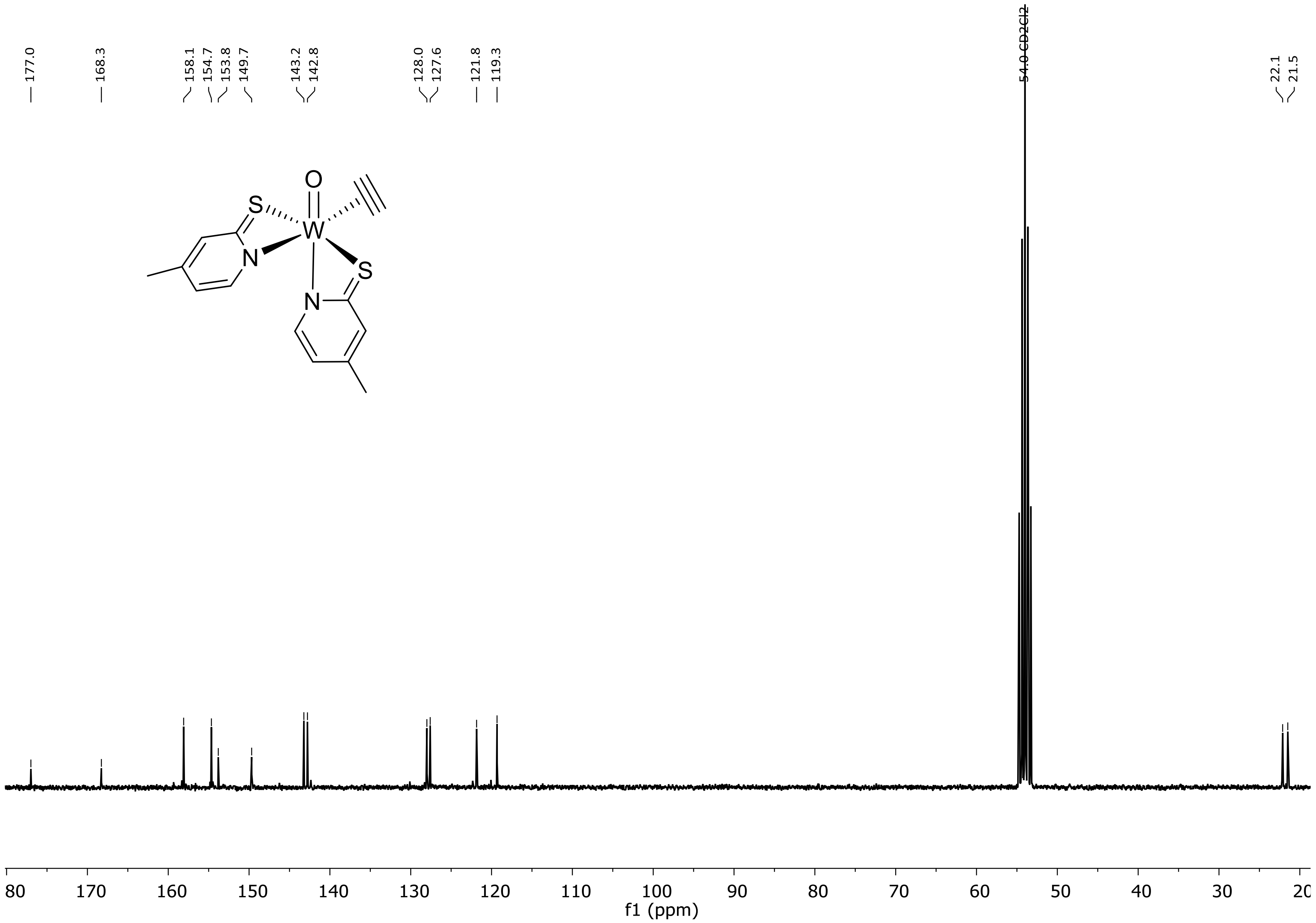

Figure S27. ^{13}C NMR spectrum of spectrum of [WO(C_2H_2)(4-MePyS)$_2$] (**4a**) in CD_2Cl_2. Two isomers are present.

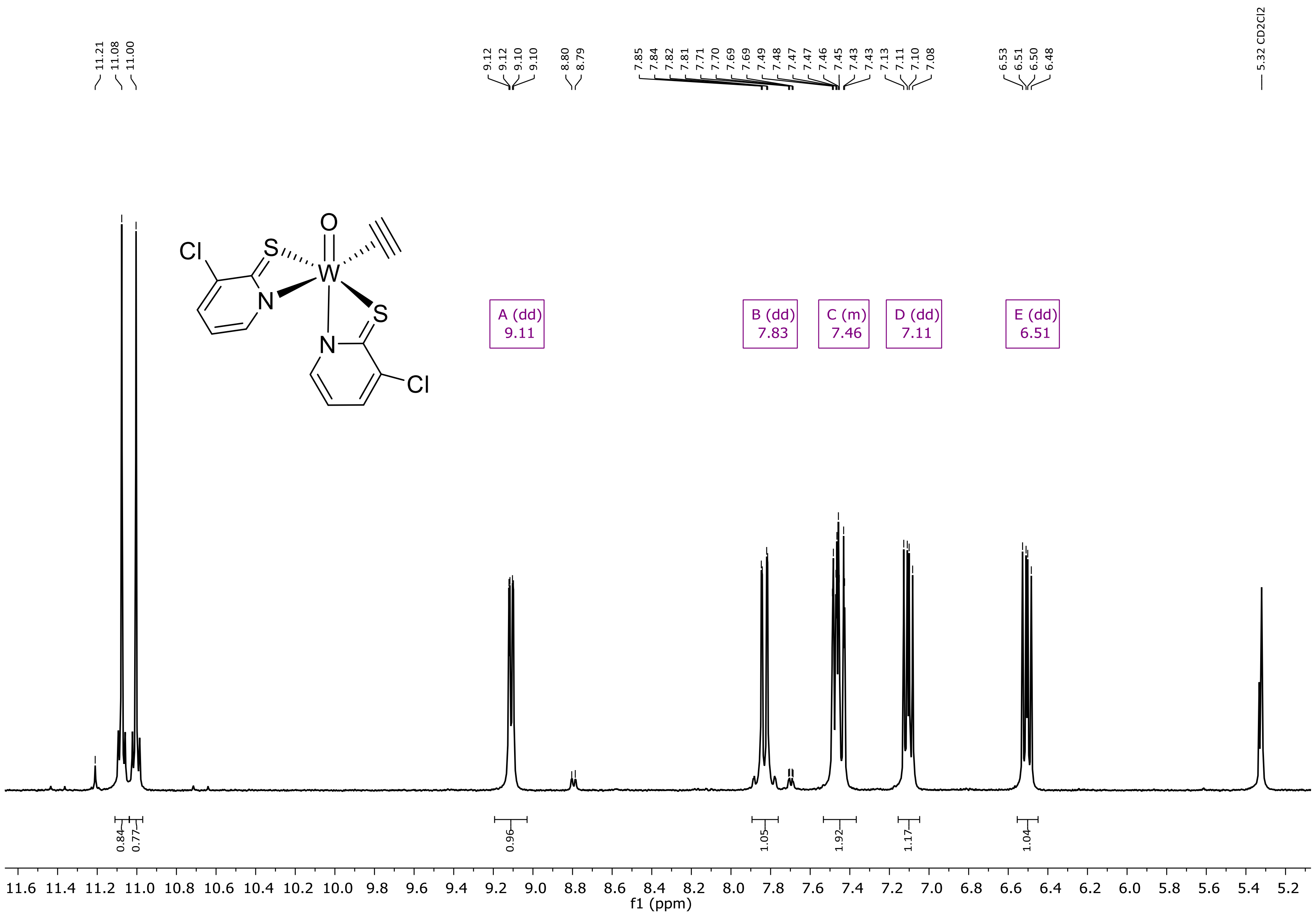

Figure S28. ^{1}H NMR spectrum of [$WO(C_2H_2)(3\text{-}ClPyS)_2$] (**4b**) in CD_2Cl_2. Two isomers are present.

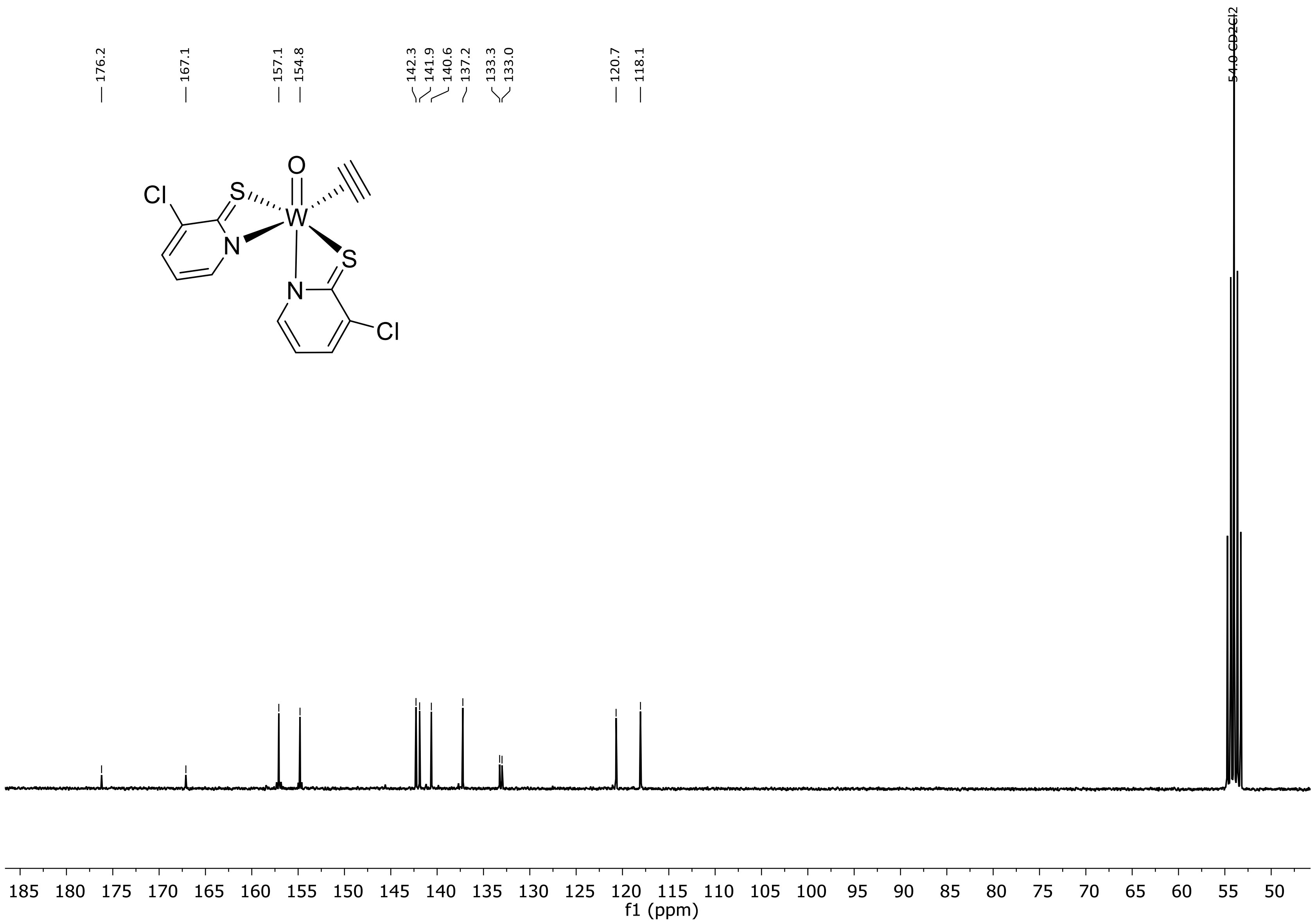

Figure S29. ^{13}C NMR spectrum of $[WO(C_2H_2)(3\text{-}ClPyS)_2]$ (**4b**) in CD_2Cl_2.

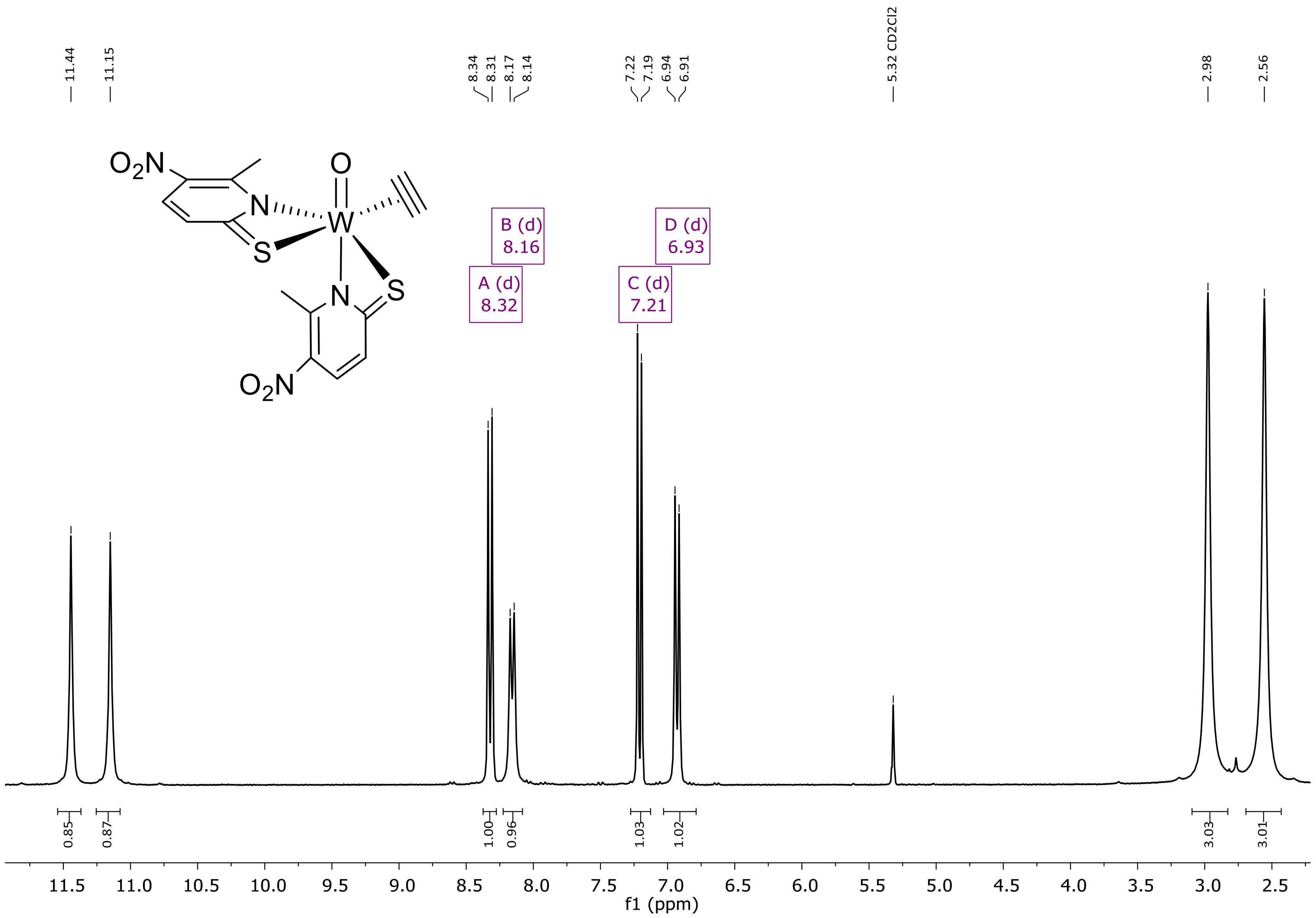

Figure S30. ^{1}H NMR spectrum of [WO(C_2H_2)(5-NO_2-6-MePyS)$_2$] (**4c**) in CD_2Cl_2.

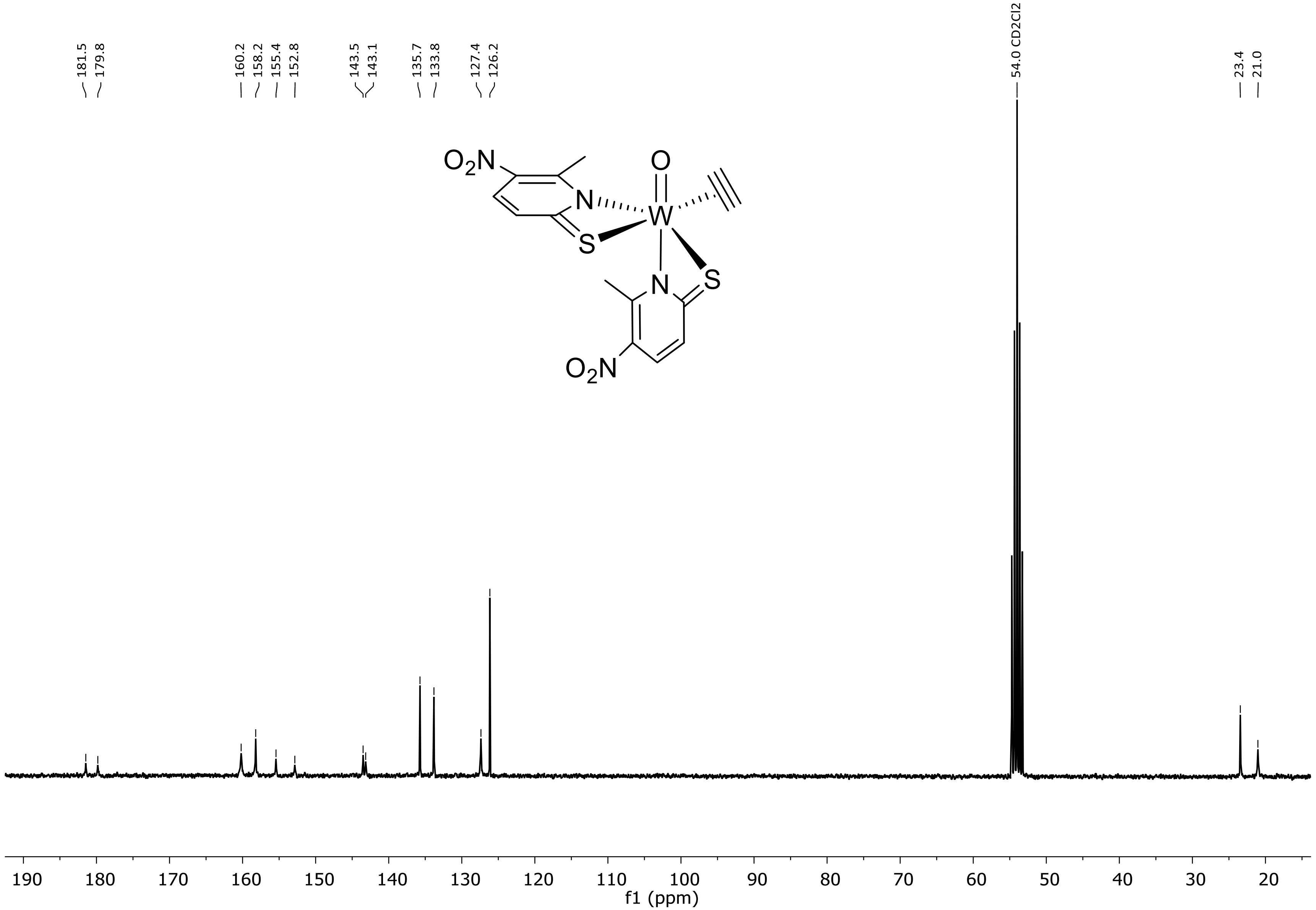

Figure S31. ^{13}C NMR spectrum of $[WO(C_2H_2)(5\text{-}NO_2\text{-}6\text{-}MePyS)_2]$ (**4c**) in CD_2Cl_2.

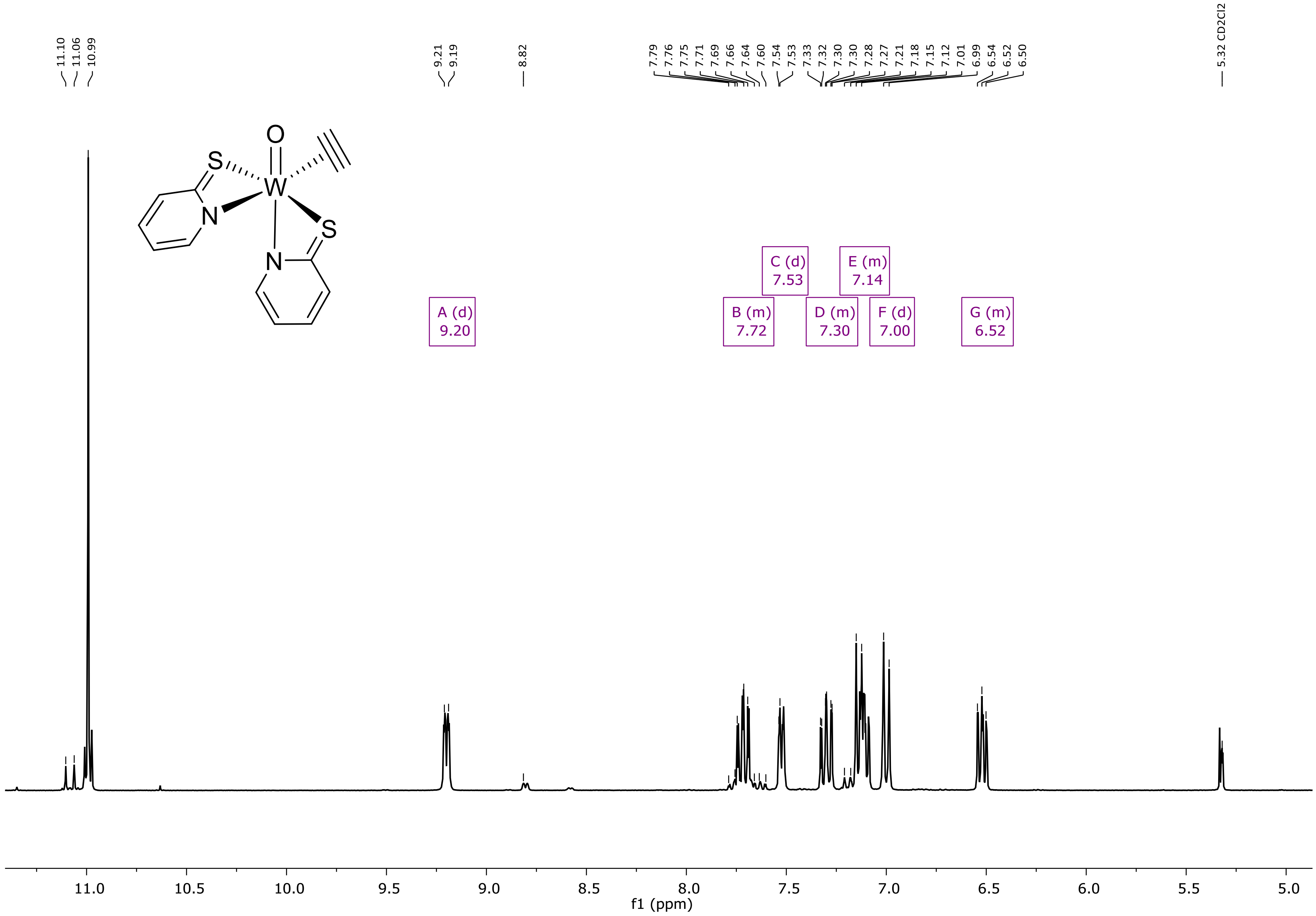

Figure S32. ^{1}H NMR spectrum of $[WO(C_2H_2)(PyS)_2]$ (**4d**) in CD_2Cl_2. Two isomers are present.

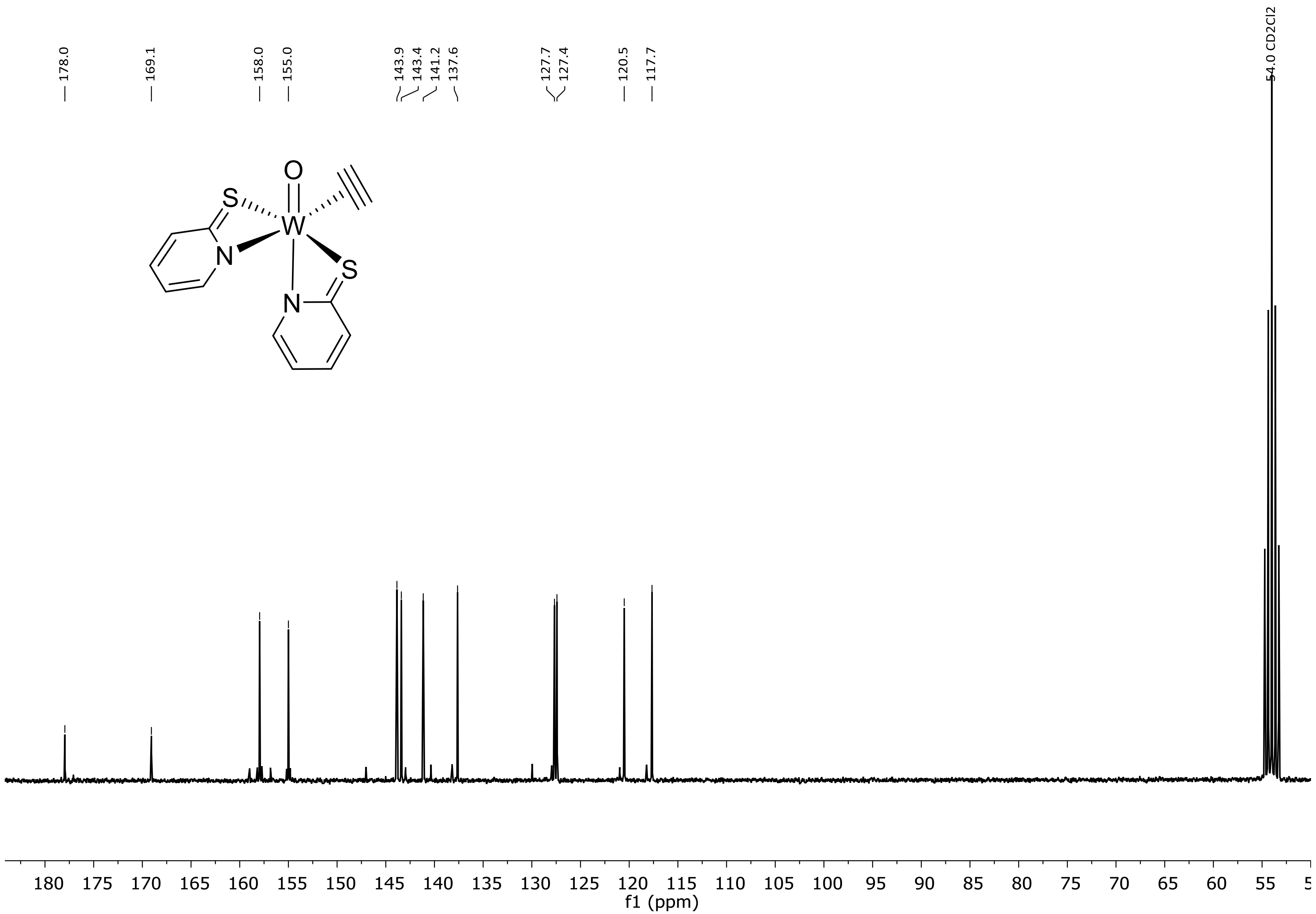

Figure S33. ^{13}C NMR spectrum of [WO(C_2H_2)(PyS)$_2$] (**4d**) in CD_2Cl_2. Two isomers are present.

5 References

(1) Peschel, L. M.; Schachner, J. A.; Sala, C. H.; Belaj, F.; Mösch-Zanetti, N. C. An Update on W II and Mo II Carbonyl Precursors and Their Application in the Synthesis of Potentially Bio-Inspired Thiophenolate-Oxazoline Complexes. *Z. anorg. allg. Chem.* **2013**, *639*, 1559–1567.

(2) Vidovič, C.; Peschel, L. M.; Buchsteiner, M.; Belaj, F.; Mösch-Zanetti, N. C. Structural Mimics of Acetylene Hydratase: Tungsten Complexes Capable of Intramolecular Nucleophilic Attack on Acetylene. *Chem. - Eur. J.* **2019**, *25*, 14267–14272.

(3) Kanishchev, O. S.; Dolbier, W. R. Synthesis and characterization of 2-pyridylsulfur pentafluorides. *Angew. Chem., Int Ed.* **2015**, *54*, 280–284.

(4) Romines, K. R.; Freeman, G. A.; Schaller, L. T.; Cowan, J. R.; Gonzales, S. S.; Tidwell, J. H.; Andrews, C. W.; Stammers, D. K.; Hazen, R. J.; Ferris, R. G.; Short, S. A.; Chan, J. H.; Boone, L. R. Structure-activity relationship studies of novel benzophenones leading to the discovery of a potent, next generation HIV nonnucleoside reverse transcriptase inhibitor. *J. Med. Chem* **2006**, *49*, 727–739.

(5) Ehweiner, M. A.; Wiedemaier, F.; Belaj, F.; Mösch-Zanetti, N. C. Oxygen Atom Transfer Reactivity of Molybdenum(VI) Complexes Employing Pyrimidine- and Pyridine-2-thiolate Ligands. *Inorg. Chem.* **2020**, *59*, 14577–14593.

(6) Sheldrick, G. M. A short history of SHELX. *Acta Cryst. A* **2008**, *64*, 112–122.

(7) Sheldrick, G. M. Crystal structure refinement with SHELXL. *Acta Cryst. C* **2015**, *71*, 3–8.
